# Identification of Novel Alleles of the Rice Blast-Resistance Gene *Pi9* through Sequence-Based Allele Mining

**DOI:** 10.1186/s12284-020-00442-z

**Published:** 2020-12-07

**Authors:** Ying Zhou, Fang Lei, Qiong Wang, Weicong He, Bin Yuan, Wenya Yuan

**Affiliations:** 1 College of Life Science and Health, Wuhan University of Science and Technology, Wuhan, 430065 People’s Republic of China; 2 Institute of Model Animal of Wuhan University, Basic Medical School of Wuhan University, Wuhan, 430071 People’s Republic of China; 3 Key Laboratory of Integrated Management of Crops of Central China, Ministry of Agriculture, Wuhan, 430064 People’s Republic of China; 4 Hubei Key Laboratory of Crop Disease, Insect Pests and Weeds Control, Wuhan, 430064 People’s Republic of China; 5 College of Life Sciences, Hubei University, Wuhan, 430062 People’s Republic of China

**Keywords:** Gene conversion, Nucleotide diversity, *Pi9*, R genes, Resistance gene alleles, Rice blast

## Abstract

**Background:**

As rice (*Oryza sativa*) is the staple food of more than half the world’s population, rice production contributes greatly to global food security. Rice blast caused by the fungus *Magnaporthe oryzae* (*M. oryzae*) is a devastating disease that affects rice yields and grain quality, resulting in substantial economic losses annually. Because the fungus evolves rapidly, the resistance conferred by most the single blast-resistance genes is broken after a few years of intensive agricultural use. Therefore, effective resistance breeding in rice requires continual enrichment of the reservoir of resistance genes, alleles, or QTLs. Seed banks represent a rich source of genetic diversity; however, they have not been extensively used to identify novel genes and alleles.

**Results:**

We carried out a large-scale screen for novel blast-resistance alleles in 1883 rice varieties from major rice-producing areas across China. Of these, 361 varieties showed at least moderate resistance to natural infection by rice blast at rice blast nurseries in Enshi and Yichang, Hubei Province. We used sequence-based allele mining to amplify and sequence the allelic variants of the major rice blast-resistance genes at the *Pi2*/*Pi9* locus of chromosome 6 from the 361 blast-resistant varieties, and the full-length coding region of this gene could be amplified from 107 varieties. Thirteen novel *Pi9* alleles (named *Pi9*-Type1 to *Pi9*-Type13) were identified in these 107 varieties based on comparison to the *Pi9* referenced sequence. Based on the sequencing results, the *Pi2/Pi9* locus of the 107 varieties was divided into 15 genotypes (including three different genotypes of *Pi9*-Type5). Fifteen varieties, each representing one genotype, were evaluated for resistance to 34 *M. oryzae* isolates. The alleles from seven varieties with the highest resistance and widest resistance spectra were selected for transformation into the susceptible variety J23B to construct near-isogenic lines (NILs). These NILs showed resistance in a field test in Enshi and Yichang, indicating that the seven novel rice blast-resistance tandem-repeat regions at the *Pi2/Pi9* locus of chromosome 6 could potentially serve as a genetic resource for molecular breeding of resistance to rice blast.

**Conclusions:**

The thirteen novel *Pi9* alleles identified in this study expand the list of available of blast-resistance alleles. Seven tandem-repeat regions of the *Pi2/Pi9* locus from different donors were characterized as broad-spectrum rice blast-resistance fragments; these donors enrich the genetic resources available for rice blast-resistance breeding programs.

**Supplementary Information:**

The online version contains supplementary material available at 10.1186/s12284-020-00442-z.

## Introduction

Rice blast is an acute, destructive disease that can reduce yields or even ruin an entire harvest. Grain blast also affects the quality of rice and poses a serious problem for food safety (Deng et al., 2017; Ishihara et al., 2014). In China, the disease affects more than 3.8 million hectares per year, reducing rice yield by 1 billion kg annually (Jiang et al., 2015; Tian et al., 2016). Rice blast, which is caused by the fungus *M. oryzae*, is the most devastating disease affecting rice under high temperature and humidity conditions, which favor its spread (Shen et al., 2004; Wang et al., 2017). Rice blast has been reported in almost all rice-producing areas worldwide, including the main rice-producing areas of 85 countries and regions (Miah et al., 2013; (Ballini et al, 2008).

Effective host resistance, conferred by resistance (R) genes, is considered to be the most economical approach to control plant diseases (Xiao et al., 2017; Wang & Valent, 2017). To date, more than 100 rice-blast R genes have been isolated (Hua et al., 2012; Zhao et al., 2018). Although analyzing these genes has advanced our understanding of the molecular mechanisms underlying disease resistance, maintaining genetic resistance in rice is challenging because single rice varieties are grown over large areas in monoculture and the pathogen evolves quickly. *M. oryzae* is known for its genetic instability and pathogenic variability, leading to rapid breakdown of resistance in rice varieties (Bryan et al., 2000; Jiang et al., 2012). Resistant rice varieties often remain effective for only a few years before new dominant pathogenic races of the fungus emerge (Lee et al., 2009; Li et al., 2017).

Plants have evolved various mechanisms that protect them from pathogen invasion and colonization. R genes encode receptors containing a nucleotide-binding site and leucine-rich repeats (NBS-LRR). Most R genes are organized into tight clusters containing multiple gene copies. Nine of the 13 major rice blast R-genes are clustered (Qu et al., 2006; Wu et al., 2012) and most are broad-spectrum R genes with variable resistance. The tandem-repeat region of the *Pi2/Pi9* locus contains at least six known R genes (*Pi2*, *Pi9*, *Piz-t*, *Piz*, *Pigm*, and *Pi50*) and is situated close to the centromere of chromosome 6. Four R genes have been cloned in these regions (Dai et al. 2010; Zhou et al., 2006), divided into *Pi2* locus and *Pi9* locus. The *Pi2* locus includes the R genes *Pi2*, *Pigm* and *Piz-t*, whereas the *Pi9* locus includes the *Pi9* R gene (Xiao et al., 2017). Numerous studies have indicated that the clustered arrangement of R genes has contributed to the evolution of novel resistance specificities via gene conversion, recombination, or unequal crossing over (Ashikawa et al., 2008; Dai et al., 2010). Some NBS-LRR gene homologs at the same locus exhibit a different evolutionary pattern. Genomic analysis of the *Pi9* locus in various rice cultivars and wild rice lines has shown that the copy numbers and SNP genotypes of *Pi9* homologs vary, pointing to the complex evolutionary history of this R-gene locus (Wu et al., 2012).

Here, to gain insight into the origin and evolution of this locus, and to identify alleles with broad-spectrum resistance for use in molecular breeding, we analyzed the genomic sequences of the tandem-repeat region in 361 blast-resistant rice varieties. These 361 varieties were selected from a collection of 1883 varieties grown throughout China and were resistant to rice blast in at least one rice planting area. The tandem-repeat region of the *Pi2/Pi9* locus contains at least four R genes that have been cloned (*Pi2*, *Pi9*, *Piz-t* and *Pigm*) (Dai et al. 2010; Zhou et al., 2006). The *Pi2/Pi9* locus includes *Pi2*, *Pigm*, *Piz-t*, and the *Pi9* locus includes *Pi9* (Xiao et al., 2017). We reasoned these two loci might be functional sites for blast-resistance. Since we observed no alleles at the *Pi2* locus aside from the previously the cloned genes *Pi2*, *Pigm*, and *Piz-t*, we focused on the *Pi9* alleles, as this locus is a functional site that could represent the characteristics of these tandem-repeat regions.

We identified 13 novel *Pi9* alleles (including the three types of *Pi9*-Type5 alleles) in these 361 resistant rice varieties. We sequenced varieties carrying the novel alleles and compared them with the referenced sequence of *Pi9* gene, concluding that 13 alleles were novel. We inoculated varieties carrying the novel alleles with *M. oryzae* and observed that the *Pi9*-Type3/4/5/6/9/10/11 alleles conferred broad-spectrum rice-blast-resistance. The identification of these novel alleles broadens our knowledge on *Pi9*-like gene family and enriches the genetic resources available for rice blast-resistance breeding and for research into the molecular mechanisms underlying rice-blast interactions. We constructed NILs containing the novel *Pi9*-Type3/4/5/6/9/10/11 alleles to exclude the interference of the R genes at other sites. The NILs harboring the individual *Pi9*-Type3/4/5/6/9/10/11 alleles all showed resistance to rice-blast diseases in field trials in Enshi and Yichang, suggesting that these seven novel *Pi9* alleles might account for the genetic variation in rice-blast-resistance. We plan to introduce these novel broad-spectrum resistance alleles into high-quality rice varieties by molecular breeding to develop elite rice varieties with enhanced blast-resistance.

## Materials and Methods

### Plant Materials

Approximately 1883 rice cultivars, including *indica* and *japonica* types, were obtained from major rice-growing provinces in China and then maintained at Huazhong Agricultural University. Rice blast-resistant varieties were identified through natural inducement in two uniform rice-blast nurseries located at Enshi and Yichang in Hubei province. For each plant, the most seriously infected leaf was scored for each plant, as determined by using the HR-HS (HR-R-MR-MS-S-HS) scale rating system (Zhou et al., 2018), in which scores of HR-MR indicate an incompatible (resistant) reaction and scores of MS-HS indicate a compatible (susceptible) reaction,.varieties that were resistant to field mix-inoculum were selected for molecular screening. Lijiangxin Tuan Heigu (LTH), which is highly susceptible to rice blast, was used as a control for disease evaluation.

### Pathogen Collection, Inoculation and Disease Evaluation

For resistance spectrum analysis, we used 34 blast isolates of different races and virulence levels. These isolates, which were collected from across the major rice-growing provinces of China, are genetically distinct and belong to different blast lineages (Shen et al. 2004; Sasaki, 1922). We used these isolates to analyze phenotypes of parents with disease resistance genes. The 34 *M. oryzae* isolates, which are highly virulent on most of the rice lines, were also used for phenotypic analysis of the *Pi9* allele. These 34 *M. oryzae* isolates were sequenced and analyzed for *AvrPi9* and *AvrPiz-t*. The promoter and coding regions of *AvrPi9* and *AvrPiz-t* genes were sequenced and compared with reference sequences. The sequencing primers used are shown in Table S6.

Twelve-day-old seedlings were spray-inoculated with blast spore suspensions (approximately 1 × 10^5^ spores/mL) and grown in a dark chamber for 24 h (26 °C, 90% humidity). Subsequently, the growth conditions were changed to 12 h light/12 h of darkness. At 7 days post inoculation, the disease reaction (0–9 disease rating scale) of each line was recorded (IRRI, 2002).

### PCR for Allele Mining and Blast-Resistance Genes

The tandem-repeat region of the *Pi2/Pi9* locus contains two functional loci, *Pi9* and *Pi2* (including *Pi2*, *Pigm* and *Piz-t*). The BAC clone sequence of *O. sativa* cv. 75–1-127 (DQ285630.1, containing *Pi9*) was used as the *Pi9* locus referenced sequence, and the genomic sequences of *O. sativa* cv. Nipponbare (www.ncbi.nlm.nih.gov), MH63 and ZS97 (http://rice.hzau.edu.cn/cgi-bin/rice2/blast) varieties were used as the negative referenced sequences. RiceVarMap v2.0 (http://ricevarmap.ncpgr.cn/wwwblast/blast1.html) was used to design PCR primers for the *Pi9* alleles (Zhao et al., 2015) so that the primers could only amplify the functional *Pi9* orthologues in resistant varieties and could not produce amplification PCR products from the negative reference varieties. Three pairs primers with successful amplification and covering the full-length *Pi9* except for a gap region in intron1 were used for the *Pi9* alleles. The primer designed at the *Pi2* locus was similar to that of the *Pi9* locus. The BAC clone sequences of *O. sativa* cv. GM4H (KU904633.2, containing *Pigm*) and C101A51 (DQ454158.1, containing *Pi2*) and *O. sativa* cv. ZY1H (DQ352040, containing *Piz-t*) were used as referenced sequences for the *Pi2* locus. The primers for amplification or sequnencing are given in Table S1.

Alleles were PCR amplified using genomic DNA extracted from rice leaves using the CTAB method (Murray and Thompson 1980). The 50 μL reaction mix used for PCR included 50 ng of template DNA, 0.2 μM of both forward and reverse primers, 5 μL 10× LA Taq Buffer II, 8 μL dNTP Mixture (2.5 mM each), and 2.5 U TaKaRa LA Taq (RR02MQ, TAKARA). The primer sequences are listed in Table S1. Amplified PCR products were purified and sequenced using a Sanger’s method-based ABI 3730XL DNA Analyzer Sequencer (ABI, Applied Biosystems Amersham, USA). Three PCR amplifications were performed per fragment, and each PCR product was sequenced. When the three sequencing results were consistent, the sequence information was utilized. Otherwise, PCR amplification and sequencing were repeated.

### DNA Sequence Analysis

All the sequence reads generated for each allele by sequencing primers were assembled separately for each allele by using Sequencing Analysis Software Version 5.1 (Applied Biosystems). High-quality sequences were assembled, and the assembled DNA sequence of each allele was used to perform Blast2Sequences (https://blast.ncbi.nlm.nih.gov/Blast.cgi) analysis against the *Pi9* genes to check its similarity.

SNPs and InDels were identified based on the *Pi9* sequence as referenced sequences. For SNPs and InDels searches, multiple sequence alignment was performed for all alleles (along with *Pi9* alleles from 75 to 1-127 as reference) using Sequencer’s software. When analyzing genome sequence of the novel *Pi9* alleles identified in this study, the gap region in intron1 was replaced with the corresponding region of the reference *Pi9* gene. Multiple sequence alignment of DNA sequences from amplified gene fragments and the referenced sequences (including *Pi9*, *Pi2*, *Piz-t*, *Pigm*, and all NBS disease resistance genes in this region) was performed using CLUSTALW (http://www.ebi.ac.uk/Tools/msa/clustalw2/) and MEGA5.0 (http://www.megasoftware.net) softwares. The parameters used for MEGA 5.0 were bootstrap (1000 replications) and neighbour joining with the p-distance model. A phylogenetic tree was constructed with MEGA to analysis the evolution of the *Pi9* alleles. The average nucleotide diversity (π), average nucleotide polymorphism (θ), and Tajima’s D significance test values were used for calculating variation among all the alleles isolated from different varieties using DnaSP 5.0 software (Librado and Rozas 2009). Structural analyses of Pi9 alleles were performed to predict protein structure and conserved domains using CDD software (https://www.ncbi.nlm.nih.gov/Structure/cdd/wrpsb.cgi), and the data were recorded as their position. Various domains, such as coiled-coil domain (CC), nucleotide-binding site (NBS), and leucine-rich repeats (LRR), which play vital roles in disease resistance, were shown on the protein sequences of all alleles using the customized R scripts.

### Crossing and Selection Scheme

Plants harboring the *Pi9* alleles were crossed with J23B, and the F1 hybrids were backcrossed with J23B to obtain the BC1F1 populations. Markers closely linked with the *Pi9* resistance genes were used to check the corresponding *Pi9* alleles in the above BC1F1 populations (Table S2). Six plants displaying closest phenotypic resemblance to J23B and including the target *Pi9* allele from each BC1F1 populations were selected and their genetic backgrounds profiled using RICE6K (with 5102 SNP and InDel markers), a whole-genome SNP array (Yu et al. 2014). Only one plant with the target *Pi9* allele and background closest to J23B was selected to backcross with J23B up to BC2F1 generation. Similarly, BC3F1 was obtained from BC2F1 populations by backcrossing the plant with the target gene and the background closest to J23B. After selfing, the BC1F2, BC2F2 and BC3F2 populations were obtained and used to evaluate the effects of individual *Pi9* alleles in J23B backgrounds.

### Scoring Rice Blast-Resistance

The BC3F2 families and control varieties were planted in a randomized complete block design in 2017 in Enshi and Yichang, Hubei Province, China. Enshi and Yichang are both mountainous areas with high humidity and heavy fog. The tests were performed in three replications. In each replication, each plot consisted of 4 rows with 6 plants per row at a planting density of 16 cm between plants and 16 cm between rows. To adequately induce blast disease infection, LTH was planted at both sides of each row and around the population. Field management essentially followed normal agricultural practices except that bactericides were not utilized.

All the plants were scored for leaf blast severity at the tillering stage and for neck blast severity at maturity stage using the HR-HS (HR-R-MR-MS-S-HS) scale rating system (IRRI, 2002; Zhou et al., 2018). The most seriously infected leaf among the top two or three new leaves was scored for each plant at the tillering stage as the leaf blast rate. The percentage of infection on the neck was scored for each plant at physiological maturity stage as neck blast rate.

## Results

### Selection of Rice Varieties for *Pi9* Alleles Mining

We collected 1883 rice varieties originating from regions across China, including 729 farm-cultivated varieties, 485 varieties with core germplasm resources, 514 *japonica* varieties from northern China, and 155 varieties from other sources. To evaluate the blast-resistance of these varieties in regional trials, plants were grown at Enshi and Yichang, Hubei Province, where test nurseries have been established. We classified the disease resistance of the cultivars into six categories: HR (High Resistance) to HS (High Sensitivity). We identified 361 varieties that displayed HR or R phenotypes in Enshi or Yichang.

Four established broad-spectrum R-genes, *Pigm, Pi2*, *Pi9* and *Piz-t* are located in the same ~ 10.38-Mb region on the short arm of chromosome 6. In the tandem-repeat region of the *Pi2*/*Pi9* locus, two functional copies had been cloned in this tandem-repeat region: the *Pi2* locus and *Pi9* locus. The *Pi2* locus, including *Pi2*, *Pigm*, and *Piz-t*, were specifically amplified from 55 of the 361 resistant varieties using the set of *Pi2* primers (Table S1). No novel allele was identified at this locus; the only R genes present were the known genes *Pi2*, *Pigm*, and *Piz-t*. The *Pi9* locus was specifically amplified from 108 of the 361 resistant varieties.

### Isolation of *Pi9* Alleles

**Fig. 1:**
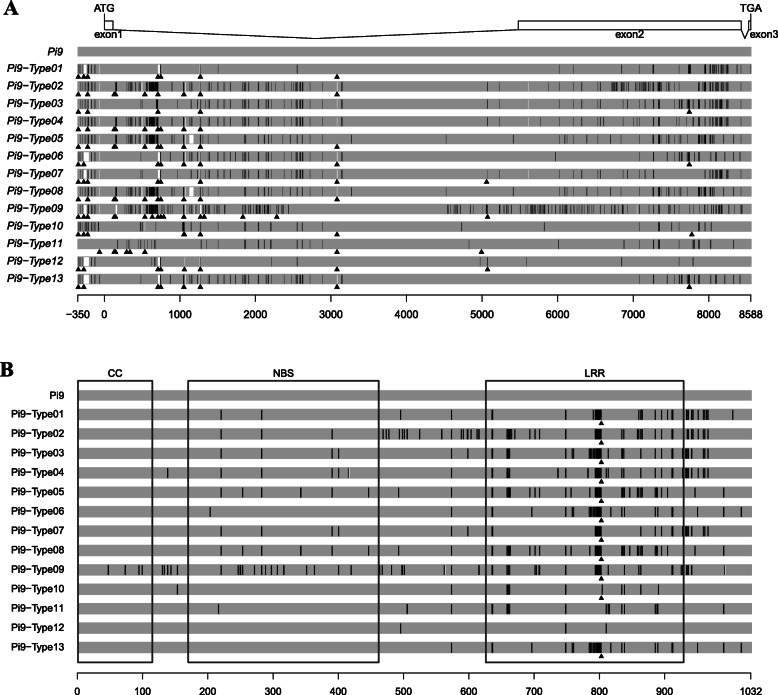
Schematic map for sequence alignment of newly identified *Pi9* allele gene (**a**) and protein (**b**)**. a**.Thirteen alleles of *Pi9* were isolated from the studied rice accessions and compared with *Pi9* gene. Boxes indicate exons and lines indicates introns, and the start codon and the termination codon are labeled with ATG and TGA, respectively, in the figure above. The unit scale indicates the location of nucleotides. **b** Thirteen alleles of Pi9 protein compared with Pi9 protein. The domain regions of the Pi9 alleles protein are shown as boxes (CC, NBS and LRR) in the picture. CC, coiled-coil domain; NBS, nucleotide-binding site; LRR, leucine-rich repeats. The unit scale indicates the location of amino acids.The black lines on the bar indicate the nucleotide or amino acid polymorphism compared with the reference sequence. The white strips indicate deletion, and the size of gaps indicate the length of deletion sequence. Triangles indicate insertion

*Pi9* genomic sequences of approximately 8938 bp, including a parts of the promoters (350 bp) and full-length coding regions (8588 bp), were amplified and sequenced from 108 (one variety could not been sequenced) of the 361 resistant varieties. Based on our analysis of these sequences, we identified 13 novel *Pi9* alleles from these 107 varieties (Table S3). The obtained sequences were compared with the reported *Pi9* gene sequences, specifically in the coding region. The 13 alleles contained unique SNPs, insertions, and deletions. Figure 1a shows the sequence alignment of the novel *Pi9* alleles.

The 13 novel *Pi9* alleles were sequenced using sequencing primers for *Pi2* (*Pi2, Piz-t*, and *Pigm* could be amplified with the same set of primers)*.* Based on the sequencing results, *Pi9*-Type5 was divided into three types (donors of *Pi9*-Type5-*Pi2*, *Pi9*-Type5-*Piz-t*, and *Pi9*-Type5 carried *Pi2*, *Piz-t*, and no cloned genes, respectively). In addition, the donor of *Pi9*-Type4 carried the *Pigm* gene, and the donor of *Pi9*-Type8 carried the *Piz-t* gene (Table S3).

### Sequence Analysis of the *Pi9* Alleles

Among the 13 *Pi9* alleles identified, *Pi9-*Type8 shared the lowest level of genomic sequence identity (92%) with the reference allele *Pi9*, and seven alleles had more than 99% identity (*Pi9*-Type1, *Pi9*-Type5, *Pi9*-Type6, *Pi9*-Type8, *Pi9*-Type10, *Pi9*-Type12, and *Pi9*-Type13) (Table 1). These 13 alleles differed from *Pi9* by numerous nucleotide polymorphisms, insertions, and deletions that were either unique or shared among the different alleles.

**Table 1:** Summary of SNP and different alleles of the *Pi9* gene in different species

***Pi9*** allele	Varieties	Number of accessions carrying the allele	Identity to ***Pi9***	Number of SNP sites	Number of SNP sites			Number of inserts/deletions	Number of inserts/deletions		
					Pt	Ex	In		Pt	Ex	In
*Pi9*	75–1-127	2	–	–	–	–	–	–	–	–	–
*Pi9*-Type1	Heo Trang	1	99%	79	29	47	3	17	9	0	8
*Pi9*-Type2	IR64	2	95%	443	246	94	103	259	10	0	249
*Pi9*-Type3	HC1H	13	98%	134	33	61	40	26	20	3	3
*Pi9*-Type4 ^a^	PIIB	11	96%	410	253	56	101	266	10	0	256
*Pi9*-Type5 ^a^	XS209	56	95%	451	259	57	135	320	9	0	311
*Pi9*-Type6	YD4038	1	99%	104	25	31	48	241	9	3	12
*Pi9*-Type7	KAUKKYI ANI	2	99%	105	30	36	39	23	20	0	3
*Pi9*-Type8 ^a^	DY1H	6	92%	710	519	56	135	581	9	0	572
*Pi9*-Type9	THAVALU	1	93%	608	312	103	193	342	109	3	230
*Pi9*-Type10	ZWH210	6	97%	316	282	16	18	269	259	0	10
*Pi9*-Type11	R03138	3	96%	387	339	22	26	339	234	0	105
*Pi9*-Type12	JP-5	1	99%	32	22	4	6	9	7	0	2
*Pi9*-Type13	ZD5H	2	99%	105	26	30	49	26	9	3	14

^a^*Pigm* is contained in the donor of *Pi9*-Type4; *Piz-t* is contained in the donor of *Pi9*-Type8 and a part of *Pi9*-Type5; *Pi2* is contained in the donor of a part of *Pi9*-Type5

*Pt* Promoter; *Ex* Exon; *In* Intron. Varieties, Representative donor variety containing the allele

The alleles include two large insertions/deletions in the nucleotide sequence between − 350 and 0 bp (considering the start codon as 0 bp) (Fig. 1b; Table S3). *Pi9*-Type11 has a 515-bp insertion at − 274 bp and a unique 232-bp insertion at − 64 bp (Fig. 1b; Table S4). In the first intron of the *Pi9*, we identified three large insertions located at + 150 bp (a 366-bp insertion), + 714 bp (a 126-bp insertion), and + 2290 bp (a 48-bp insertion). The insertion at + 150 bp was present in *Pi9*-Type2/4/5/8/9 and that at + 714 bp was present in *Pi9*-Type2/4/5/8 (Fig. 1b; Table S3). The insertion at + 2290 bp was only present in *Pi9*-Type9.

**Fig. 2:**
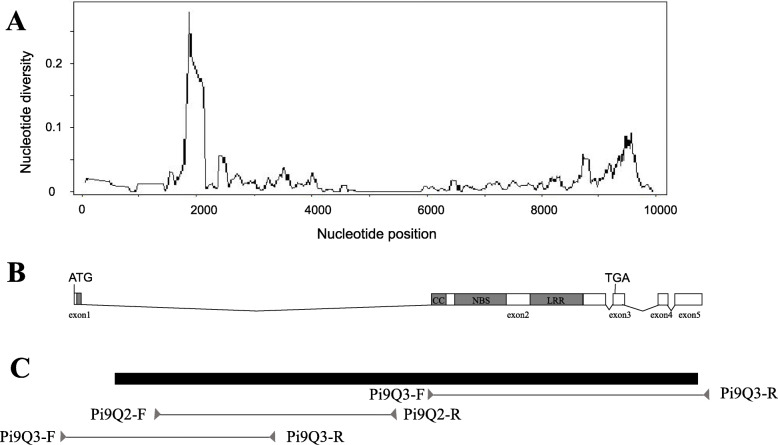
SNP rich region in the Pi9 alleles. **a**. Sliding-window analysis of nucleotide diversity (π) about new *Pi9* alleles are shown above; **b**. The gene structure is described at the unit scale of nucleotide location, and the domains (CC, NBS and LRR) are illustrated as gray frames; **c**. Schematic diagram of the location of the primers used to amplify the *Pi9* alleles. The unit scale indicates the location of nucleotides

All *Pi9* alleles have conserved sequences in the CC and NBS domains, suggesting that these domains are important for *Pi9* function. By contrast, in the LRR domain, the sequence of these alleles were highly polymorphic. The allelic variation in the LRR domain indicates that this region is under less selective pressure than the other domains (Fig. 2; Table 2). We also performed nucleotide polymorphism analyses for all 13 *Pi9* alleles using DnaSP5.10. The average nucleotide diversity (π) of the alleles was 0.01674. Sliding-window analysis of *Pi9* nucleotide diversity in the *Pi9* allele showed that the diversity rate was higher in regions with abundant nucleotide polymorphisms and that there were more deletions/insertions in the first intron than elsewhere in the allele (Fig. 2; Table 2). The Tajima D test value was less than 1 (− 0.64099), indicating that the *Pi9* locus is under positive selection, especially in the conserved CC and NBS domains (Fig. 2; Table 2).

**Table 2:** Summary of the natural variation of different *Pi9* allele genes in different species

Region	π	θ	Tajima’s D
CC	0.00670	0.01475	−2.23881
NBS	0.01035	0.01574	−1.49472
LRR	0.03311	0.03529	−0.27483
Promoter	0.01401	0.01823	−0.94844
Exon1	0	0	–
Intron1	0.01499	0.01665	−0.45014
Exon2	0.02137	0.02604	−0.80967
Intron2	0	0	–
Exon3	0	0	–

Nucleotide diversity (π), Watterson’s Theta (θ), and Tajima’s D for alleles

*CC* Coiled-coil domain; *NBS* Nucleotide-binding site; *LRR* Leucine-rich repeats

### Analysis of the Deduced Pi9 Protein Sequences

Pi9 proteins are composed of three conserved domains: CC, NBS, and LRR. All of the newly identified alleles have complete open reading frames (ORFs) similar to that of Pi9 (Fig. 1b; Table 3; Table S4). The alleles’ predicted encoded protein had high similarity with the control Pi9 protein sequence (94.0–99.7%), with the alleles containing complete CC-NBS-LRR domains. Of all the allele amino acid sequences, Pi9-Type9 had the most amino acid differences compared to the Pi9 amino acid sequence. In the CC domain, only Pi9-Type9 had amino acid differences (4 amino acids are different from the Pi9 amino acid sequence). In the NBS domain, there were no difference between Pi9-Type10/12/13 and Pi9 proteins. In the LRR region, there were more differences between the proteins predicted to be encoded by each allele type, of which Pi9-Type5/8 had the most differences, with 27 amino acid differences. Pi9-Type12 had the fewest differences, with only 2 amino acid differences. None of the 13 Pi9 allele proteins had amino acid insertions/deletions in the CC and NBS domains, and only one amino acid insertion was detected in the LRR domain in Pi9-Type1 ~ 10/13 (Table 3, Table S5).

**Table 3:** Summary of difference in each alleles of the Pi9 protein

Protein	Varieties	AA Number	Identity to Pi9	Number of SNP sites	Number of SNP sites			Number of inserts/deletions	Number of inserts/deletions		
					CC	NBS	LRR		CC	NBS	LRR
Pi9	75–1-127	1032	–	–	–	–	–	–	–	–	–
Pi9-Type1	Heo Trang	1032	97.0%	31	0	2	15	3	0	0	1
Pi9-Type2	IR64	1033	95.0%	52	0	3	23	3	0	0	1
Pi9-Type3	HC1H	1031	95.9%	40	0	4	26	3	0	0	1
Pi9-Type4	PIIB	1032	96.3%	36	0	5	18	2	0	0	1
Pi9-Type5	XS209	1033	96.4%	37	0	6	27	3	0	0	1
Pi9-Type6	YD4038	1032	97.4%	25	0	1	20	2	0	0	1
Pi9-Type7	KAUKKYI ANI	1032	97.9%	22	0	4	8	2	0	0	1
Pi9-Type8	DY1H	1030	96.4%	37	0	6	27	2	0	0	1
Pi9-Type9	THAVALU	1033	94.0%	58	4	15	19	3	0	0	1
Pi9-Type10	ZWH210	1032	98.6%	12	0	0	10	2	0	0	1
Pi9-Type11	R03138	1032	98.2%	19	0	1	15	0	0	0	0
Pi9-Type12	JP-5	1033	99.7%	3	0	0	2	1	0	0	0
Pi9-Type13	ZD5H	1032	97.5%	24	0	0	20	3	0	0	1

Varieties, Representative donor variety containing the alleles; *AA Number* Number of amino acids in Pi9 allele proteins; *CC* Coiled-coil domain; *NBS* Nucleotide-binding site; *LRR* Leucine-rich repeats

### Phylogeny and Distribution of the Novel *Pi9* Alleles

In addition to the cloned *Pi9* gene, we identified 13 *Pi9* alleles in this chromosomal region. The reference allele *Pi9* is derived from the 75–1-127 donor. The presence of other *Pi9* alleles varied among rice varieties. Four of the 13 novel *Pi9* alleles (i.e., *Pi9*-Type1, *Pi9*-Type6, *Pi9*-Type9, and *Pi9*-Type12) were present in only one rice line each. *Pi9*-Type2, *Pi9*-Type7, *Pi9*-Type11, and *Pi9*-Type13 were present in fewer than three varieties. The remaining alleles were present in more than six varieties (Table 1). Among the *Pi9* alleles, *Pi9*-Type5 was the most widespread and was detected in 56 varieties.

**Fig. 3:**
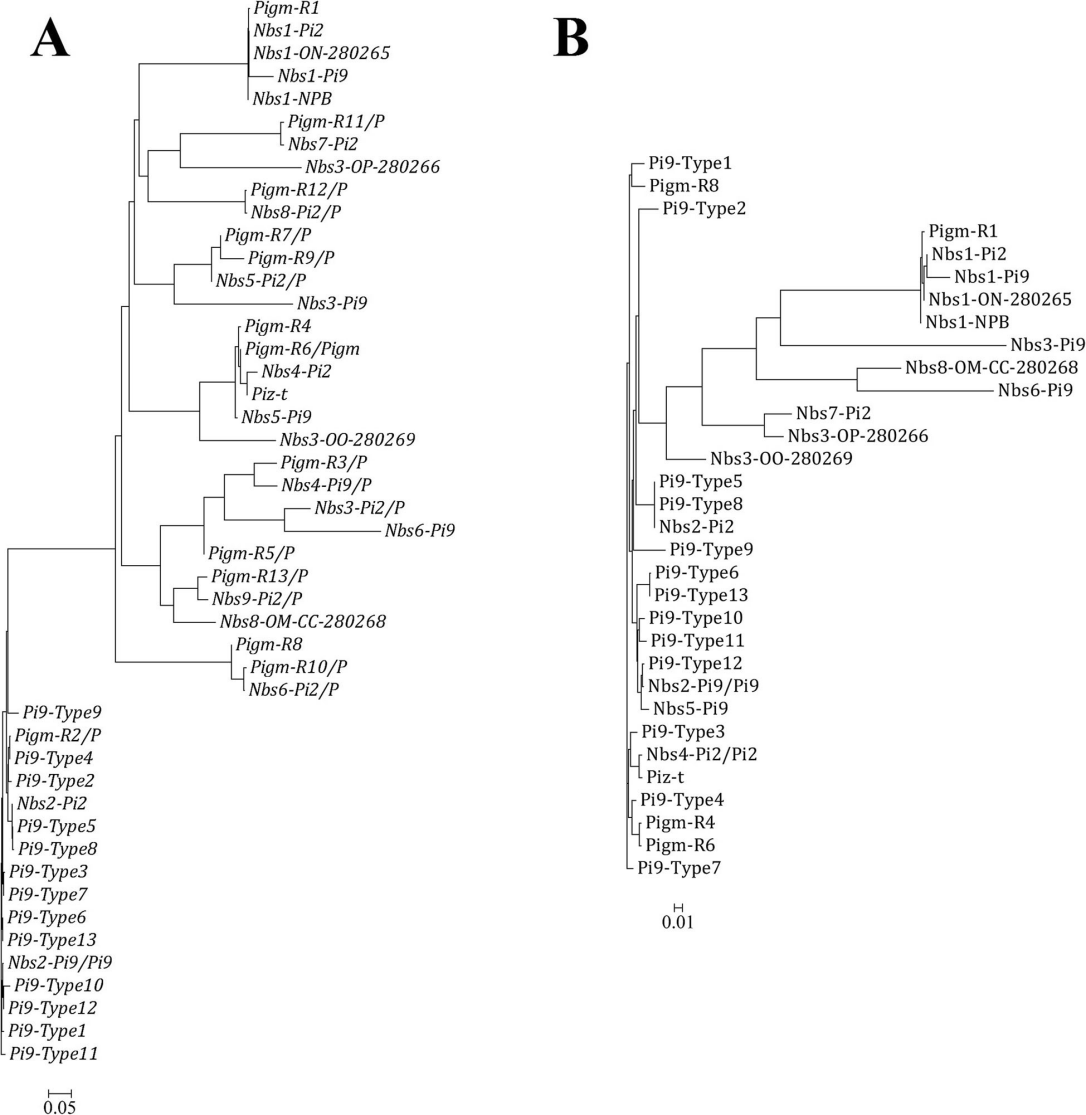
Phylogenetic relationship among gDNA and protein sequences of NBS genes at *Pi2*/*9* locus.Thirteen new *Pi9* allele sequences found in our research materials and the sequences of *Pi9*, *Pi2*, *Piz-t*, *Pigm*, and all NBS disease resistance genes at the tandem repeat region of the *Pi2*/*Pi9* locus were used for analysis. **a**. Phylogenetic tree of the genomic sequences (from ATG to TGA, including the introns) for 13 novel *Pi9* alleles based on our sequencing results and NBS genes at *Pi2*/*9* locus; **b**. Phylogenetic tree of the protein sequences of 13 novel Pi9 alleles protein and NBS proteins at Pi2/9 locus. Bootstrap values (1000 replications) are mentioned at the branch nodes

The phylogeny of *Pi9* alleles was analyzed after adding the sequences of *Pi9*, *Pi2*, *Piz-t*, *Pigm* and all NBS disease resistance genes at the tandem-repeat region of the *Pi2/Pi9* locus. The NBS genes in BAC clones *Pi9* (DQ285630), *Pi2* (DQ352453), *Pigm* (KU904633), *Piz-t* (DQ352040), and others (GQ280265, GQ280266, GQ280267, GQ280268, GQ280269, DQ454158) from the NCBI database were used for the phylogenetic analysis. A phylogenetic tree was constructed using nucleotide sequences that included the complete ORF and 350 bp of the promoter sequence upstream of the start codon or the predicted protein sequences. Phylogenetic analysis of gDNA indicated that the *Pi9* homologues in these varieties were more closely related to each other than to the other homologues (Fig. 3). The results obtained using genomic and protein sequences differed substantially: *Pi9* alleles that encode similar protein sequences did not show higher genomic sequence similarity (Fig. 3a and Fig. 3b). However, *Pi9*-Type6/13 and *Pi9*-Type5/8 showed very high genomic and protein sequence similarity (Fig. 3).

### Evaluation of Blast-Resistance Using Different *M. oryzae* Isolates

About 34 *M. oryzae* isolates from Hubei, Jiangxi, Hunan, Fujian, and Guangdong Provinces, China, were sequenced and analyzed for *AvrPi9* and *AvrPiz-t*. All 34 *M. oryzae* isolates contained *AvrPi9*, and about half of them contained the *AvrPiz-t* gene. We assessed the leaf blast-resistance of the 13 alleles of *Pi9* donors and their *Pigm, Pi2*, *Pi9,* and *Piz-t* donors in the greenhouse using these 34 *M. oryzae* isolates. The donors of *Pigm*, *Pi2*, *Pi9*, and *Piz-t*, i.e., Gumei4, C101A51, 75–1-127, and DY1H, respectively, showed broad-spectrum resistance to rice blast with resistance frequencies ranging from 58.8 to 94.1%. Since the collected *M. oryzae* isolates all contained *AvrPi9* (Table S7), the donors of *Pi9* showed the best broad-spectrum resistance of 94.1%. The resistance frequencies of donors of the *Pi9* allele ranged from 23.5 to 100%. The donor GD-1S (containing the *Pi9*-Type5 allele) and the donor THAVALU (containing the *Pi9*-Type9 allele) were resistant to all 34 blast isolates, with a resistance frequency of 100%, an even higher resistance frequency than GM4H. The donors YD4038 and ZWH210, containing the *Pi9-Type6* and *Pi9-Type10* alleles, were resistant to more than 30 of 34 blast isolates with a resistance frequency of more than 91.2% (Table 4). The blast-resistance evaluation scale was used to evaluate leaf blast from 0 to 9 according to IRRI standard for each blast isolates, and the resistance level was calculated as average with 34 blast isolates infection for each varieties. The resistance level of donors of the *Pi9* allele ranged from 1.79 to 5.62, and there was also a correlation between resistance ratio and resistance level. When the resistance ratio was higher than 85%, the resistance level was usually less than 3. The varieties with a resistance ratio greater than 85% and a resistance value less than 2 were chosen as broad-spectrum resistant varieties to been used to construct the NILs.

**Table 4:** Disease responses of *Pigm*, *Pi2*, *Pizt*, *Pi9* and *Pi9* alleles honor plants to *M. oryzae* isolates

Gene	Varieties	Resistance ratio	Resistance level
None	LTH	0.00%	8.15 ± 0.77
None	J23B	0.00%	6.22 ± 1.19
*Pigm*	GM4H	91.20%	2.43 ± 1.82
*Pi2*	C101 A51	67.60%	3.81 ± 1.84
*Pi9*	75–1-127	94.10%	2.71 ± 1.14
*Pizt*	DY1H	58.80%	4.16 ± 2.70
*Pi9*-Type1	Heo Trang	26.50%	5.10 ± 2.14
*Pi9*-Type2	IR64	23.50%	5.62 ± 1.70
*Pi9*-Type3 ^a^	HC1H	85.30%	2.88 ± 2.09
*Pi9-*Type4 ^a^	PIIB	88.20%	2.01 ± 1.31
*Pi9*-Type5 ^a^	GD-1S	100.00%	1.79 ± 0.72
*Pi9*-Type5-*Pi2*	CT 18664–9–18-1-3-2	70.60%	3.15 ± 2.00
*Pi9*-Type5-*Piz-t*	ASD 18	67.60%	3.56 ± 1.76
*Pi9*-Type6 ^a^	YD4038	91.20%	2.03 ± 1.25
*Pi9*-Type7	KAUKKYI ANI	61.80%	4.04 ± 2.45
*Pi9*-Type8	DY1H	58.80%	4.16 ± 2.70
*Pi9*-Type9 ^a^	THAVALU	100.00%	1.79 ± 0.72
*Pi9*-Type10 ^a^	ZWH210	94.10%	2.07 ± 1.59
*Pi9*-Type11 ^a^	R03138	88.20%	2.75 ± 1.58
*Pi9*-Type12	JP-5	79.40%	2.72 ± 1.64
*Pi9*-Type13	ZD5H	55.90%	4.43 ± 2.36

^a^When Resistance ratio was greater than 85% and Resistance level was less than 2, NILs of allele materials were done

Resistance ratio, the proportion above MR inoculated with 34 *M. oryzae* isolates; Resistance level, the average level inoculated with 34 *M. oryzae* isolates. None, no functional *Pi9* allele; *Pi9-*Type5 has three different type *Pi9*-Type5, *Pi9*-Type5-*Pi2* and *Pi9*-Type5-*Piz-t*. Donors of *Pi9*-*Type5*-*Pi2* and *Pi9*-*Type5*-*Piz-t* carried *Pi2* and *Piz-t* genes, respectively. Donors of *Pi9*-Type4 carried *Pigm*. Donors of *Pi9*-Type8 carried *Piz-t*

### Construction of the NILs and Evaluation of Blast-Resistance in Field Trials

In order to choose the ideal donor for rice blast breeding and characterize the novel *Pi9* alleles function, seven *Pi9* allele genes with a resistance ratio of > 85% (*Pi9*-Type3, *Pi9*-Type4, *Pi9*-Type5, *Pi9*-Type6, *Pi9*-Type9, *Pi9*-Type10, and *Pi9*-Type11) and four cloned genes (*Pigm*, *Pi2*, *Pi9* and *Piz-t*) were introduced into the recurrent parent J23B to construct NILs. For the NILs construction, different donors were crossed to J23B and the F1 hybrids were backcrossed with J23B to obtain the BC1F1 population, then from the BC1F1 population we selected six plants displaying closest phenotypic resemblance to J23B and containing the *Pi2/Pi9* locus with markers selection to profile using our RICE6K array, then we selected one plant with the target *Pi9* allele gene and a background closest to J23B and without any region containing the known rice blast genes, to backcross with J23B up to BC3F1. After three consecutive rounds of selection, the chip data for BC3F1 showed that the background of the NILs was more than 85% similar to J23B, and these newly developed NILs included the whole tandem-repeat region of the *Pi2/Pi9* locus, but no other known resistance tandem-repeat regions, such as the *Pik* locus on Chr. 11 and the *Pi3*/*Pi5* locus on Chr. 9. We examined the resistance of the seven alleles and four cloned genes in both the J23B and donor parents’ background to leaf blast and neck blast at Enshi and Yichang in 2017.

Among the four cloned genes, *Pigm* conferred the greatest resistance to both leaf blast and neck blast in the J23B or donor parent background at both Enshi and Yichang (Table 5). Like the cloned genes, among the seven *Pi9* allele genes, *Pi9*-Type6, *Pi9*-Type10, and *Pi9*-Type11 showed significantly enhanced resistance to leaf blast and neck blast in the J23B background compared with the control J23B at both Enshi and Yichang (Table 5). *Pi9*-Type3, *Pi9*-Type5, and *Pi9*-Type9 significantly enhanced resistance to leaf blast at Enshi (Table 5). *Pi9*-Type3 also significantly enhanced resistance to neck blast at Enshi and Yichang, but *Pi9*-Type5 and *Pi9*-Type9 conferred enhanced resistance to neck blast only in Yichang. All of the *Pi9* alleles conferred enhanced resistance to leaf or neck blast in at least one location. Moreover, *Pi9*-Type6 and *Pi9*-Type11 were associated with greater resistance to both leaf blast and neck blast than the cloned genes *Pi2* and *Piz*-*t* (Table 5).

**Table 5:** Disease responses of *Pigm*, *Pi2*, *Pizt*, *Pi9* and *Pi9* alleles honor plants to *M. oryzae* isolates

Gene	Varieties	Generation	Enshi		Yichang	
			Lr	Nr	Lr	Nr
None	LTH	F0	S	S	HS	S
None	J23B	F0	MS	S	HS	MS
*Pigm*	GM4H	F0	MR	R	HR	HR
*Pi2*	C101 A51	F0	R	MR	MR	MR
*Pi9*	75–1-127	F0	MR	R	HR	R
*Pizt*	DY1H	F0	MR	MR	R	MR
*Pi9*-Type1	Heo Trang	F0	HR	R	HR	R
*Pi9*-Type2	IR64	F0	MS	MS	R	R
*Pi9*-Type3 ^a^	HC1H	F0	R	MR	R	R
*Pi9-*Type4 ^a^	PIIB	F0	R	R	HR	HR
*Pi9*-Type5 ^a^	GD-1S	F0	R	HR	HR	HR
*Pi9*-Type5-*Pi2*	CT 18664–9–18-1-3-2	F0	MR	MR	MR	MR
*Pi9*-Type5-*Pizt*	ASD 18	F0	MR	MS	MR	MR
*Pi9*-Type6 ^a^	YD4038	F0	R	HR	HR	HR
*Pi9*-Type7	KAUKKYI ANI	F0	MR	HR	HR	R
*Pi9*-Type8	DY1H	F0	S	HS	HR	HR
*Pi9*-Type9 ^a^	THAVALU	F0	HR	R	HR	R
*Pi9*-Type10 ^a^	ZWH210	F0	R	HR	HR	MR
*Pi9*-Type11^a^	R03138	F0	HR	HR	HR	HR
*Pi9*-Type12	JP-5	F0	R	HR	HR	MS
*Pi9*-Type13	ZD5H	F0	MR	MR	R	HS
*Pigm*	GM4H	BC3F2	MR	MR	R	HR
*Pi2*	C101 A51	BC2F2	MR	MS	MR	R
*Pi9*	75–1-127	BC3F2	MR	MR	R	R
*Pizt*	DY1H	BC3F2	MR	MS	MR	MR
*Pi9*-Type3	HC1H	BC3F2	MR	MR	MS	R
*Pi9*-Type4	PIIB	BC2F2	MR	R	HR	R
*Pi9*-Type5	GD-1S	BC3F2	MR	MS	R	R
*Pi9*-Type6	YD4038	BC3F2	R	MR	R	R
*Pi9*-Type9	THAVALU	BC3F2	MR	MS	R	MR
*Pi9*-Type10	ZWH210	BC2F2	R	MR	R	MR
*Pi9*-Type11	R03138	BC3F2	R	MR	R	R

*Lr* Leaf blast; *Nr* Ear blast and neck blast; Varieties, Representative donor variety containing the allele

^a^When Resistance ratio was greater than 85% and Resistance level was less than 2, NILs of allele materials were done

None, no functional *Pi9* allele. *Pi9-*Type5 has three different type *Pi9*-Type5-*Pi2, Pi9*-Type5-*Piz-t* and *Pi9*-Type5. Donors of *Pi9*-Type5-*Pi2, Pi9*-Type5-*Piz-t* and *Pi9*-Type5 carried *Pi2, Piz-t* and no genes, respectively. Donors of *Pi9*-Type4 carried *Pigm* gene. Donors of *Pi9*-Type8 carried *Piz-t* gene

## Discussion

### Current Status of Cloning and Characterization of Rice Blast R-Genes

Identifying and cloning novel broad-spectrum blast R-genes is critical for breeding resistant rice varieties and has been a major focus of rice genome research. With the development of molecular marker technology, the construction of a high-density genetic linkage map, and the improvement of related molecular techniques, great progress has been made in cloning rice blast R-genes (Chen et al., 2006; Hayashi and Yoshida, 2009; Hittalmai et al., 2000; Lin et al., 2007; Sharma et al., 2005).

The theory of rice blast-resistance genes was first presented by Sasaki in 1922, initiating nearly 100 years of discovery and utilization of these genes in rice breeding (Sasaki et al., 1922). In 1966, Yamasaki cloned the rice blast R-genes *Pia*, *Pii*, and *Pik* (Yamasaki and Kiyosawa, 1966) from Aichi Asahi, Ishikari Shiroke, and Kanto 51. In 1999, Wang cloned the R-gene *Pib* (Wang et al., 1999) by map-based cloning. With improvements in technology, the cloning and exploitation of rice-blast R genes has seen tremendous progress in the twenty-first century. To date, 119 blast R-genes and more than 400 quantitative trait loci (QTLs) have been mapped, and more than 30 blast-resistance genes have been mapped and cloned (Balliniet et al., 2008; Bryan et al., 2000; Chen et al., 2018a; Chen et al., 2006; Chen et al., 2018b; Fukuoka et al., 2009; Inoue et al., 2017; Hayashi et al., 2010; Ishihara et al., 2014; Li et al., 2017; Lin et al., 2007; Liu et al., 2002; Liu et al., 2007; Zhao et al., 2018).

Most blast R-genes, except for a few genes such as *pi21*, *Pid2*, *Pid3*, and *Ptr*, encode nucleotide-binding site leucine-rich repeat (NBS-LRR) proteins (Chen et al., 2006; Fukuoka et al., 2009; Liu et al., 2007; Xu et al., 2014). Analysis of the cloned blast R-genes revealed that most of the broad-spectrum R-genes are located in tandemly repeated gene clusters on chromosomes 6, 9, 11, and 12. Generally, rice blast genes that have an NBS-LRR structure and occur in tandem repeat regions confer broad-spectrum resistance. The structures of tandem-repeat regions differ greatly among rice varieties, harboring various inversions and deletions. Furthermore, many R-genes with highly similar structures are tandemly duplicated with pseudogenes (Dai et al., 2010).

The short arm of chromosome 6 contains at least 10 blast R-genes (*Pigm*, *Pi2*, *Pi9*, *Piz-t*, *Piz*, *Pi22*, *Pi25*, *Pi26*, *Pi40*, and *Pi42*)), arranged as tandem repeats (Deng et al., 2006). *Pi2*, which was cloned from the variety ‘C101 A51’, encodes a 1032 amino acid NBS-LRR protein (Hittalmani et al., 2000; Zhou et al., 2006). *Pi9* is highly similar to *Pi2* but has a different resistance profile (Xiao et al., 2017). *Piz-t*, derived from Xiushui 209, differs from *Pi2* at eight amino acids (Fig. 1) due to differences at more than 20 bases in the coding regions of the two genes. *Pigm* is an unusual R-gene formed by one copy each of *PigmR* and *PigmS* in series. *PigmR* imparts broad-spectrum disease resistance but reduces yield. *PigmS* does not confer resistance but improves seed setting rate. Here, we focused our analysis on this region of chromosome 6 due to its high density of R genes and identified 13 novel alleles. The tandem-repeat region of the *Pi2/Pi9* locus contains at least four cloned R genes (*Pi2*, *Pi9*, *Piz-t*, and *Pigm*) (Dai et al. 2010; Zhou et al., 2006) and is divided into the *Pi2* locus and *Pi9* locus. The *Pi2* locus includes *Pi2*, *Pigm*, and *Piz-t*, while the *Pi9* locus includes *Pi9* (Xiao et al., 2017). We reasoned that these two loci may be functional sites for blast-resistance. We identified no novel alleles at the *Pi2* locus, which contained only the previously cloned genes *Pi2*, *Pigm*, and *Piz-t.* Therefore, we focused on mining the *Pi9* alleles, as this locus is a functional site that might represent the characteristics of these tandem-repeat regions. *Pi9,* a broad-spectrum rice blast-resistance gene cloned by Qu in 2006 (Qu et al., 2006), has been used in China for many years and confers robust broad-spectrum resistance. Here, we sequenced the *M. oryzae* isolates collected from all over China and observed that almost all of the *M. oryzae* isolates contained the *AvrPi9* gene, which was why donors of *Pi9* showed broad-spectrum resistance. Therefore, isolating *Pi9* alleles was of great significance for mining broad-spectrum rice blast-resistance genes. Pi9 protein, like many disease resistance proteins, has a typical CC-NBS-LRR domain. Since this is also true of most other cloned rice blast-resistance proteins, we predict that that only *Pi9* alleles with complete CC-NBS-LRR domains are likely to confer resistance to rice blast.

The genomic sequences vary among the different *Pi9* alleles, especially in the first intron, and there are many SNPs and large insertions or deletions in these alleles compared to the original *Pi9* gene. However, in the first intron, due to the complex sequence results, it was difficult to amplify a single band with a sequence of about 840 bp. Considering that this region is located in the intron, it likely does not affect the translation of the protein, and since we already had about 9 kb of the sequence of the *Pi9* allele, which could be used to distinguish the *Pi9* allele, we gave up further attempting to sequence the 840 bp fragments. The protein sequences of the Pi9 allele types are conserved, especially in the CC-NBS-LRR domain. None of the 13 Pi9 allele proteins have amino acid insertions/deletions in the CC or NBS domain, and we identified only one amino acid insertion in the LRR domains. In the CC domain, only Pi9-Type9 differs from Pi9, by four amino acids. Pi9-Type10/12/13 and Pi9 proteins have identical NBS domains, and Pi9-Type9 has the most amino acid differences in this domain. The LRR region has more amino acid differences among the different allele types, with Pi9-Type5/8 having the most (~ 27) amino acid differences. Pi9-Type12 had the fewest amino acid differences (only 2).

The CC domain is particularly notable. Among the 13 newly identified Pi9 allele proteins, we detected only 4 amino acid differences between the Pi9-Type9 and Pi9 proteins and no differences in remaining Pi9 allele proteins, suggesting that the CC domain must be highly conserved. However, this region of the *Pi9* gene contains the first intron, which has the greatest number of base mutations among the *Pi9* alleles and a large number of insertions/deletions. It is interesting that this region has so much nucleotide sequence variation in the intron but is highly conserved in terms of amino acid sequence. By comparison, there are more amino acid differences in the LRR domain. The observation that the CC region is the most strictly conserved region of the CC-NBS-LRR domain suggests that it might be the most important region for disease resistance. When an amino acid in this region is changed, it could lead to the loss of disease resistance. The LRR domain might be less important; disease resistance might be maintained as long as a complete LRR domain is present, even if there are some amino acid changes. Because all of the Pi9 allele proteins contain a complete CC-NBS-LRR domain, we predict that they have the potential to confer broad-spectrum resistance to rice blast. Exploring such genotypes will help enrich our gene library for resistance to rice blast. After subsequent functional verification, we hope to use the alleles to improve resistance to rice blast.

### Allelic Variants of the R Gene Associated with Rice-Blast-Resistance

We used rice blast resistant varieties of local cultivated rice, wild rice, and core germplasm resources for resistance gene mining Transferring resistance genes from resistant varieties to cultivated varieties can improve rice-blast-resistance. Transferring individual genes or pyramiding multiple R genes can confer race-specific or broad spectrum resistances (Vasudevan et al., 2015). For example, the *Pi54* allele from *Oryza sativa* cv Tetep conferred broad-spectrum resistance against several rice-blast isolates compared to *Pi54* orthologs from *Oryza sativa* cv Co39 (Thakur et al., 2015), suggesting allelic variants of *Pi54* have functionally distinct capacities. Many studies have focused on mining single-copy rice blast-resistance genes, such as *Pi54*. However, due to the complex structure of the tandem-repeat region, few efforts at mining genes in this region have been successful.

In the current study, we attempted to mine homologous alleles of *Pi9* in the tandem-repeat region from different variants based on the notion that this locus could represent the characteristics of these tandem-repeat regions. Thirteen novel alleles of *Pi9* was identified (including three types of *Pi9*-Type5), significantly extending the known *Pi9* allelic series. We used controlled infections to assess the resistance of rice varieties carrying the novel alleles. The varieties identified as being resistant in the nursery trials showed varied disease responses when infected with the single rice-blast isolate, suggesting that the novel *Pi9* alleles vary in their blast-resistance spectra. Some of the novel alleles have unique SNPs, insertions, or deletions in addition to polymorphic residues that are shared between the different alleles. These variations are important for the durability of *Pi9* against *M. oryzae*. The varied patterns among the rice varieties containing the novel *Pi9* alleles to different rice-blast strains reveal their altered resistance spectra. However, this notion requires functional validation, such as transgenic verification, RNA expression analysis, or the construction of NILs.

Due to the distinct genomic structures (such as inversions and deletions) within the tandem-repeat regions of different rice varieties, it was difficult to clone these new alleles in the tandem repeat region. Furthermore, many R-genes with highly similar structures are tandemly duplicated with pseudogenes, which makes it challenging to distinguish candidate alleles from pseudogenes through a transcriptional assay like qRT-PCR. To clearly elucidate the function of these novel *Pi9-like* alleles and to circumvent the interference of other R genes, we constructed NILs to verify the resistance conferred by novel *Pi9-like* alleles at this locus. Through molecular-marker assisted backcrossing (MAB), we successfully pyramided seven *Pi9* allele resistance genes, *Pi9*-Type3/4/5/6/9/10/11, into the susceptible variant J23B to develop BC3F2 lines. These BC3F2 lines exhibited enhanced resistance to rice blast compared to the controls, suggesting that the broad-spectrum blast-resistance conferred was by these novel alleles was not attributable to other R genes.

### Current Status of Molecular Breeding for Rice-Blast-Resistance

Rice blast, caused by the fungus *M. oryzae*, is one of the most important rice diseases worldwide (Ashkani et al., 2015; Shen et al., 2004). Rice blast has been reported in almost all rice-producing areas of the world (Miah et al., 2013; Zhu et al., 2000). Because the physiological races of rice-blast pathogen are highly variable and change rapidly, any gene conferring resistance to a single race is easily overcome (Wu et al., 2016; Xu et al., 2014). The breakdown of resistance can be avoided by developing rice varieties with a large number of broad-spectrum R genes associated with strong resistance. This is of great importance for breeding disease-resistant rice varieties and preventing rice blast.

Rice blast-resistance genes generally encode proteins containing the CC-NBS-LRR domain, and we established that genes located in tandem-repeat regions of the genome often confer broad-spectrum resistance. Monoclonal rice blast-resistance genes such as *Pi36* and *Pi37* (Liu et al., 2007; Lin et al., 2007) usually do not offer lasting resistance. When these genes were transformed into susceptible receptor material using transgenic or traditional hybrid technology, the resistance of these plants was rapidly overcome by pathogenic microorganisms, resulting in loss of resistance (Zhu et al., 2000). The rice blast-resistance genes located in tandem repeat regions often have relatively robust broad-spectrum characteristics and can confer rice-blast-resistance in many regions for a long period of time (Mi et al., 2018; Zhou et al., 2018).

The arrangement of R genes in tandem-repeat regions, which often encode proteins with multiple CC-NBS-LRR domains with similar structures and functions (Dai et al. 2010), may help alleviate the loss of disease resistance caused by mutations of pathogenic microorganisms. However, this genetic structure makes it challenging to clone individual R genes. The *Pi9* locus contains a dozen or so genes with similar structures that are connected in series to form a region of several hundred kilobases. These tandemly repeated genes can have as much as 99% identity with each other (Dai et al. 2010), making it challenging to amplify and sequence a specific gene. Furthermore, the position of the *Pi9* locus often differs significantly from its position in known reference genomes (9311, Nipponbare, MH63, ZS97, and other varieties) (Zhang et al., 2016), adding to the complexity of cloning these genes. After many attempts, our research group has now developed a method to stably amplify *Pi9* allele genes from the tandem-repeat regions.

Using these techniques in this work, we screened 107 rice blast-resistant varieties from 1883 rice varieties and identified 13 novel *Pi9* alleles (including three types of *Pi9*-Type5 alleles). Theoretically, different alleles of *Pi9* will correspond to different tandem-repeat regions in the *Pi9* locus. Since *Pigm*, *Pi2*, and *Piz-t* have been cloned, they are also located in the tandem repeats of this locus. To test the possibility that *Pi9* alleles correspond to different tandem-repeat regions in the *Pi2/Pi9* locus, we sequenced the *Pigm*, *Pi2*, and *Piz-t* genes from the donor materials of the 13 newly identified *Pi9* alleles. *Pi9*-Type4 contained *Pigm* in the tandem repeat region, and *Pi9*-Type8 contained *Piz-t* in the tandem repeat region. Interestingly, *Pi9*-Type5 was located in three different tandem-repeat regions, including one containing *Pi2,* one containing *Piz-t* and the other not containing any cloned gene. These results demonstrate that the structure of the *Pi2/Pi9* locus is particularly complex, but homologue of the *Pi9* allele are conservative and exist in many tandem repeat regions.

Our research group is currently developing chips that could be used to screen rice varieties for differences in the tandem repeat sequence flanking the *Pi9* locus. This can further distinguish different tandem repeats in this region. It is challenging to clone a specific broad-spectrum rice blast-resistance gene in a tandem repeat. However, we used tandem-repeat sequencing results to develop a chip and combine the sequencing information from a relatively conserved genotype of the tandem-repeat region to distinguish among different tandem-repeat regions. Subsequently, the disease resistance of different tandem-repeat regions will be verified, and molecular-marker selection technology will be used to transform the tandem-repeat region with broad spectrum resistance into the susceptible receptor varieties. We therefore aim to confer broad-spectrum resistance to susceptible rice varieties using this technique.

## Conclusions

Thirteen novel *Pi9* alleles were identified from 107 blast resistance varieties through sequence-base allele mining. The developed NILs containing 5 novel *Pi9* alleles fragments (*Pi9*-Type3, 4, 5, 6, 9, and 10) showed broad-spectrum rice blast resistance in the field indicating its potentiality in rice blast resistance genetic improvement. Further studies are required to clone and characterize the functional alleles for the blast resistance.

## Supplementary Information

**Additional file 1: Supplementary Table 1.** Primers for sequence analysis of *Pi9, Pi2, Pigm* and *Pizt* allele genes in rice.Among them, the alleles of *Pi2*, *Pigm* and *Piz-t* share a set of primers for amplification and sequencing; the alleles of *Pi9* use a unique set of primers for amplification and sequencing. The primers used for PCR amplification and sequencing were clearly labelled and listed in the table

**Additional file 2: Supplementary Table 2.** The SSR primers used in molecular-marker assisted backcrossing (MAB). FAM and HEX are different fluorescent label.

**Additional file 3: Supplementary Table 3.** List of 107 rice varieties used for allele mining. Approximately 1883 varieties were grown at trial nurseries of Enshi and Yichang, Hubei Province. We identified 361 varieties from nurseries at Enshi or Yichang that displayed HR or R resistance phenotypes. A PCR-based screen for the presence of *Pi2*, *Pi9*, *Pigm*, or *Piz-t* identified 107 varieties as candidates for allele mining. The sequencing results of all 107 materials are shown in this table

**Additional file 4: Supplementary Table 4.** Sequence of *Pi9* allele genes. The gap region in intron1 replaced with the corresponding sequence of reference *Pi9* sequence was shown in gray.

**Additional file 5: Supplementary Table 5.** Sequence of Pi9 allele proteins

**Additional file 6: Supplementary Table 6.** Primers for sequence analysis of *AvrPi9* and *AvrPiz-t* genes in ***M. oryzae*.**The *AvrPi9* and *AvrPiz-t* genes each use a unique set of primers for amplification and sequencing. The primers used for PCR amplification and sequencing were labelled and listed in the table.

**Additional file 7: Supplementary Table 7.** List the presence of *AvrPi9* and *AvrPiz-t* genes in 34 *M. oryzae.*+, the gene is present in the *M. oryzae*; −, the gene does not exist in the *M. oryzae.*

## Data Availability

The data sets supporting the results of this article are included within the article and its supporting files.

## Abbreviations

QTLs: Quantitative trait loci
NILs: Near-isogenic lines
R gene: Resistance gene
CC: Coiled-coil domain
NBS: Nucleotide-binding site
LRR: Leucine-rich repeats
HR: High resistance
R: Resistance
MR: Middle resistance
MS: Middle ensitivity
S: Sensitivity
HS: High sensitivity
BAC: Bacterial artificial chromosome
SNPs: Single nucleotide polymorphisms
InDels: Insertions and deletions
CDD: Conserved domains database
ORF: Open reading frame
qPCR: Quantitative polymerase chain reaction
MAB: Molecular-marker assisted backcrossing
