# Supplementary material for: Identification of Novel Alleles of the Rice Blast-Resistance Gene Pi9 through Sequence-Based Allele Mining
Source: Rice (N Y). 2020 Dec 7;13:80. doi: 10.1186/s12284-020-00442-z (PMC7721961; doi:10.1186/s12284-020-00442-z)
Supplement: Supplementary file 4 — Additional file 4: Supplementary Table 4. Sequence of Pi9 allele genes. The gap region in intron1 replaced with the corresponding sequence of reference Pi9 sequence was shown in gray. [file 12284_2020_442_MOESM4_ESM.docx]

>Pi9

GCTTGCATGACGTTATGTAGATAGAGATGGCGAATATAATGCGCTGGAAAGTCCCAAGTGAGGATGCAAAACATCTTATAGTGGGTAGTGGAGCCCTGCAAGGACCCTAATATCTAAAGCGCACCTAAACTGATATGGACATCCATTATAGTTAAAGTTAGGGGGAATATGATTCTCTTCCTGTGCACCTAAACCGTAATATGCAGTGAAACGAACGCTATGATACGATGATAAGCTTAATTCCTCTCTCTGCTCAGACTGTTCAGTGCAAAAGCTACCAACGAGAGCTTGTCTCCTTGTGCGGTCGTGAGCTTGCTTGTGCTAAGCTTGAAGGGAGAGTCGAACGAATCCATGGCGGAGACGGTGCTGAGCATGGCGAGGTCGCTGGTGGGCAGTGCCATCAGCAAGGCCGCCTCTGCCGCTGCCAATGAGACGAGCCTCCTGCTCGGCGTCGAGAAGGACATCTGGTACGTACTGCACTGCTCTCGTTTATCCTAGCAAGTTCTTAGGCTCTTAATCTCGAAATTGAGGAACACCATGAAACACTAAAAGAGAGCTCGAAGACTAGGAAAGAAAACTAGAAGACTAAGCTTTGAAAGTCTTCTAAATCCAAGCATCTCGACATTGATCATCCTTGTGCAACATCATCCCTTCCTATTGCTTCACCAGAATCGGTGTCCCTTGTGGAGATCTCTGTCGTAGCGTCAAGGGGAGAATCCGAGAAGCAGAACTAGTCCGCGCTGCCTTCGCTACGCCATCTCCGCCATAGAGGATCTCATCCACGAAACATCCACCATCCAAACGGGAAACTGTTTTAAACACTCGGGTGGATATTCACCCGTTTCTTGCATGTCATCTAAATGGTTATGAAAAATTTTCAAAAAAAAAACATGATAGGTTAATATATAATATATCATCTCACAAATATGCAAGTTCAAATTCAACTTTTATAAGTTGTAAGTATAACAGGACGTTCATCTCACAAATATGCAAGTTTAAATTTAACTTTTACAAGTTGTAAGTGTAACAGTACGTCCATCGGATAGATTAATATCCATCTCCCCATCCAAACCCGTTGTTGCACCATCTGTCGAATCCGGCTGTGGACGCTCGGAGGCAAGAGCTAGCTCACCCGTCCCACACACACACCCAACGACGTCACAAGCGCCTCCGAACAACGCCAACTGATAACTTGGCAGCTCCTACGTGCCGACGTCGCGGTACTTGCCGGCGCTCCTAGCGCACGCACCGTCGAACCACACCGTCACCGACCAACTACCCACCGCCGCCGACTTCTGCCTCATCTGCCATCGTCGCCCTAGCCCAAGTTATCATCGTGGCAATTGCCGAGGCTCCTAAGTGTGCCACGGCCGAGGCAAAGTTCTAACTGAATCAGAGTATCAGACAGCCACCACCGACGCTACTTCTGCTTCATCTGCCATCGCCGTACTAGTTCAAGTTGTCGCTGTGGCAATCATGGGCCCTCCTAGCGTGCCACACAACCGGACAGCCACGACATCCCCCATCACTGTTGTTATTGCCGCGCCCTGACCCCTATCGTCGTCGCTCTTAGCGCGTCGTCGAGCCGACCAGCCACTGTCGTGCAGATGAAAAAAAAACACATATTGGCCTGAGAGATCTGCTTAGTTCCAGTGCAGGTCCAACATGCTGTGAGATGCGGGCGTGCCAGTCAGTTTGATCTTGCAACTGACAAGATATATAAATAGCAGATAAAACAGCCTATCGACTAACAAGCCGATGGAGTAATTCCAGCCGATAGCCGATATTAGCCGATGCCGATTCTAGCCGATGTCGATAGGGTTTTGAACTATCGGCTATATGTCCAATGTAGGTAATGATATAAAGACAATTGGCTGATGATAATAAAATATAAAAATATAATCCAATAGAAACCAATCGGCTAATAATAAGTATTGATCCGATAGTTAAAGCATACATCGGCTAAAAGTCCGATGTCATAAAATCCAATCGATTTAGATAAACAGTGAAACCTTTGTTGCAATCGGCTAAATCCAACTTGTATGTAATCTTCGTAAGCCGATGAACGTCCAGATAACTTATCGGCTAGCACCTCGATAAAACACTAGCATGAACCTATCGGCTTAACAAGATTTATATTATCAACAACAATCTAGTAGGTCGGACCTAACCGATGCAACACGTATTAGATATGATAATCTAATACTTGATGAGCCAATAAATCTGTCTAATGTGATGGATATAACAAATCTATTTATAAAAGCATTGCGATTGTAGAGATATATCGGCTAAGACAGAATATCAGACCTAACTAAACCGATGCGTCTCTAAACACAATGCAATTAATTAGAGATATAATTGAGATATCAGCTAGGCAAATATATCAATCAAACTAGAGCGATCCAAGAGATCGGAGCAATGCAGCCTTGAACAACACCAATGTAGCCGATGGATTCACCAGGGCCGACGGAACGTAGGACTTACCCCTTCCCTGAAGATCGGGCTGAACCAATGCAGTCCCACGTCAGGTGCCAAATTCCGCCGGTTGATAAGTAAAACCTTAGAAAAGAGGATGACGATGCGCCGAGAGTAGTATTGATCGAGAGATAAATTGCAATGACCCTGGATGTACATATTTGTACCCATGGGTAGATATTAGTTCTTGTAGGACAAGAAAGAAACTTTCCTAAAGATAAAATGAAAACATAAAGTCTTTATTGGATACTAAACACACTTTCCTAAAGATAAAAGGAAACTAAACCCTGCCTAATTAATAGATAAACTGCCATGTCGTATCCTCCTTGAACTCGGACTCTTTTAGATAAGCTTCCTTTAACTAATCTTTACCCGAATCCATCAAGAATACAAATGTTGGCATTGATAGTTTTCATCGGTCAATTATAGGACTTTGAAGCCGATACTGACTCTAAGCCGATGACTACTTTGGGCTTACCAAATTTTGTTGTTAATATGTCGCGACCACCATCACCGGCCAGCCACCCTGATCATTGTTGTTGACTCAGCATTCGCCAGGCTGAGCAGTCCACATACATGCCGCCATCTCCATGGCAGTGTCGTTGCCGCCCCTTTCTCCTAGAGCCGCCGCAGCGCTCTTCGACACACCTACTGCATCGTCGAGCAGTCGTGCTACCACCTCCTCCATCGACCATAGCCGCCTCTTCTGCTGCACCGGATCCACCCACACCAACCACCAGATACAGTCAAGCCCTCATTCCTGGATCCCATATCCATCCATGCCACTACTGTGCTGCCCAGTCCAAGGAATGGAGCGAAGGAGGAAGCCCCGCCGCTGCCCTCCCGGCGGCCACATGCACTCCAGTGCCTTGCTCCGACGGCAGCGAGGTTGGAAAATGGGTGGCAGCGGCTAGGGTTTATCTGGGGAGAAGGAAAAGGAGAGGGAGGGGGGGGGAGGGTCCACTTCCAGCTTAATTAGCCTAGATCTTATTGACAAATCAGTTGCTGGGTGCACAAACATGTTATTTTTTTTGCATGACCAATCTTGAACACTTAGGTATGTTAGTTGAGTGGACACTGGTCTATCTGAAACATCTCTTCACATGGAGGCTGCGAATGAGTTTTCTTTTTGAGAGACCAAAGTTTCGTTGTATGTTAAGTGATAAAGCCTTGGTAAGAAATGCTACCACAAACGAACTAATAACTCCAAACGTAAAGTGGAGGAACCCGTATGGGTGACTCGAGTGGCGACAAACTCTAGCACCTCCACCTCCTTGGACGGGCTGCGGCGGTGCTTTCGGCATCCCAGTCTTCTTGGAGGCATCATCTAGAATTAAGGTCTTGTTATTGCTTAGCATGCCTTAGGGCACGTCCAGTGTTTAGTTCGACTAAAACTTCCATGAAAGCCAAACAAAAGTTCTGTTTGACCACCACAGTGTAAAAATCGATTGTGGGACCCATGCAAAAAAATCACAATCTCAGCTGCCTATGCTCTCCTCCTGGACCTGATAGCCGTGCACAACAAATATTTTTTTAAACTGGATGTGTTCGGCTTCTCTTTAAAGATCGTTTTTTCCTCTGACACTTACCAACCGGCTTTCACAGTGTGGTCAGTTCTTTTTTTTTTTACGCAAAGTTTGATTTTAGTCAGACACGGGAGGATCTGTTAAGCAGGCTTGGAAATTTCGGACCCCTCCAATACAATATTATTTTAGCCAAAATTTCTAATTTTTTAATTTTTCATGAATTTTGGTAATATTTGTTCTAATTTAACTAAATTTTGTTCAAAATTTCGGTCTATCAGTGACCTCCGATCAAATCAGTTAAACCGAGAAAATAAACCATGCTCTTAAGAGAGTTTGGTATGGTTCAATATCAAAACTTATAGTCTTGCAATTTTTTCTACCCTTTATCTTTTTCCCTGACTATTTAGTATGGATCGTTTAAAAAAAAGAAAGCCCATTGGTGACCAAGGGCTTGTTTGATTCAAGACCATCCCTAGCCTTACCAACCTTTTGGCAATGGCAAAAATTGGTTGTTGCCAAAAATATTGGCACAAATTGGCTAAGCCTATGATTGGTTTCTACCAAAGTTGAATTTTGGCATTCAATCAAGCCAAATAATTTGGCAATAACATTTTCTTATCTATGGATATAACATATGGCAAATATTTTGGCATTACCATTTTCTTTTTGCCAAACATGTTATTCCTTTTGAATGACCAATCTTGACACCTTATGTATGTTAGTAGTGGAATCGACACTATTCTATCTAAAACATCTCTTTACATAGAGGCCGCTAATAATTTTTCTTTGAGATAACCAAATTTTCCTTACAAGTTAAGCAACAAAGCCCATTGGTAAGATATGCTACGACAAATGAACTAATAACTCCAAACGTAAAGCGGAGGATCCCGCATTTCCCACGTGGGTGACTCGAGCGGTGACAAACCCTAGTACCTCCACCCCCTTGGGTGGGTTGTGGTGGCACTTTCGGCACCGTATTTTCCTTGGACGGATCATTTAGAAAGTCCTATTATTGCCTAGTATGCCTTGACAGTTTAGGCAACACTCTTGGATGGTGGTGTCCTTTGCCCTGGTGATCTAGTAGCCCATGGATGTTTAGTTATTTGGACATGGTGTTGGATGGTGCGCTCGTGGGCCTGTTGTAGGTCTGGTGCCAACCAGTCATGCTTAGAAATAGCCGGATAGGTGCACAGTGCTAGTTCTTTACTTGGTGGTTTGTGCAGCGCTATCGACATGTGGTGGTGTGCTTTTTCTTTGTCCGGATAATAATCTCATAGGGCTATACTCTTGTTATTTTGCTGCTATATTATTATGATAACTTGGTATGGTTCGTTTTTTCTTTTTTTGGAAAAACACCTAGTTGATCAAGGGCTTGTTTGGTTCAAGTGCATTCCTAATCTTACCTTTTCTTTTTTTTTTCAATGGCAAGAATTGTTCATTGCAAAAAAAAAAAGAGATAAAAATTGGCTAGGCTTACGTTTTGGTTCTTACCAAAGTTGTACTTTGAGACCAAATATATGGCAAAATTTTGGCATAACCTTTTTTTTTTTTGCTTGGTTGAGCTTGGTACAAACCAATCAGTCACAAAATAGACTGTCATGAATCACGCCTACTAAATTCCTTTGAACCGAACTAGAATATATTTGCTCTTAAAAGATTTCTTGATTTCAATTGGTACCATTTACTAGTAGAAACTTAAATTTAAATTTTAAAAACAAAATCATAATATTGTTGTTATGGAAATTTTAGTCATTTTAGTACTTTTGTAATATATGAGTTGGGTTATACTTGAGATATCCTAAATTGCTTTAAGATGAACAATTGCTAGGTATATCAAAGATGAGCTAAAAACAATGCAGGCATTCCTTAGAGCTGCTGAAGTTATGAAAAAGAAAGATGAACTATTAAAGGTTTGGGCAGAGCAAATACGTGACCTGTCGTATGACATTGAAGATTCCCTTGATGAATTTAAAGTCCATATTGAAAGCCAAACCCTATTTCGTCAGTTGGTGAAACTTAGAGAGCGCCACCGGATCGCTATCCGTATCCACAACCTCAAATCAAGAGTTGAAGAAGTGAGTAGCAGGAACACACGCTACAATTTAGTCGAGCCTATTTCCTCCGGCACAGAGGATGACATGGATTCCTATGCAGAAGACATTCGCAATCAATCAGCTCGAAATGTGGATGAAGCTGAGCTTGTTGGGTTTTCTGACTCCAAGAAAAGGCTGCTTGAAATGATCGATACCAATGCTAATGATGGTCCGGCCAAGGTAATCTGTGTTGTTGGGATGGGTGGTTTAGGCAAGACAGCTCTTTCGAGGAAGATCTTTGAAAGCGAAGAAGACATTAGGAAGAACTTCCCTTGCATTGCTTGGATTACAGTGTCACAATCATTTCACAGGATTGAGCTACTTAAAGATATGATACGCCAACTTCTTGGCCCCAGTTCTCTGGATCAACTCTTGCAAGAATTGCAAGGGAAGGTGGTGGTGCAAGTACATCATCTTTCTGAGTACCTGATAGAAGAGCTCAAGGAGAAGAGGTACTTTGTTATTCTAGATGATCTATGGATTTTACATGATTGGAATTGGATAAATGAAATTGCATTTCCTAAGAACAATAAGAAGGGCAGTCGAATAGTAATAACCACTCGGAATGTTGATCTAGCGGAGAAGTGTGCCACAGCCTCACTGGTGTACCACCTTGATTTCTTGCAGATGAACGATGCCATAACATTGCTACTGAGAAAAACAAATAAAAATCATGAAGACATGGAATCAAATAAAAATATGCAAAAGATGGTTGAACGAATTGTAAATAAATGTGGTCGTCTACCATTAGCAATACTTACAATAGGAGCTGTGCTTGCAACTAAACATGTGTCAGAATGGGAGAAATTCTATGAACAACTTCCTTCAGAACTAGAAATAAACCCAAGCCTGGAAGCTTTGAGGAGAATGGTGACCCTAGGTTACAACCACCTACCATCCCATCTGAAACCATGCTTTTTGTATCTAAGTATCTTTCCTGAGGATTTTGAAATCAAAAGGAATCGTCTAGTAGGTAGATGGATAGCAGAAGGGTTTGTTAGACCAAAGGTTGGGATGACGACTAAGGATGTCGGAGAAAGTTACTTTAATGAGCTAATCAACCGAAGTATGATTCAACGATCAAGAGTGGGCATAGCAGGAAAAATTAAGACTTGTCGAATCCATGATATCATCCGTGATATCACAGTTTCAATCTCGAGACAGGAAAATTTTGTATTATTACCAATGGGAGATGGCTCTGATTTAGTTCAGGAAAACACTCGCCACATAGCATTCCATGGGAGTATGTCCTGCAAAACAGGATTGGATTGGAGCATTATTCGATCATTAGCTATTTTTGGTGACAGACCCAAGAGTCTAGCACATGCAGTTTGTCTAGATCAATTGAGGATGTTACGGGTCTTGGATCTTGAAGATGTGACATTCTTAATCACTCAAAAAGATTTCGACCGTATTGCATTGTTGTGCCACTTGAAATACTTGAGTATTGGATATTCGTCATCCATATATTCACTTCCCAGATCCATTGGTAAACTACAGGGCCTACAAACTTTGAACATGCTGAGAACATACATTGCAGCACTACCAAGTGAGATCAGTAAACTCCAATGTCTGCATACTCTTCGTTGTAGTAGAAAGTTTGTTTATGACAACTTTAGTCTAAACCACCCAATGAAGTGCATAACTAACACAATATGCCTGCCTAAAGTATTCACACCTTTAGTTAGTCGCGATGATCGTGCAAAACAAATTGCTGAATTGCACATGGCCACCAAAAGTTGCTGGTCTGAATCATTCGGTGTGAAGGTACCCAAAGGAATAGGTAAGTTGCGAGACTTGCAGGTTCTAGAGTATGTAGATATCAGGCGGACCAGTAGTAGAGCAATCAAAGAGCTGGGGCACTTAAGCAAGTTGAGGAAATTAGGTGTGATAACAAAAGGCTCGACAAAGGAAAAATGTAAGATACTTTATGCAGCCATTGAGAAGCTCTCTTCCCTCCAATCTCTCTATGTGAATGCTGCGTTATTATCAGATATTGAAACACTTGAGTGCCTAGATTCTATTTCATCTCCTCCTCCCCTACTGAGGACACTCGGGTTGAATGGAAGTCTTGAAGAGATGCCTAACTGGATTGAGCAGCTCACTCACCTGAAGAAGATCTACTTATTGAGGAGCAAACTAAAGGAAGGTAAAACCATGCTGATACTTGGGGCATTGCCCAACCTCATGGTCCTTTATCTTTATTGGAATGCTTACCTTGGGGAGAAGCTAGTATTCAAAACGGGAGCATTCCCAAATCTTAGAACACTTCGTATTTACGAATTGGATCAGCTAAGAGAGATGAGATTTGAGGATGGCAGCTCACCCCTGTTGGAAAAGATAGAAATCTCTTGCTGCAGGTTGGAATCAGGGATTATTGGTATCATTCACCTTCCAAGGCTCAAGGAGATTTCACTTGAATACAAAAGTAAAGTGGCTAGGCTTGGTCAGCTGGAGGGAGAAGTGAACACACACCCAAATCGCCCCGTGCTGCGAATGGACAGTGACCGAAGGGATCACGACCTGGGGGCTGAAGCCGAAGGATCTTCTATAGAAGTGCAAACAGCAGATCCTGTTCCTGATGCCGAAGGATCAGTCACTGTAGCAGTGGAAGCAACGGATCCCCTTCCCGAGCAGGAGGGAGAGAGCTCGCAGTCGCAGGTGATCACGTTGACGACGAACGATAGGTCAGTCACTCCCTACATGGCAGCTTAATTAACTTGTTTCTAATTCTCTTCTTGTTCAGTATTAGCCATCAGGTGAGGGCGATGATTTCAACTCACTTTTCATCTCTCTCGTTTTCTTAACCTGACA

>Pi9-Type1

GCTTGCATATGACGTCATGTAGATAGAGATGGCCAATATAATGCGCTGGAAAGTCCAAAGTGAGGATGCAAAACATCTTATAGTGGGTAGTGGAGCCATGCAAGGACCTGGTCTAAAGCGCACCTAAACCGTAATGTGGACTGCCATTATAGTTAAAGTTAGGGGGAATATGATTCTCTTCATGTGCACCTAAACCGTAATATGCAGTGAAACGAACGCTATGATATGATGATAAGCTTAATTCCTCTCTCTGCTCAGACTGTTCAGTGCAAAAGCTACCAACGAGCTTGTCTCCTTGTGCGGTCGTGAGCTTGCTTGTGCTAAGCTTGAAGGGAGAGTCGAACGAATCCATGGCGGAGACGGTGCTGAGCATGGCGAGGTCGCTGGTGGGCAGTGCCATCAGCAAGGCCGCCTCTGCCGCTGCCAATGAGACGAGCCTCCTGCTCGGCGTCGAGAAGGACATCTGGTACGTACTGCACTGCTCTCGTTTATCCTAGCAAGTTCTTAGGCTCTTAATCTCGAAATTGAGGAACACCATGAAACACTAAAAGAGAGCTCGAAGACTAGGAAAGAAAACTAGAAGACTAAGCTTTGAAAGTCTTCTAAATCCAAGCATCTCGACATTGATCATCCTTGTGCAACATCATCCCTTCCTATTGCTTCACCAGAATCGGTGTCCCTTGTGGAGATCTCTGTCGTAGCGTCAAGGGGAGAATCCGAGAAGCAGAACTAGTCCGCGCTGCCTTCGCTACGCCATCTCCGCCATAGAGGATCTCATCCACGAAACATCCACCATCCAAACGGGAAACTGTTTTAAACACTCGGGTGGATATTCACCCGTTTCTTGCATGTCATCTAAATGGTTATGAAAAATTTTCAAAAAAAAAACATGATAGGTTAATATATAATATATCATCTCACAAATATGCAAGTTCAAATTCAACTTTTATAAGTTGTAAGTATAACAGGACGTTCATCTCACAAATATGCAAGTTTAAATTTAACTTTTACAAGTTGTAAGTGTAACAGTACGTCCATCGGATAGATTAATATCCATCTCCCCATCCAAACCCGTTGTTGCACCATCTGTCGAATCCGGCTGTGGACGCTCGGAGGCAAGAGCTAGCTCACCCGTCCCACACACACACCCAACGACGTCACAAGCGCCTCCGAACAACGCCAACTGATAACTTGGCAGCTCCTACGTGCCGACGTCGCGGTACTTGCCGGCGCTCCTAGCGCACGCACCGTCGAACCACACCGTCACCGACCAACTACCCACCGCCGCCGACTTCTGCCTCATCTGCCATCGTCGCCCTAGCCCAAGTTATCATCGTGGCAATTGCCGAGGCTCCTAAGTGTGCCACGGCCGAGGCAAAGTTCTAACTGAATCAGAGTATCAGACAGCCACCACCGACGCTACTTCTGCTTCATCTGCCATCGCCGTACTAGTTCAAGTTGTCGCTGTGGCAATCATGGGCCCTCCTAGCGTGCCACACAACCGGACAGCCACGACATCCCCCATCACTGTTGTTATTGCCGCGCCCTGACCCCTATCGTCGTCGCTCTTAGCGCGTCGTCGAGCCGACCAGCCACTGTCGTGCAGATGAAAAAAAAACACATATTGGCCTGAGAGATCTGCTTAGTTCCAGTGCAGGTCCAACATGCTGTGAGATGCGGGCGTGCCAGTCAGTTTGATCTTGCAACTGACAAGATATATAAATAGCAGATAAAACAGCCTATCGACTAACAAGCCGATGGAGTAATTCCAGCCGATAGCCGATATTAGCCGATGCCGATTCTAGCCGATGTCGATAGGGTTTTGAACTATCGGCTATATGTCCAATGTAGGCAATGATATAAAGACAATTGGCTGATGATAATAAAATATAAAAATATAATCCAATAGAAACCAATCGGCTAATAATAAGTATTGATCCGATAGTTAAAGCATACATCGGCTAAAAGTCCGATGTCATAAAATCCAATCGATTTAGATAAACAGTGAAACCTTTGTTGCAATCGGCTAAATCCAACTTGTATGTAATCTTCGTAAGCCGATGAACGTCCAGATAACTTATCGGCTAGCACCTCGATAAAACACTAGCATGAACCTATCGGCTTAACAAGATTTATATTATCAACAACAATCTAGTAGGTCGGACCTAACCGATGCAACACGTATTAGATATGATAATCTAATACTTGATGAGCCAATAAATCTGTCTAATGTGATGGATATAACAAATCTATTTATAAAAGCATTGCGATTGTAGAGATATATCGGCTAAGACAGAATATCAGACCTAACTAAACCGATGCGTCTCTAAACACAATGCAATTAATTAGAGATATAATTGAGATATCAGCTAGGCAAATATATCAATCAAACTAGAGCGATCCAAGAGATCGGAGCAATGCAGCCTTGAACAACACCAATGTAGCCGATGGATTCACCAGGGCCGACGGAACGTAGGACTTACCCCTTCCCTGAAGATCGGGCTGAACCAATGCAGTCCCACGTCAGGTGCCAAATTCCGCCGGTTGATAAGTAAAACCTTAGAAAAGAGGATGACGATGCGCCGAGAGTAGTATTGATCGAGAGATAAATTGCAATGACCCTGGATGTACATATTTGTACCCATGGGTAGATATTAGTTCTTGTAGGACAAGAAAGAAACTTTCCTAAAGATAAAATGAAAACATAAAGTCTTTATTGGATACTAAACACACTTTCCTAAAGATAAAAGGAAACTAAACCCTGCCTAATTAATAGATAAACTGCCATGTCGTATCCTCCTTGAACTCGGACTCTTTTAGATAAGCTTCCTTTAACTAATCTTTACCCGAATCCATCAAGAATACAAATGTTGGCATTGATAGTTTTCATCGGTCAATTCTAGGACTTTGAAGCCGATACTGACTCTAAGCCGATGACTACTTTGGGCTTACCAAATTTTGTTGTTAATATGTCGCGACCACCATCACCGGCCAGCCACCCTGATCATTGTTGTTGACTCAGCATTCGCCAGGCTGAGCAGTCCACATACATGCCGCCATCTCCATGGCAGTGTCGTTGCCGCCCCTTTCTCCTAGAGCCGCCGCAGCGCTCTTCGACACACCTACTGCATCGTCGAGCAGTCGTGCTACCACCTCCTCCATCGACCATAGCCGCCTCTTCTGCTGCACCGGATCCACCCACACCAACCACCAGATACAGTCAAGCCCTCATTCCTGGATCCCATATCCATCCATGCCACTACTGTGCTGCCCAGTCCAAGGAATGGAGCGAAGGAGGAAGCCCCGCCGCTGCCCTCCCGGCGGCCACATGCACTCCAGTGCCTTGCTCCGACGGCAGCGAGGTTGGAAAATGGGTGGCAGCGGCTAGGGTTTATCTGGGGAGAAGGAAAAGGAGAGGGAGGGGGGGGGGGGGGGGGAGGGTCCACTTCCAGCTTAATTAGCCTAGATCTTATTGACAAATCAGTTGCTGGGTGCACAAACATGTTATTTTTTTTGCATGACCAATCTTGAACACTTAGGTATGTTAGTTGAGTGGACACTGGTCTATCTGAAACATCTCTTCACATGGAGGCTGCGAATGAGTTTTCTTTTTGAGAGACCAAAGTTTCGTTGTATGTTAAGTGATAAAGCCTTGGTAAGAAATGCTACCACAAACGAACTAATAACTCCAAACGTAAAGTGGAGGAACCCGTATGGGTGACTCGAGTGGCGACAAACTCTAGCACCTCCACCTCCTTGGACGGGCTGCGGCGGTGCTTTCGGCATCCCAGTCTTCTTGGAGGCATCATCTAGAATTAAGGTCTTGTTATTGCTTAGCATGCCTTAGGGCACGTCCAGTGTTTAGTTCGACTAAAACTTCCATGAAAGCCAAACAAAAGTTCTGTTTGACCACCACAGTGTAAAAATCGATTGTGGGACCCATGCAAAAAAATCACAATCTCAGCTGCCTATGCTCTCCTCCTGGACCTGATAGCCGTGCACAACAAATATTTTTTTAAACTGGATGTGTTCGGCTTCTCTTTAAAGATCGTTTTTTCCTCTGACACTTACCAACCGGCTTTCACAGTGTGGTCAGTTCTTTTTTTTTTTACGCAAAGTTTGATTTTAGTCAGACACGGGAGGATCTGTTAAGCAGGCTTGGAAATTTCGGACCCCTCCAATACAATATTATTTTAGCCAAAATTTCTAATTTTTTAATTTTTCATGAATTTTGGTAATATTTGTTCTAATTTAACTAAATTTTGTTCAAAATTTCGGTCTATCAGTGACCTCCGATCAAATCAGTTAAACCGAGAAAATAAACCATGCTCTTAAGAGAGTTTGGTATGGTTCAATATCAAAACTTATAGTCTTGCAATTTTTTCTACCCTTTATCTTTTTCCCTGACTATTTAGTATGGATCGTTTAAAAAAAAGAAAGCCCATTGGTGACCAAGGGCTTGTTTGATTCAAGACCATCCCTAGCCTTACCAACCTTTTGGCAATGGCAAAAATTGGTTGTTGCCAAAAATATTGGCACAAATTGGCTAAGCCTATGATTGGTTTCTACCAAAGTTGAATTTTGGCATTCAATCAAGCCAAATAATTTGGCAATAACATTTTCTTATCTATGGATATAACATATGGCAAATATTTTGGCATTACCATTTTCTTTTTGCCAAACATGTTATTCCTTTTGAATGACCAATCTTGACACCTTATGTATGTTAGTAGTGGAATCGACACTATTCTATCTAAAACATCTCTTTACATAGAGGCCGCTAATAATTTTTCTTTGAGATAACCAAATTTTCCTTACAAGTTAAGCAACAAAGCCCATTGGTAAGATATGCTACGACAAATGAACTAATAACTCCAAACGTAAAGCGGAGGATCCCGCATTTCCCACGTGGGTGACTCGAGCGGTGACAAACCCTAGTACCTCCACCCCCTTGGGTGGGTTGTGGTGGCACTTTCGGCACCGTATTTTCCTTGGACGGATCATTTAGAAAGTCCTATTATTGCCTAGTATGCCTTGACAGTTTAGGCAACACTCTTGGATGGTGGTGTCCTTTGCCCTGGTGATCTAGTAGCCCATGGATGTTTAGTTATTTGGACATGGTGTTGGATGGTGCGCTCGTGGGCCTGTTGTAGGTCTGGTGCCAACCAGTCATGCTTAGAAATAGCCGGATAGGTGCACAGTGCTAGTTCTTTACTTGGTGGTTTGTGCAGCGCTATCGACATGTGGTGGTGTGCTTTTTCTTTGTCCGGATAATAATCTCATAGGGCTATACTCTTGTTATTTTGCTGCTATATTATTATGATAACTTGGTATGGTTCGTTTTTTCTTTTTTTGGAAAAACACCTAGTTGATCAAGGGCTTGTTTGGTTCAAGTGCATTCCTAATCTTACCTTTTCTTTTTTTTTTCAATGGCAAGAATTGTTCATTGCAAAAAAAAAAAGAGATAAAAATTGGCTAGGCTTACGTTTTGGTTCTTACCAAAGTTGTACTTTGAGACCAAATATATGGCAAAATTTTGGCATAACCTTTTTTTTTTTTGCTTGGTTGAGCTTGGTACAAACCAATCAGTCACAAAATAGACTGTCATGAATCACGCCTACTAAATTCCTTTGAACCGAACTAGAATATATTTGCTCTTAAAAGATTTCTTGATTTCAATTGGTACCATTTACTAGTAGAAACTTAAATTTAAATTTTAAAAACAAAATCATAATATTGTTGTTATGGAAATTTTAGTCATTTTAGTACTTTTGTAATATATGAGTTGGGTTATACTTGAGATATCCTAAATTGCTTTAAGATGAACAATTGCTAGGTATATCAAAGATGAGCTAAAAACAATGCAGGCATTCCTTAGAGCTGCTGAAGTTATGAAAAAGAAAGATGAACTATTAAAGGTTTGGGCAGAGCAAATACGTGACCTGTCGTATGACATTGAAGATTCCCTTGATGAATTTAAAGTCCATATTGAAAGCCAAACCCTATTTCGTCAGTTGGTGAAACTTAGAGAGCGCCACCGGATCGCTATCCGTATCCACAACCTCAAATCAAGAGTTGAAGAAGTGAGTAGCAGGAACACACGCTACAATTTAGTCGAGCCTATTTCCTCCGGCACAGAGGATGACATGGATTCCTATGCAGAAGACATTCGCAATCAATCAGCTCGAAATGTGGATGAAGCTGAGCTTGTTGGGTTTTCTGACTCCAAGAAAAGGCTGCTTGAAATGATCGATACCAATGCTAATGATGGTCCGGCCAAGGTAATCTGTGTTGTTGGGATGGGTGGTTTAGGCAAGACAGCTCTTTCGAGGAAGATCTTTGAAAGCGAAGAAGACATTAGGAAGAACTTCCCTTGCAATGCTTGGATTACAGTGTCACAATCATTTCACAGGATTGAGCTACTTAAAGATATGATACGCCAACTTCTTGGCCCCAGTTCTCTGGATCAACTCTTGCAAGAATTGCAAGGGAAGGTGGTGGTGCAAGTACATCATCTTTCTGAGTACCTGATAGAAGAGCTCAAGGAGAAGAGGTACTTTGTTGTTCTAGATGATCTATGGATTTTACATGATTGGAATTGGATAAATGAAATTGCATTTCCTAAGAACAATAAGAAGGGCAGTCGAATAGTAATAACCACTCGGAATGTTGATCTAGCGGAGAAGTGTGCCACAGCCTCACTGGTGTACCACCTTGATTTCTTGCAGATGAACGATGCCATAACATTGCTACTGAGAAAAACAAATAAAAATCATGAAGACATGGAATCAAATAAAAATATGCAAAAGATGGTTGAACGAATTGTAAATAAATGTGGTCGTCTACCATTAGCAATACTTACAATAGGAGCTGTGCTTGCAACTAAACATGTGTCAGAATGGGAGAAATTCTATGAACAACTTCCTTCAGAACTAGAAATAAACCCAAGCCTGGAAGCTTTGAGGAGAATGGTGACCCTAGGTTACAACCACCTACCATCCCATCTGAAACCATGCTTTTTGTATCTAAGTATCTTTCCTGAGGATTTTGAAATCAAAAGGAATCGTCTAGTAGGTAGATGGATAGCAGAAGGGTTTGTTAGACCAAAGGTTGGGATGACGACTAAGGATGTCGGAGAAAGTTACTTTAATGAGCTAATCAACCGAAGTATGATTCAACGATCAAGAGTGGGCATAGCAGGAATAATTAAGACTTGTCGAATCCATGATATCATCCGTGATATCACAGTTTCAATCTCGAGACAGGAAAATTTTGTATTATTACCAATGGGAGATGGCTCTGATTTAGTTCAGGAAAACACTCGCCACATAGCATTCCATGGGAGTATGTCCTGCAAAACAGGATTGGATTGGAGCATTATTCGATCATTAGCTATTTTTGGTGACAGACCCAAGAGTCTAGCACATGCAGTTTGTCCAGATCAATTGAGGATGTTACGGGTCTTGGATCTTGAAGATGTGACATTCTTAATCACTCAAAAAGATTTCGACCGTATTGCATTGTTGTGCCACTTGAAATACTTGAGTATTGGATATTCGTCATCCATATATTCACTTCCCAGATCCATTGGTAAACTACAGGGCCTACAGACTTTGAACATGTCAAGCACATACATTGCAGCACTACCAAGTGAGATCAGTAAACTCCAATGTCTGCATACTCTTCGTTGTAGTAGAAAGTTTGTTTATGACAACTTTAGTCTAAACCACCCAATGAAGTGCATAACTAACACAATATGCCTGCCTAAAGTATTCACACCTTTAGTTAGTCGCGATGATCGTGCAAAACAAATTGCTGAATTGCACATGGCCACCAAAAGTTGCTGGTCTGAATCATTCGGTGTGAAGGTACCCAAAGGAATAGGTAAGTTGCGAGACTTGCAGGTTCTAGAGTATGTAGATATCAGGCGGACCAGTAGTAGAGCAATCAAAGAGCTGGGGCAGTTAAGCAAGTTGAGGAAATTAGGTGTGATAACAAAAGGCTCGACAAAGGAAAAATGTAAGATACTTTATGCAGCCATTGAGAAGCTCTCTTCCCTCCAATCTCTCTATGTGAATGCTGCGTTATTTTCAGGTATTGGAACACTTCAGTGTATAGATTCTATTTCATCTCCTCCTCCCCTACTGAGGACACTCGGGTTGAATGGAAGTCTTGAAGAGATGCCTAACTGGATTGAGCAGCTCACTCACCTGAAGAAGATCTACTTATTGAGGAGCAAACTAAAGGAAGGTAAAACCATGCTGATACTTGGGGCATTGCCCAACCTCATGGTCCTTGATCTTTATCATAATTCTTATCTTGGGGAGAAGCTAGTATTCAAAACGGGAGCATTCCCAAATCTTAGAACACTTTGGATTTATGAATTGGATCAGCTAAGAGAGATTAGATTTGAGGACGGCAGCTCGCCCCAGTTGGAAAAGATAGAAATCAGATTCTGCAGGTTGGAATCAGGGATTATTGGTATTATCCACCTTCCAAGGCTCAAGGAGATTTCACGTGGATACGAAAGTAAAGTGGCTGGGCTTGCTCAGCTGGAGGGAGAAGTGAACGCACACCCAAATCGCCCCGTGCTGCTAATGTACAGTGACGGAAGGTATCACGACCTGGGGGCTGAAGCCGAAGGATCTTCTATAGAAGTGCAAACAGCAGATCCTGTTCCTGATGCCGAAGGATCAGTCACTGTAGCAGTGGAAGCAACGGATCCCCTTCTCGAGCAGGAGGGAGAGAGCTCGCAGTCGCAGGTGATCACGTTGACGACGAATGATAGGTCAGTCACTCCCTACATGGCAGCTTAATTAACTTGTTTCTAATTCTCTTCTTGTTCAGTATTAGCCATCAGGTGAGGGCGATGATTTCAACTCACTTTTCATCTCTCTCGTTTTCTTAACTTGACA

>Pi9-Type2

GCTTGCATATGACGTTATGTAGATAGAGATGGCCAATATAATGCGCTGGAAAGTCCAAAGTGAGGATGCAAAACATCTTATAGTGGGTAGTGGAGCCATGCAAGGACCTGGTCTAAAGCGCACCTAAACCGTCATGTGGACTGCCATTATAGTTAAAGTTAGGGGGAATATGATTCTCTTCATGTGCACCTAAACTTTAATATGCAGTGAAACGAACGCTATGATATGATGATAAGCTTAATTCCTCTCTCTGCTCAGACTGTTCAGTGCAAAAGCTACCAACGAGCTTGTCTCCTTGTGCGGTCGTGAGCTTGCTTGTGCTAAGCTTGAAGGGAGAGTCGAACGAATCCATGGCGGAGACGGTGCTGAGCATGGCGAGGTCGCTGGTGGGCAGTGCCATCAGCAAGGCCGCCTCTGCCGCTGCCAATGAGACGAGCCTCCTGCTCGGCGTCGAGAAGGACATCTGGTACGTACTGCACTGCGCTCTCGTTTATCCTAGCTCGGTTGTATCGACTTCCAGCTTAATCTTTTTAATAATGAATAAAAACCCGGACTTGTTATCCATAAGTGGATATACACAGTCAAAACACGCGACAAGTTCTTAGGCTCTTAATTAATCTCGAAATTGAGGAACACCATGAAACACTAAAAGAGAGCTCGAAGACTAGGAAAGAAAACTAGAAGACTAAGCTTTGAAAGTCTTCTAAATCCAAGCATCTCGACATTGATCATCCTTGTGCAACATCAACCCTTCCTATTGCTTCACCAGAATCGGCGTCCCTTGTGGAGATCTCTGTTGTAACGTCAAGGGGAAAATCGGAGAAGCAGAACTAGTCCGCGCTGCCTTCGCTACGCCATCTCCGCCTTAGAGGATCTCATCCACGAAACATCCACCATCCAAACGGGAAACAGTTTTAAACACTCGTGGACGTTCACCCGTTCATCTAAATGGTTATGAAAAATTTTCAAAAAAAATAACATGATAGGTTAACATGTAATATATCATCTTATAAATATGCAAGTTCAAATTTGATTTCTACAAGTTGTAACAAAAATAACAAATTTTACTGTGAATATACGTAAACTAGTTAAAGTTTAATTTGTTATTTTTGTTACAACTTGTAGAAGTCGAATTTAAATCTGTATGTTTGTGAAATGATATATTACATATTAACCTATCTTATAATTTTTTTTAGAAAATTTTTTAGAATTATTTAGGTGGCATACAAGAAACGGGTGGACATCCACAAAGAGAGATTAGTATCCATCTCCACATCCAAACCCGTTGTTGCACCATCTGTCGAATCCGGCTGTGGACGCTCGGAGGCAAGAGCTAGCTCACCCGTCCCACACACACACCCAACGACGTCACAAGCGCCTCCGAACAACGCCAACTGATAACTTGGCAGCTCCTACGTGCCGACGTCGCGGTACTTGCCGGCGCTCCTAGCGCATGCACCGTCGAACCACACCGTCACCGACCAACTACCCACCGCCGCCGACTTCTGCCTCATCTGCCATCGTCGCCCTAGCCCAAGTTATCATCGTGGCAATTGCCGAGGCTCCTAAGTGTGCCACGGCCGAGGCAAAGTTCTAACTGAATCAGACAGCCACCACCGACACTTCTGCTTCATCTGCCATCGCCGTACTAGTTCAAGTTGTCGCTGTGGCAATCATTGGCCCTCCTAGCGTGCCACCCAACCGGACAACCACGACATCCCCCATCACTGTTGTTATTGCCGCGCCCTGACCCCTATCGTCGTCGCTCTTAGCGCGTCGTCGAGCCGACCAGCCACTGTCGTGCAGATGAAAAAAAAAACACATATTGGCCTGAGAGATCTGCTTAGTTCCAGTGCAGGTCCAACATGCTGTGAGATGCGGGCGTGCCAGTCAGTTTGATCTTGCAACTGACAAGATATATAAACAGCAGATAAAACAGCCGATCGACTAACAAGCCGATGGAGTAATTACAGCCGATAGCCGATATTAGCCGATGCCGATTCTAGCCGATGTCGATAGGGTTTTGAACTATCGGCTATATGTCTAATGTAGGCAATGATATAAAGACAATTGGCTGATGATAATAAAATATAAAAATATAATCCAATAGAAACCAATCGGCTAATAATAAATATTGATCCGATGGTTAAAGCATACATCGGCTAAAAGTCCGATGTCATAAAATCCAATCGATTTAGATAAACAGTGAAACCTTTTTTGCAATCGGCTAAATCCAACTTGTATGTAATCTTCGTAAGCCGATGAACGTCCAGATAACTTATCGGCTAGCACCTCGATAAAACACTAGCATGAACCTATCGGCTTAACAAGATTTATATTATCAACAACAATCTAGTAGGTCGGACCTAACCGATGCAACACGGATTAGATATGATAATCTAATACTCGATGAGCCAATAGATCTGTCTAATGTGATGGATATAACAAATCTATTTATAACAGCATTGCGATTGTAGAGATATATCGGCTAAGACAGAATATCAGACCTAACTAAACCGATGCGTCTCTAAACACAATGCAATTAATTAGAGATATAATTGAGATATCAGCTAGGCAAATATATCAACCAAACTGGAGCGATCCAAGAGATCGGAGCAATGCAGCCTTGAACAACACCAATGTAGCCGATGGATTCACCAGGGCCGACGGAACGTAGGACTTACCCCTTCGCTGAAGATCGGGCTGAACCAATGCAGCCCCGCGTCAGGTGCCAAATTCCGCCGGTTGATAAGTAAAACCTCAGAAAAGAGGATGACGATGCGCCGAGAGTAGTATTGATCGAGAGATAAATTTCAATGACCCTGGATGTACATATTTGTACCCATGGGTAGATATTAGTTCTTGTAGGACAAGAAAGAAACTTTCCTAAAGATAAAATGAAAACATAAAGTCTTTATTGGATACTAAATACACTTTCCTAAAGATAAAAGGAAACTAAACCCTGCCTAATTAATAGATAAACTGCCATGTCGTATCCTCCTTGAACTCGGACTCTTTTAGATAAGCTACCTTTAACTAATCTTTACCCAAATCCATCAAGAATACAAATGTTGGCATTGATAGGTTCCATCGGTCAATTCTAGGACTTTGAAGCCGATACTGACTCTAAGCCGATGATTACTTTGTGCTTACCAAATTTTGTTGTTAACATGTCGCAACCACCATCACCGGCCAGCCACCCTGATCATTGTTGTTGACTCAGCATTCGCCAGGCTGAGCAGTCCACATACATGCCGCCATCTCCATGGCACTGTCGTTGCCGCCCCTTTCTCCTAGAGCCGCCGCAGCGCTCTTCGACACACCTACTGCATCGTCGAGCAGTCGTGCTACCACCTCCTCCATCGACCATAGCCGCCTCTTCTGCTGCACCGGATCCACCCACACCAACCACCAGATACAGTCAAGCCCTCATTCCCGGATCCCATATCCATCCATGCCACTACTGTGCTGCCCAGTCCAAGGAATGGAGCGAAGGAGGAAGCCCCGCCGCTGCCCTCCCGGCGGCCACATGCACTCCAGTGCCTTGCTCCGACGGCAGCGAGGTTGGAAAATGGGTGGCAGCGGCTAGGGTTTATCTGGGGAGAAGGAAAAGGAGAGGGAGGGGGGGGGGGTCCACTTCCAGCTTAATTAGCCTAGATCTTATTGACAAATCAGTTGCTGGGTGCACAGACATGTTATTCTTTTTGCATGACCAATCTTGAACACTTAGGTATGTTAGTTGAGTGGACACTGGTCTATCTGAAACATCTCTTCACATGGAGGCTGCGAATGAGTTTTCTTTTTGAGAGACCAAAGTTTCGTTGTATGTTAAGTGATAAAGCCTTGGTAAGAAATGCTACCACAAACGAACTAATAACTCCAAACGTAAAGTGGAGGAACCCGTATGGGTGACTCGAGTGGCGACAAACTCTAGCACCTCCACCTCCTTGGACGGGCTGCGGCGGTGCTTTCGGCATCCCAGTCTTCTTGGAGGCATCATCTAGAATTAAGGTCTTGTTATTGCTTAGCATGCCTTAGGGCACGTCCAGTGTTTAGTTCGACTAAAACTTCCATGAAAGCCAAACAAAAGTTCTGTTTGACCACCACAGTGTAAAAATCGATTGTGGGACCCATGCAAAAAAATCACAATCTCAGCTGCCTATGCTCTCCTCCTGGACCTGATAGCCGTGCACAACAAATATTTTTTTAAACTGGATGTGTTCGGCTTCTCTTTAAAGATCGTTTTTTCCTCTGACACTTACCAACCGGCTTTCACAGTGTGGTCAGTTCTTTTTTTTTTTACGCAAAGTTTGATTTTAGTCAGACACGGGAGGATCTGTTAAGCAGGCTTGGAAATTTCGGACCCCTCCAATACAATATTATTTTAGCCAAAATTTCTAATTTTTTAATTTTTCATGAATTTTGGTAATATTTGTTCTAATTTAACTAAATTTTGTTCAAAATTTCGGTCTATCAGTGACCTCCGATCAAATCAGTTAAACCGAGAAAATAAACCATGCTCTTAAGAGAGTTTGGTATGGTTCAATATCAAAACTTATAGTCTTGCAATTTTTTCTACCCTTTATCTTTTTCCCTGACTATTTAGTATGGATCGTTTAAAAAAAAGAAAGCCCATTGGTGACCAAGGGCTTGTTTGATTCAAGACCATCCCTAGCCTTACCAACCTTTTGGCAATGGCAAAAATTGGTTGTTGCCAAAAATATTGGCACAAATTGGCTAAGCCTATGATTGGTTTCTACCAAAGTTGAATTTTGGCATTCAATCAAGCCAAATAATTTGGCAATAACATTTTCTTATCTATGGATATAACATATGGCAAATATTTTGGCATTACCATTTTCTTTTTGCCAAACATGTTATTCCTTTTGAATGACCAATCTTGACACCTTATGTATGTTAGTAGTGGAATCGACACTATTCTATCTAAAACATCTCTTTACATAGAGGCCGCTAATAATTTTTCTTTGAGATAACCAAATTTTCCTTACAAGTTAAGCAACAAAGCCCATTGGTAAGATATGCTACGACAAATGAACTAATAACTCCAAACGTAAAGCGGAGGATCCCGCATTTCCCACGTGGGTGACTCGAGCGGTGACAAACCCTAGTACCTCCACCCCCTTGGGTGGGTTGTGGTGGCACTTTCGGCACCGTATTTTCCTTGGACGGATCATTTAGAAAGTCCTATTATTGCCTAGTATGCCTTGACAGTTTAGGCAACACTCTTGGATGGTGGTGTCCTTTGCCCTGGTGATCTAGTAGCCCATGGATGTTTAGTTATTTGGACATGGTGTTGGATGGTGCGCTCGTGGGCCTGTTGTAGGTCTGGTGCCAACCAGTCATGCTTAGAAATAGCCGGATAGGTGCACAGTGCTAGTTCTTTACTTGGTGGTTTGTGCAGCGCTATCGACATGTGGTGGTGTGCTTTTTCTTTGTCCGGATAATAATCTCATAGGGCTATACTCTTGTTATTTTGCTGCTATATTATTATGATAACTTGGTATGGTTCGTTTTTTCTTTTTTTGGAAAAACACCTAGTTGATCAAGGGCTTGTTTGGTTCAAGTGCATTCCTAATCTTACCTTTTCTTTTTTTTTTGAATGGCAAGAATTGTTCATTGCAAAAAAAAAAAGAGATAAAAATTGGCTAGGCTTACGTTTTGGTTCTTACCAAAGTTGTACTTTGAGACCAAATATATGGCAAAATTTTGGCATAACCTTTTTTTTTTTTGCTTGGTTGAGCTTGGTACAAACCAAACAGTCACAAAATAGACTGTCATGAATCACGCCTACTAAATTCCTTTGAACCGAACTAGAATATATTTGCTCTTAAAAGATTTCTTGATTTCAATTGGTACCATTTACTAGTAGAAACTTAAATTTAAATTTTAAAAACAAAATCATAATATTGTTGTTATGGAAATTTTAGTCATTTTAGTACTTTTGTAATATATGAGTTGGGTTATACTTGAGATATCCTAAATTGCTTTAAGATGAACAATTGCTAGGTATATCAAAGATGAGCTAAAAACAATGCAGGCATTCCTTAGAGCTGCTGAAGTTATGAAAAAGAAAGATGAACTATTAAAGGTTTGGGCAGAGCAAATACGTGACCTGTCGTATGACATTGAAGATTCCCTTGATGAATTTAAAGTCCATATTGAAAGCCAAACCCTATTTCGTCAGTTGGTGAAACTTAGAGAGCGCCACCGGATCGCTATCCGTATCCACAACCTCAAATCAAGAGTTGAAGAAGTGAGTAGCAGGAACACACGCTACAATTTAGTCGAGCCTATTTCCTCCGGCACAGAGGATGACATGGATTCCTATGCAGAAGACATTCGCAATCAATCAGCTCGAAATGTGGATGAAGCTGAGCTTGTTGGGTTTTCTGACTCCAAGAAAAGGTTGCTTGAAATGATCGATACCAATGCTAATGATGGTCCGGCCAAGGTAATCTGTGTTGTTGGGATGGGTGGTTTAGGCAAGACAGCTCTTTCGAGGAAGATCTTTGAAAGCGAAGAAGACATTAGGAAGAACTTCCCTTGCAATGCTTGGATTACAGTGTCACAATCATTTCACAGGATTGAGCTACTTAAAGATATGATACGCCAACTTCTTGGCCCCAGTTCTCTGGATCAACTCTTGCAAGAATTGCAAGGGAAGGTGGTGGTGCAAGTACATCATCTTTCTGAGTACCTGATAGAAGAGCTCAAGGAGAAGAGGTACTTTGTTGTTCTAGATGATCTATGGATTTTACATGATTGGAATTGGATAAATGAAATTGCATTTCCTAAGAACAATAAGAAGGGCAGTCGAATAGTAATAACCACTCGGAATGTTGATCTTGCGGAGAAGTGTGCCACAGCCTCACTGGTGTACCACCTTGATTTCTTGCAGATGAACGATGCCATAACATTGCTACTGAGAAAAACAAATAAAAATCATGAAGACATGGAATCAAATAAAAATATGCAAAAGATGGTTGAACGAATTGTAAATAAATGTGGTCGTCTACCATTAGCAATACTTACAATAGGAGCTGTGCTTGCAACTAAACAGGTGTCAGAATGGGAGAAATTCTATGAACAGCTTCCTTCAGAACTAGAAATAAACCCAAGCCTGGAAGCTTTGAGGAGAATGGTGACCCTAGGTTACAACCACCTACCATCCCATCTGAAACCATGCTTTTTGTATCTAAGTATCTTTCCTGAGGATTTTGAAATCAAAAGGAATCGTCTGGTAGGTAGATGGATCGCAGAAGGGTTTGTCGGACCGAAGGTTGGGATGACGATTAAGGATGTTGGAAAAAGTTACTTTTATGAGCTAATCAACCGAAGTATGATTCAACGATCAAGAGTGGGCATAGAAGGAAAAATAAAGAGTTGTCGAGTCCATGATATCATGCGTGATATCACAGTTTCAATCTCGAGACAGGAAAACTTTGTATTATTACCAATGGACGATGGCTCTGATTTAGTTCAGGAAAACACTCGCCACATAGCATTCCATGGGAGTATGTCCTGCAAAACAGGATTGGATTGGAGCATTATTCGATCATTAACTATTTTTGGTGATAGACCCAAGAGTCTAGCACATGCAGTTTGTTCAGATCAATTGAGGATGTTACGTGTCTTGGATCTTGAAGATGTGAAATTCTTAAGCACTCAAAAAGATTTCAACAATATAGCATTGTTGCGCCACTTGAAATACTTGAGTATTGGAAATTCGTCATGCATATATTCACTTCCCAGATCGATTGGTAAACTACAGGGCCTACAGACTTTGAACATGTCAAGCACATACATTGCAGCACTACCAAGTGAGATCAGTAAACTCCAATGTCTACATACTCTTCGTTGTATAAGAGAGCTTGATTTTGACAAATTTAGTCTAAATCGCCCAATGAAGTGCATAACTAACACAATATGCCTGCCTAAAGTATTCACACCTTTAGTTAGTCGCGATAATCGTGCAAAACAAATTGCTGAATTTCACATGGCCACCAAAAGTTTCTGGTCTGAATCATTCGGTGTGAAGGTACCCAAAGGAATAGGTAAGTTGCGAGACTTACAGGTTCTAGAGTATGTAGATATCAGGCGGACCAGTAGTAGAGCAATCAAAGAGCTGGGGCAGTTAAGCAAGTTGAGGAAATTAGGTGTGATAACAAAAGGCTCGACAAAGGAAAAATGTAAGATACTTTATGCAGCCATTGAGAAGCTCTCTTCCCTCCAATCTCTCTATGTGAATGCTGCGTTATTATCAGATATTGAAACACTTGAGTGCCTAGATTCTATTTCATCTCCTCCTCCCCTACTGAGGACACTCGGGTTGAATGGAAGTCTTGAAGAGATGCCTAACTGGATTGAGCAGCTCACTCACCTGAAGAAGTTCTACTTATGGAGGAGCAAACTAAAGGAAGGTAAAACCATGCTGATACTTGGGGCACTGCCCAACCTCATGTTCCTTTCTCTTTATCATAATTCTTATCTTGGGGAGAAGCTAGTATTCAAAACGGGAGCATTCCCAAATCTTAGAACACTTTGGATTTATGAATTGGATCAGCTAAGAGAGATCAGATTTGAGGACGGCAGCTCACCCCTGTTGGAAAAGATAGAAATAGGCGAGTGCAGGTTGGAATCTGGGATTATTGGTATTATCCACCTTCCAAGGCTCAAGGAGATTTCACTTCGATACGAAAGTAAAGTGGCTGGGCTTGCTCAGCTGGAGGGAGAAGTGAACGCACACCCAAATCGCCCCGTGCTGCTAATGTACAGTGACCGAAGGTATCACGACCTGGGGGCTGAAGCCGAAGGATCTTCTATAGAAGTGCAAACAGCAGATCCTGTTCCTGATGCCGAAGGATCAGTCACTGTAGCAGTGGAAGCAACGGATCCCCTTCCCGAGCAGGAGGGAGAGAGCTCGCAGTCGCAGGTGATCACGTTGACGACGAATGATAGGTCAGTCACTCCCTACATGGCAGCTTAATTAACTTGTTTCTAATTCTCTTCTTGTTCAGTATTAGCCATCAGGTGAGGGCGATGATTTCAACTCACTTTTCATCTCTCTCGTTTTCTTAACCTGACA

>Pi9-Type3

GCTTGCATATGACGTTATGTAGATAGAGATGGCCAATATAATGCGCTGGAAAGTCCAAAGTGAGGATGCAAAACATCTTATAGTGGGTAGTGGAGCCATGCAAGGACCTGGTCTAAAGCGCACCTAAACCGTAATGTGGACTTTAAAGTTAGGGGGAATATGATTCTCTTCATGTGCACCTAAACCGTAATATGCAGTGAAACGAACGCTATGATATGATGATAAGCTTAATTCCTCTCTCTGCTCAGACTGTTCAGTGCAAAAGCTACCAACGAGCTTGTCTCCTTGTGCGGTCGTGAGCTTGCTTGTGCTAAGCTTGAAGGGAGAGTCGAACGAATCCATGGCGGAGACGGTGCTGAGCATGGCGAGGTCGCTGGTGGGCAGTGCCATCAGCAAGGCCGCCTCTGCCGCTGCCAATGAGACGAGCCTCCTGCTCGGCGTCGAGAAGGACATCTGGTACGTACTGCACTGCTCTCGTTTATCCTAGCAAGTTCTTAGGCTCTTAATCTCGAAATTGAGGAACACCATGAAACACTAAAAGAGAGCTCGAAGACTAGGAAAGAAAACTAGAAGACTAAGCTTTGAAAGTCTTCTAAATCCAAGCATCTCGACATTGATCATCCTTGTGCAACATCATCCCTTCCTATTGCTTCACCAGAATCGGTGTCCCTTGTGGAGATCTCTGTCGTAGCGTCAAGGGGAGAATCCGAGAAGCAGAACTAGTCCGCGCTGCCTTCGCTACGCCATCTCCGCCATAGAGGATCTCATCCACGAAACATCCACCATCCAAACGGGAAACTGTTTTAAACACTCGGGTGGATGTTCACCCGTTTCTTGCATATCATCTAAATGGTTATGAAAAATTTTCAAAAAAAAAACATGATAGGTTAATATATAATATATCATCTCACAAATATGCAAGTTCAAATTCAACTTTTATAAGTTGTAAGTATAACAGGACGTTCATCTCACAAATATGCAAGTTTAAATTTAACTTTTACAAGTTGTAAGTGTAACAGTACGTCCATCGGATAGATTAATATCCATCTCCCCATCCAAACCCGTTGTTGCACCATCTGTCGAATCCGGCTGTGGACGCTCGGAGGCAAGAGCTAGCTCACCCGTCCCACACACACACCCAACGACGTCACAAGCGCCTCCGAACAACGCCAACTGATAACTTGGCAGCTCCTACGTGCCGACGTCGCGGTACTTGCCGGCGCTCCTAGCGCACGCACCGTCGAACCACACCGTCACCGACCAACTACCCACCGCCGCCGACTTCTGCCTCATCTGCCATCGTGGCCCTAGCCCAAGTTATCATCGTGGCAATTGCCGAGGCTCCTAAGTGTGCCACGGCCGAGGCAAAGTTCTAACTGAATCAGAGTATCAGACAGCCACCACCGACGCTACTTCTGCTTCATCTGCCATCGCCGTACTAGTTCAAGTTGTCGCTGTGGCAATCATGGGCCCTCCTAGCGTGCCACCCAACCGGACAGCCACGACATCCCCCATCACTGTTGTTATTGCCGCGCCCTGACCCCTATCGTCGTCGCTCTTAGCGCGTCGACGAGCCGACCAGCCACTGTCGTGCAGATGAAAAAAAAAACACATATGGGCCTGAGAGATCTGCTTAGTTCCAGTGCAGGTCCAACATGCTGTGAGATGCGGGCGTGCTAGTCAGTTTGATCTTGCAACTGACAAGATATATAAACAGCAGATAAAACAGCCGATCGACTAACAAGCCGATGGAGTAATTCCAGCCGATAGCCGATATTAGCCGATGCCGATTCTAGCCGATGTCGATAGGGTTTTGAACTATCGGCTATATGTCTAATGTAGGCAATGATATAAAGACAATTGGCTGATGATAATAAAATATAAAAATATAATCCAATAGAAACCAATCGGCTAATAATAAATATTGATCCGATGGTTAAAGCATACATCGGCTAAAAGTCCGATGTCATAAAATCCAATCGATTTAGATAAACAGTGAAACCTTTTTTGCAATCGGCTAAATCCAACTTGTATGTAATCTTCGTAAGCCGATGAACGTCCAGATAACTTATCGGCTAGCACCTCGATAAAACACTAGCATGAACCTATCGGCTTAACAAGATTTATATTATCAACAACAATCTAGTAGGTCGGACCTAACCGATGCAACACGGATTAGATATGATAATCTAATACTCGATGAGCCAATAGATCTGTCTAATGTGATGGATATAACAAATCTATTTATAACAGCATTGCGATTGTAGAGATATATCGGCTAAGACAGAATATCAGACCTAACTAAACCGATGCGTCTCTAAACACAATGCAATTAATTAGAGATATAATTGAGATATCAGCTAGGCAAATATATCAACCAAACTGGAGCGATCCAAGAGATCGGAGCAATGCAGCCTTGAACAACACCAATGTAGCCGATGGATTCACCAGGGCCGACGGAACGTAGGACTTACCCCTTCGCTGAAGATCGGGCTGAACCAATGCAGCCCCGCGTCAGGTGCCAAATTCCGCCGGTTGATAAGTAAAACCTCAGAAAAGAGGATGACGATGCGCCGAGAGTAGTATTGATCGAGAGATAAATTGCAATGACCCTGGATGTACATATTTGTACCCATGGGTAGATATTAGTTCTTGTAGGACAAGAAAGAAACTTTCCTAAAGATAAAATGAAAACATAAAGTCTTTATTGGATACTAAATACACTTTCCTAAAGATAAAAGGAAACTAAACCCTGCCTAATTAATAGATAAACTGCCATGTCGTATCCTCCTTGAACTCGGACTCTTTTAGATAAGCTACCTTTAACTAATCTTTACCCGAATCCATCAAGAATACAAATGTTGGCATTGATAGTTTCCATCGGTCAATTCTAGGACTTTGAAGCCGATACTGACTCTAAGCCGATGATTACTTTGTGCTTACCAAATTTTGTTGTTAACATGTCGCAACCACCATCACCGGCCAGCCACCCTGATCATTGTTGTTGACTCAGCATTCGCCAGGCTGAGCAGTCCACATACATGCCGCCATCTCCATGGCACTGTCGTTGCCGCCCCTTTCTCCTAGAGCCGCCGCAGCGCTCTTCGACACACCTACTGCATCGTCGAGCAGTCGTGCTACCACCTCCTCCATCGACCATAGCCGCCTCTTCTGCTGCACCGGATCCACCCACACCAACCACCAGATACAGTCAAGCCCTCATTCCCGGATCCCATATCCATCCATGCCACTACTGTGCTGCCCAGTCCAAGGAATGGAGCGAAGGAGGAAGCCCCGCCGCTGCCCTCCCGGCGGCCACATGCACTCCAGTGCCTTGCTCCGACGGCAGCGAGGTTGGAAAATGGGTGGCAGCGGCTAGGGTTTATCTGGGGAGAAGGAAAAGGAGAGGGAGGGGGGGGGGGTCCACTTCCAGCTTAATTAGCCTAGATCTTATTGACAAATCAGTTGCTGGGTGCACAGACATGTTATTCTTTTTGCATGACCAATCTTGAACACTTAGGTATGTTAGTTGAGTGGACACTGGTCTATCTGAAACATCTCTTCACATGGAGGCTGCGAATGAGTTTTCTTTTTGAGAGACCAAAGTTTCGTTGTATGTTAAGTGATAAAGCCTTGGTAAGAAATGCTACCACAAACGAACTAATAACTCCAAACGTAAAGTGGAGGAACCCGTATGGGTGACTCGAGTGGCGACAAACTCTAGCACCTCCACCTCCTTGGACGGGCTGCGGCGGTGCTTTCGGCATCCCAGTCTTCTTGGAGGCATCATCTAGAATTAAGGTCTTGTTATTGCTTAGCATGCCTTAGGGCACGTCCAGTGTTTAGTTCGACTAAAACTTCCATGAAAGCCAAACAAAAGTTCTGTTTGACCACCACAGTGTAAAAATCGATTGTGGGACCCATGCAAAAAAATCACAATCTCAGCTGCCTATGCTCTCCTCCTGGACCTGATAGCCGTGCACAACAAATATTTTTTTAAACTGGATGTGTTCGGCTTCTCTTTAAAGATCGTTTTTTCCTCTGACACTTACCAACCGGCTTTCACAGTGTGGTCAGTTCTTTTTTTTTTTACGCAAAGTTTGATTTTAGTCAGACACGGGAGGATCTGTTAAGCAGGCTTGGAAATTTCGGACCCCTCCAATACAATATTATTTTAGCCAAAATTTCTAATTTTTTAATTTTTCATGAATTTTGGTAATATTTGTTCTAATTTAACTAAATTTTGTTCAAAATTTCGGTCTATCAGTGACCTCCGATCAAATCAGTTAAACCGAGAAAATAAACCATGCTCTTAAGAGAGTTTGGTATGGTTCAATATCAAAACTTATAGTCTTGCAATTTTTTCTACCCTTTATCTTTTTCCCTGACTATTTAGTATGGATCGTTTAAAAAAAAGAAAGCCCATTGGTGACCAAGGGCTTGTTTGATTCAAGACCATCCCTAGCCTTACCAACCTTTTGGCAATGGCAAAAATTGGTTGTTGCCAAAAATATTGGCACAAATTGGCTAAGCCTATGATTGGTTTCTACCAAAGTTGAATTTTGGCATTCAATCAAGCCAAATAATTTGGCAATAACATTTTCTTATCTATGGATATAACATATGGCAAATATTTTGGCATTACCATTTTCTTTTTGCCAAACATGTTATTCCTTTTGAATGACCAATCTTGACACCTTATGTATGTTAGTAGTGGAATCGACACTATTCTATCTAAAACATCTCTTTACATAGAGGCCGCTAATAATTTTTCTTTGAGATAACCAAATTTTCCTTACAAGTTAAGCAACAAAGCCCATTGGTAAGATATGCTACGACAAATGAACTAATAACTCCAAACGTAAAGCGGAGGATCCCGCATTTCCCACGTGGGTGACTCGAGCGGTGACAAACCCTAGTACCTCCACCCCCTTGGGTGGGTTGTGGTGGCACTTTCGGCACCGTATTTTCCTTGGACGGATCATTTAGAAAGTCCTATTATTGCCTAGTATGCCTTGACAGTTTAGGCAACACTCTTGGATGGTGGTGTCCTTTGCCCTGGTGATCTAGTAGCCCATGGATGTTTAGTTATTTGGACATGGTGTTGGATGGTGCGCTCGTGGGCCTGTTGTAGGTCTGGTGCCAACCAGTCATGCTTAGAAATAGCCGGATAGGTGCACAGTGCTAGTTCTTTACTTGGTGGTTTGTGCAGCGCTATCGACATGTGGTGGTGTGCTTTTTCTTTGTCCGGATAATAATCTCATAGGGCTATACTCTTGTTATTTTGCTGCTATATTATTATGATAACTTGGTATGGTTCGTTTTTTCTTTTTTTGGAAAAACACCTAGTTGATCAAGGGCTTGTTTGGTTCAAGTGCATTCCTAATCTTACCTTTTCTTTTTTTTTTTAATGGCAAGAATTGTTCATTGCAAAAAAAAAAAGAGATAAAAATTGGCTAGGCTTACGTTTTGGTTCTTACCAAAGTTGTACTTTGAGACCAAATATATGGCAAAATTTTGGCATAACCTTTTTTTTTTTTGCTTGGTTGAGCTTGGTACAAACCAAACAGTCACAAAATAGACTGTCATGAATCACGCCTACTAAATTCCTTTGAACCGAACTAGAATATATTTGCTCTTAAAAGATTTCTTGATTTCAATTGGTACCATTTACTAGTAGAAACTTAAATTTAAATTTTAAAAACAAAATCATAATATTGTTGTTATGGAAATTTTAGTCATTTTAGTACTTTTGTAATATATGAGTTGGGTTATACTTGAGATATCCTAAATTGCTTTAAGATGAACAATTGCTAGGTATATCAAAGATGAGCTAAAAACAATGCAGGCATTCCTTAGAGCTGCTGAAGTTATGAAAAAGAAAGATGAACTATTAAAGGTTTGGGCAGAGCAAATACGTGACCTGTCGTATGACATTGAAGATTCCCTTGATGAATTTAAAGTCCATATTGAAAGCCAAACCCTATTTCGTCAGTTGGTGAAACTTAGAGAGCGCCACCGGATCGCTATCCGTATCCACAACCTCAAATCAAGAGTTGAAGAAGTGAGTAGCAGGAACACACGCTACAATTTAGTCGAGCCTATTTCCTCCGGCACAGAGGATGACATGGATTCCTATGCAGAAGACATTCGCAATCAATCAGCTCGAAATGTGGATGAAGCTGAGCTTGTTGGGTTTTCTGACTCCAAGAAAAGGCTGCTTGAAATGATCGATACCAATGCTAATGATGGTCCGGCCAAGGTAATCTGTGTTGTTGGGATGGGTGGTTTAGGCAAGACAGCTCTTTCGAGGAAGATCTTTGAAAGCGAAGAAGACATTAGGAAGAACTTCCCTTGCAATGCTTGGATTACAGTGTCACAATCATTTCACAGGATTGAGCTACTTAAAGATATGATACGCCAACTTCTTGGCCCCAGTTCTCTGGATCAACTCTTGCAAGAATTGCAAGGGAAGGTGGTGGTGCAAGTACATCATCTTTCTGAGTACCTGATAGAAGAGCTCAAGGAGAAGAGGTACTTTGTTGTTCTAGATGATCTATGGATTTTACATGATTGGAATTGGATAAATGAAATTGCATTTCCTAAGAACAATAAGAAGGGCAGTCGAATAGTAATAACCACTCGGAATGTTGATCTTGCGGAGAAGTGTGCCACAGCCTCACTGGTGTACCACCTTGATTTCTTGCAGATGAACGATGCCATAACATTGCTACTGAGAAAAACAAATAAAAATCATGAAGACATGGAATCAAATAAAAATATGCAAAAGATGGTTGAACGAATTGTAAATAAATGTGGTCGTCTACCATTAGCAATACTTACAATAGGAGCTGTGCTTGCAACTAAACAGGTGTCAGAATGGGAGAAATTCTATGAACACCTTCCTTCAGAACTAGAAATAAACCCAAGCCTGGAAGCTTTGAGGAGAATGGTGACCCTAGGTTACAACCACCTACCATCCCATCTGAAACCATGCTTTTTGTATCTAAGTATCTTTCCTGAGGATTTTGAAATCAAAAGGAATCGTCTAGTAGGTAGATGGATAGCAGAAGGGTTTGTTAGACCAAAGGTTGGGATGACGACTAAGGATGTCGGAGAAAGTTACTTTAATGAGCTAATCAACCGAAGTATGATTCAACGATCAAGAGTGGGCATAGCAGGAAAAATTAAGACTTGTCGAATTCATGATATCATCCGTGATATCACAGTTTCAATCTCGAGACAGGAAAATTTTGTATTGTTACCAATGGGAGATGGCTCTGATTTAGTTCAGGAAAACACTCGCCACATAGCATTCCATGGGAGTATGTCCTGCAAAACAGGATTGGATTGGAGCATTATTCGATCATTAGCTATTTTTGGTGACAGACCCAAGAGTCTAGCACATGCAGTTTGTCCAGATCAATTGAGGATGTTACGGGTCTTGGATCTTGAAGATGTGACATTCTTAATCACTCAAAAAGATTTCGACCATATTGCATTGTTGTGCCACTTGAAATACTTGAGTATTGGATATTCGTCATCCATATATTCACTTCCCAGATCCATTGGTAAACTACAGGGCCTACAAACTTTGAACATGCCGAGCACATACATTGCAGCACTACCAAGTGAGATCAGTAAACTCCAATGTCTGCATACTCTTCGTTGTATAGGACAGTTTCATTATGACAACTTTAGTCTAAACCACCCAATGAAGTGCATAACTAACACAATATGCCTGCCTAAAGTATTCACACCTTTAGTTAGTCGCGATGATCGTGCAAAACAAATTGCTGAATTGCACATGGCCACCAAAAGTTGCTGGTCTGAATCATTCGGTGTGAAGGTACCCAAAGGAATAGGTAAGTTGCGAGACTTACAGGTTCTAGAGTATGTAGATATCAGGCGGACCAGTAGTAGAGCAATCAAAGAGCTGGGGCAGTTAAGCAAGCTGAGGAAATTAGGTGTGACAACAAACGGGTCGACAAAGGAAAAATGTAAGATACTTTATGCAGCCATTGAGAAGCTCTCTTCCCTCCAATCTCTCCATGTGGATGCTGTGTTATTCTCAGGTATTATTGGAACACTTGAGTGCCTAGATTCTATTTCATCTCCTCCTCCCCTACTAAGGACACTCAGGTTGAATGGAAGTCTTGAAGAGATGCCTAACTGGATTGAGCAGCTCACTCACCTGAAGAAGTTCGACTTACGGAGGAGTAAACTAAAGGAAGGTAAAACCATGCTGATACTTGGGGCATTGCCCAACCTCATGGTCCTTTATCTTTATCGGAATGCTTACCTTGGGGAGAAGCTAGTATTCAAAACGGGAGCATTCCCAAATCTTAGAACACTTTGTATTTACGAATTGGATCAGCTAAGAGAGATCAGATTTGAGGACGGCAGCTCACCCCTGTTGGAAAAGATAGAAATAGGCAAGTGCAGGTTGGAATCTGGGATTATTGGTATCATTCACCTTCCAAAGCTCAAGGAGATTCCAATTACATACGGAAGTAAAGTGGCTGGGCTTGGTCAGCTGGAGGGAGAAGTGAACACACACCCAAATCGCCCCGTGCTGCTAATGTACAGTGACCGAAGGTATCACGACCTGGGGGCTGAAGCCGAAGGATCTTCTATAGAAGTGCAAACAGCAGATCCTGTTCCTGATGCCGAAGGATCAGTCACTGTAGCAGTGGAAGCAACGGATCCCCTTCCCGAGCAGGAGGGAGAGAGCTCGCAGTCGCAGGTGATCACGTTGACGACGAATGATAGGTCAGTCACTCCCTACATGGCAGCTTAATTAACTTGTTTCTAATTCTCTTCTTGTTCAGTATTAGCCATCAGGTGAGGGCGATGATTTCAACTCACTTTTCATCTCTCTCGTTTTCTTAACCTGACA

>Pi9-Type4

GCTTGCATATGACGTTATGTAGATAGAGATGGCCAATATAATGCGCTGGAAAGTCCAAAGTGAGGATGCAAAACATCTTATAGTGGGTAGTGGAGCCATGCAAGGACCTGGTCTAAAGCGCACCTAAACCGTCATGTGGACTGCCATTATAGTTAAAGTTAGGGGGAATATGATTCTCTTCATGTGCACCTAAACTTTAATATGCAGTGAAACGAACGCTATGATATGATGATAAGCTTAATTCCTCTCTCTGCTCAGACTGTTCAGTGCAAAAGCTACCAACGAGCTTGTCTCCTTGTGCGGTCGTGAGCTTGCTTGTGCTAAGCTTGAAGGGAGAGTCGAACGAATCCATGGCGGAGACGGTGCTGAGCATGGCGAGGTCGCTGGTGGGCAGTGCCATCAGCAAGGCCGCCTCTGCCGCTGCCAATGAGACGAGCCTCCTGCTCGGCGTCGAGAAGGACATCTGGTACGTACTGCACTGCGCTCTCGTTTATCCTAGCTCGGTTGTATCGACTTCCAGCTTAATCTTTTTAATAATGAATAAAAACCCGGACTTGTTATCCATAAGTGGATATACACAGTCAAAACACGCGACAAGTTCTTAGGCTCTTAATTAATCTCGAAATTGAGGAACACCATGAAACACTAAAAGAGAGCTCGAAGACTAGGAAAGAAAACTAGAAGACTAAGCTTTGAAAGTCTTCTAAATCCAAGCATCTCGACATTGATCATCCTTGTGCAACATCAACCCTTCCTATTGCTTCACCAGAATCGGCGTCCCTTGTGGAGATCTCTGTTGTAACGTCAAGGGGAAAATCGGAGAAGCAGAACTAGTCCGCGCTGCCTTCGCTACGCCATCTCCGCCTTAGAGGATCTCATCCACGAAACATCCACCATCCAAACGGGAAACAGTTTTAAACACTCGTGGACGTTTACCCGTTCATCTAAATGGTTATGAAAAATTTTCAAAAAAAATAACATGATAGGTTAACATGTAATATATCATCTTATAAATATGCAAGTTCAAATTTGATTTCTACAAGTTGTAACAAAAATAACAAATTTTACTGTGAATATACGTAAACTAGTTAAAGTTTAATTTGTTATTTTTGTTACAACTTGTAGAAGTCGAATTTAAATATGTATGTTTGTGAAATGATATATTACATATTAACCTATCTTATAATTCTTTTTAGAAATTTTTTATAATTATTTAGGTGGCATACAAGAAACGGGTGGACATCCACAAAGAGATTAGTATCCATCTCCACATCCAAACCCGTTGTTGCACCATCTGTCGAATCTGTCGAATCCGGCTGTGGACGCTCGGAGGCAAGAGCTAGCTCACCCGTCCCACACACACACCCAACGACGTCACAAGCGCCTCCGAACAACGCCAACTGATAACTTGGCAGCTCCTACGTGCCGACGTCGCGGTACTTGCCGGCGCTCCTAGCGCATGCACCGTCGAACCACACCGTCACCGACCAACTACCCACCGCCGCCGACTTCTGCCTCATCTGCCATCGTCGCCCTAGCCCAAGTTATCATCGTGGCAATTGCCGAGGCTCCTAAGTGTGCCACGGCCGAGGCAAAGTTCTAACTGAATCAGACAGCCACCACCGACACTTCTGCTTCATCTGCCATCGCCGTACTAGTTCAAGTTGTCGCTGTGGCAATCATTGGCCCTCCTAGCGTGCCACCCAACCGGACAACCACGACATCCCCCATCACTGTTGTTATTGCCGCGCCCTGACCCCTATCGTCGTCGCTCTTAGCGCGTCGTCGAGCCGACCAGCCACTGTCGTGCAGATGAAAAAAAAAAACACATATGGGCCTGAGAGATCTGCTTAGTTCCAGTGCAGGTCCAACATGCTGTGAGATGCGGGCGTGCCAGTCAGTTTGATCTTGCAACTGACAAGATATATAAACAGCAGATAAAACAGCCAATCGACTAACAAGCCGATGGAGTAATTCCAGCCGATAGCCGATATTAGCCGATGCCGATTCTAGCCGATGTCGATAGGGTTTTGAACTATCGGCTATATGTCTAATGTAGGCAATGATATAAAGACAATTGGCTGATGATAATAAAATATAAAAATATAATCCAATAGAAACCAATCGGCTAATAATAAATATTGATCCGATGGTTAAAGCATACATCGGCTAAAAGTCCGATGTCATAAAATCCAATCGATTTAGATAAACAGTGAAACCTTTTTTGCAATCGGCTAAATCCAACTTGTATGTAATCTTCGTAAGCCGATGAACGTCCAGATAACTTATCGGCTAGCACCTCGATAAAACACTAGCATGAACCTATCGGCTTAACAAGATTTATATTATCAACAACAATCTAGTAGGTCGGACCTAACCGATGCAACACGGATTAGATATGATAATCTAATACTCGATGAGCCAATAGATCTGTCTAATGTGATGGATATAACAAATCTATTTATAACAGCATTGCGATTGTAGAGATATATCGGCTAAGACAGAATATCAGACCTAACTAAACCGATGCGTCTCTAAACACAATGCAATTAATTAGAGATATAATTGAGATATCAGCTAGGCAAATATATCAACCAAACTGGAGCGATCCAAGAGATCGGAGCAATGCAGCCTTGAACAACACCAATGTAGCCGATGGATTCACCAGGGCCGACGGAACGTAGGACTTACCCCTTCGCTGAAGATCGGGCTGAACCAATGCAGCCCCGCGTCAGGTGCCAAATTCCGCCGGTTGATAAGTAAAACCTCAGAAAAGAGGATGACGATGCGCCGAGAGTAGTATTGATCGAGAGATAAATTGCAATGACCCTGGATGTACATATTTGTACCCATGGGTAGATATTAGTTCTTGTAGGACAAGAAAGAAACTTTCCTAAAGATAAAATGAAAACATAAAGTCTTTATTGGATACTAAATACACTTTCCTAAAGATAAAAGGAAACTAAACCCTGCCTAATTAATAGATAAACTGCCATGTCGTATCCTCCTTGAACTCGGACTCTTTTAGATAAGCTACCTTTAACTAATCTTTACCCGAATCCATCAAGAATACAAATGTTGGCATTGATAGTTTCCATCGGTCAATTCTAGGACTTTGAAGCCGATACTGACTCTAAGCCGATGATTACTTTGTGCTTACCAAATTTTGTTGTTAACATGTCGCAACCACCATCACCGGCCAGCCACCCTGATCATTGTTGTTGACTCAGCATTCGCCAGGCTGAGCAGTCCACATACATGCCGCCATCTCCATGGCACTGTCGTTGCCGCCCCTTTCTCCTAGAGCCGCCGCAGCGCTCTTCGACACACCTACTGCATCGTCGAGCAGTCGTGCTACCACCTCCTCCATCGACCATAGCCGCCTCTTCTGCTGCACCGGATCCACCCACACCAACCACCAGATACAGTCAAGCCCTCATTCCCGGATCCCATATCCATCCATGCCACTACTGTGCTGCCCAGTCCAAGGAATGGAGCGAAGGAGGAAGCCCCGCCGCTGCCCTCCCGGCGGCCACATGCACTCCAGTGCCTTGCTCCGACGGCAGCGAGGTTGGAAAATGGGTGGCAGCGGCTAGGGTTTATCTGGGGAGAAGGAAAAGGAGAGGGAGGGGGGGGGGGTCCACTTCCAGCTTAATTAGCCTAGATCTTATTGACAAATCAGTTGCTGGGTGCACAGACATGTTATTCTTTTTGCATGACCAATCTTGAACACTTAGGTATGTTAGTTGAGTGGACACTGGTCTATCTGAAACATCTCTTCACATGGAGGCTGCGAATGAGTTTTCTTTTTGAGAGACCAAAGTTTCGTTGTATGTTAAGTGATAAAGCCTTGGTAAGAAATGCTACCACAAACGAACTAATAACTCCAAACGTAAAGTGGAGGAACCCGTATGGGTGACTCGAGTGGCGACAAACTCTAGCACCTCCACCTCCTTGGACGGGCTGCGGCGGTGCTTTCGGCATCCCAGTCTTCTTGGAGGCATCATCTAGAATTAAGGTCTTGTTATTGCTTAGCATGCCTTAGGGCACGTCCAGTGTTTAGTTCGACTAAAACTTCCATGAAAGCCAAACAAAAGTTCTGTTTGACCACCACAGTGTAAAAATCGATTGTGGGACCCATGCAAAAAAATCACAATCTCAGCTGCCTATGCTCTCCTCCTGGACCTGATAGCCGTGCACAACAAATATTTTTTTAAACTGGATGTGTTCGGCTTCTCTTTAAAGATCGTTTTTTCCTCTGACACTTACCAACCGGCTTTCACAGTGTGGTCAGTTCTTTTTTTTTTTACGCAAAGTTTGATTTTAGTCAGACACGGGAGGATCTGTTAAGCAGGCTTGGAAATTTCGGACCCCTCCAATACAATATTATTTTAGCCAAAATTTCTAATTTTTTAATTTTTCATGAATTTTGGTAATATTTGTTCTAATTTAACTAAATTTTGTTCAAAATTTCGGTCTATCAGTGACCTCCGATCAAATCAGTTAAACCGAGAAAATAAACCATGCTCTTAAGAGAGTTTGGTATGGTTCAATATCAAAACTTATAGTCTTGCAATTTTTTCTACCCTTTATCTTTTTCCCTGACTATTTAGTATGGATCGTTTAAAAAAAAGAAAGCCCATTGGTGACCAAGGGCTTGTTTGATTCAAGACCATCCCTAGCCTTACCAACCTTTTGGCAATGGCAAAAATTGGTTGTTGCCAAAAATATTGGCACAAATTGGCTAAGCCTATGATTGGTTTCTACCAAAGTTGAATTTTGGCATTCAATCAAGCCAAATAATTTGGCAATAACATTTTCTTATCTATGGATATAACATATGGCAAATATTTTGGCATTACCATTTTCTTTTTGCCAAACATGTTATTCCTTTTGAATGACCAATCTTGACACCTTATGTATGTTAGTAGTGGAATCGACACTATTCTATCTAAAACATCTCTTTACATAGAGGCCGCTAATAATTTTTCTTTGAGATAACCAAATTTTCCTTACAAGTTAAGCAACAAAGCCCATTGGTAAGATATGCTACGACAAATGAACTAATAACTCCAAACGTAAAGCGGAGGATCCCGCATTTCCCACGTGGGTGACTCGAGCGGTGACAAACCCTAGTACCTCCACCCCCTTGGGTGGGTTGTGGTGGCACTTTCGGCACCGTATTTTCCTTGGACGGATCATTTAGAAAGTCCTATTATTGCCTAGTATGCCTTGACAGTTTAGGCAACACTCTTGGATGGTGGTGTCCTTTGCCCTGGTGATCTAGTAGCCCATGGATGTTTAGTTATTTGGACATGGTGTTGGATGGTGCGCTCGTGGGCCTGTTGTAGGTCTGGTGCCAACCAGTCATGCTTAGAAATAGCCGGATAGGTGCACAGTGCTAGTTCTTTACTTGGTGGTTTGTGCAGCGCTATCGACATGTGGTGGTGTGCTTTTTCTTTGTCCGGATAATAATCTCATAGGGCTATACTCTTGTTATTTTGCTGCTATATTATTATGATAACTTGGTATGGTTCGTTTTTTCTTTTTTTGGAAAAACACCTAGTTGATCAAGGGCTTGTTTGGTTCAAGTGCATTCCTAATCTTACCTTTTCTTTTTTTTTTTAATGGCAAGAATTGTTCATTGCAAAAAAAAAAAGAGATAAAAATTGGCTAGGCTTACGTTTTGGTTCTTACCAAAGTTGTACTTTGAGACCAAATATATGGCAAAATTTTGGCATAACCTTTTTTTTTTTTGCTTGGTTGAGCTTGGTACAAACCAAACAGTCACAAAATAGACTGTCATGAATCACGCCTACTAAATTCCTTTGAACCGAACTAGAATATATTTGCTCTTAAAAGATTTCTTGATTTCAATTGGTACCATTTACTAGTAGAAACTTAAATTTAAATTTTAAAAACAAAATCATAATATTGTTGTTATGGAAATTTTAGTCATTTTAGTACTTTTGTAATATATGAGTTGGGTTATACTTGAGATATCCTAAATTGCTTTAAGATGAACAATTGCTAGGTATATCAAAGATGAGCTAAAAACAATGCAGGCATTCCTTAGAGCTGCTGAAGTTATGAAAAAGAAAGATGAACTATTAAAGGTTTGGGCAGAGCAAATACGTGACCTGTCGTATGACATTGAAGATTCCCTTGATGAATTTAAAGTCCATATTGAAAGCCAAACCCTATTTCGTCAGTTGGTGAAACTTAGAGAGCGCCACCGGATCGCTATCCGTATCCACAACCTCAAATCAAGAGTTGAAGAAGTGAGTAGCAGGAACACACGCTACAATTTAGTCGAGCCTATTTCCTCCGACACAGAGGATGACATGGATTCCTATGCAGAAGACATTCGCAATCAATCAGCTCGAAATGTGGATGAAGCTGAGCTTGTTGGGTTTTCTGACTCCAAGAAAAGGCTGCTTGAAATGATCGATACCAATGCTAATGATGGTCCGGCCAAGGTAATCTGTGTTGTTGGGATGGGTGGTTTAGGCAAGACAGCTCTTTCGAGGAAGATCTTTGAAAGCGAAGAAGACATTAGGAAGAACTTCCCTTGCAATGCTTGGATTACAGTGTCACAATCATTTCACAGGATTGAGCTACTTAAAGATATGATACGCCAACTTCTTGGCCCCAGTTCTCTGGATCAACTCTTGCAAGAATTGCAAGGGAAGGTGGTGGTGCAAGTACATCATCTTTCTGAGTACCTGATAGAAGAGCTCAAGGAGAAGAGGTACTTTGTTGTTCTAGATGATCTATGGATTTTACATGATTGGAATTGGATAAATGAAATTGCATTTCCTAAGAACAATAAGAAGGGCAGTCGAATAGTAATAACCACTCGGAATGTTGATCTTGCGGAGAAGTGTGCCACAGCCTCACTGGTGTACCACCTTGATTTCTTGCAGATGAACGATGCCATAACATTGCTACTGAGAAAAACAAATAAAAATCATGAAGACATGGAATCAAATAAAAATATGCAAAAGATGGTTGAACGAATTGTAAATAAATGTGGTCGTCTACCATTAGCAATACTTACAATAGGAGCTGTGCTTGCAACTAAACAGGTGTCAGAATGGGAGAAATTCTATGAACACCTTCCTTCAGAACTAGAAATAAACCCAAGCCTGGAAGCTTTGAGTTGAATGGTGACCCTAGGTTACAACCACCTACCATCCCATCTGAAACCATGCTTTTTGTATCTAAGTATCTTTCCTGAGGATTTTGAAATCAAAAGGAATCGTCTAGTAGGTAGATGGATAGCAGAAGGGTTTGTTAGACCAAAGGTTGGGATGACGACTAAGGATGTCGGAGAAAGTTACTTTAATGAGCTAATCAACCGAAGTATGATTCAACGATCAAGAGTGGGCATAGCAGGAAAAATTAAGACTTGTCGAATTCATGATATCATCCGTGATATCACAGTTTCAATCTCGAGACAGGAAAATTTTGTATTATTACCAATGGGAGATGGCTCTGATTTAGTTCAGGAAAACACTCGCCACATAGCATTCCATGGGAGTATGTCCTGCAAAACTGGATTGGATTGGAGCATTATTCGATCATTAGCTATTTTTGGTGATAGACCCAAGAGTCTAGCACATGCAGTTTGTCCAGATCAATTGAGGATGTTACGGGTCTTGGATCTTGAAGATGTGACATTCTTAATCACTCAAAAAGATTTCGACCGTATTGCATTGTTGTGCCACTTGAAATACTTGAGTATTGGATATTCGTCATCCATATATTCACTTCCCAGATCCATTGGTAAACTACAGGGCCTACAAACTTTGAACATGCCGAGCACATACATTGCAGCACTACCAAGTGAGATCAGTAAACTCCAATGTCTGCATACTCTTCGTTGTATAGGACAGTTTCATTATGACAACTTTAGTCTAAACCACCCAATGAAGTGCATAACTAACACAATATGCCTGCCTAAAGTATTCACACCTTTAGTTAGTCGCGATGATCGTGCAAAACAAATTGCTGAATTGCACATGGCCACCAAAAGTTGCTGGTCTGAATCATTCGGTGTGAAGGTACCCAAAGGAATAGGTAAGTTGCGAGACTTGCAGGTTCTAGAGTATGTAGATATCAAGCGGACCAGTAGTAGAGCAATCAAAGAGCTGGGGCAGTTAAGCAAGTTGAGGAAATTAGGTGTGATAACAAAAGGCTCGACAAAGGAAAAATGTAAGATACTTTATGCAGCCATTGAGAAGCTCTCTTCCCTCCAATATCTCTATGTGAATGCTGCGTTATTATCAGATATTGAAACACTTGAGTGCCTAGATTCTATTTCATCTCCTCCTCCCCTACTAAGTACACTCAGGTTGAATGGAAGTCTTGAAGAGATGCCTAACTGGATTGAGCAGCTCACTCACCTGAAGAAGTTCTACTTACGGAGGAGTAAACTAAAGGAAGGTAAAACCATGCTGATACTTGGGGCATTGCCCAACCTCATGGTCCTTTATCTTTATCGGAATGCTTACCTTGGGGAGAAGCTAGTATTCAAAACGGGAGCATTCCCAAATCTTAGAACACTTTGTATTTACGAATTGGATCAGCTAAGAGAGATCAGATTTGAGGACGGCAGCTCACCCCTGTTGGAAAAGATAGAAATAGGCAAGTGCAGGTTGGAATCTGGGATTATTGGTATCATTCACCTTCCAAAGCTCAAGGAGATTCCAATTACATACGGAAGTAAAGTGGCTGGGCTTGGTCAGCTGGAGGGAGAAGTGAACGCACACCCAAATCGCCCCGTGCTGCTAATGTACAGTGACCGAAGGTATCACGACCTGGGGGCTGAAGCCGAAGGATCTTCTATAGAAGTGCAAACAGCAGATCCTGTTCCTGATGCCGAAGGATCAGTCACTGTAGCAGTGGAAGCAACGGATCCCCTTCCCGAGCAGGAGGGAGAGAGCTCGCAGTCGCAGGTGATCACGTTGACGACGAATGATAGGTCAGTCACTCCCTACATGGCAGCTTAATTAACTTGTTTCTAATTCTCTTCTTGTTCAGTATTAGCCATCAGGTGAGGGCGATGATTTCAACTCACTTTTCATCTCTCTCGTTTTCTTAACCTGACA

>Pi9-Type5

GCTTGCATATGACGTTATGTAGATAGAGATGGCCAATATAATGCGCTGGAAAGTCCAAAGTGAGGATGCAAAACATCTTATAGTGGGTAGTGGAGCCATGCAAGGACCTGGTCTAAAGCGCACCTAAACCGTCATGTGGACTGCCATTATAGTTAAAGTTAGGGGGAATATGATTCTCTTCATGTGCACCTAAACTTTAATATGCAGTGAAACGAACGCTATGATATGATGATAAGCTTAATTCCTCTCTCTGCTCAGACTGTTCAGTGCAAAAGCTACCAACGAGCTTGTCTCCTTGTGCGGTCGTGAGCTTGCTTGTGCTAAGCTTGAAGGGAGAGTCGAACGAATCCATGGCGGAGACGGTGCTGAGCATGGCGAGGTCGCTGGTGGGCAGTGCCATCAGCAAGGCCGCCTCTGCCGCTGCCAATGAGACGAGCCTCCTGCTCGGCGTCGAGAAGGACATCTGGTACGTACTGCACTGCGCTCTCGTTTATCCTAGCTCGGTTGTATCGACTTCCAGCTTAATCTTTTTAATAATGAATAAAAACCCGGACTTGTTATCCATAAGTGGATATACACAGTCAAAACACGCGACAAGTTCTTAGGCTCTTAATTAATCTCGAAATTGAGGAACACCATGAAACACTAAAAGAGAGCTCGAAGACTAGGAAAGAAAACTAGAAGACTAAGCTTTGAAAGTCTTCTAAATCCAAGCATCTCGACATTGATCATCCTTGTGCAACATCAACCCTTCCTATTGCTTCACCAGAATCGGCGTCCCTTGTGGAGATCTCTGTTGTAACGTCAAGGGGAAAATCGGAGAAGCAGAACTAGTCCGCGCTGCCTTCGCTACGCCATCTCCGCCTTAGAGGATCTCATCCACGAAACATCCACCATCCAAACGGGAAACAGTTTTAAACACTCGTGGACGTTCACCCGTTCATCTAAATGGTTATGAAAAATTTTCAAAAAAAATAACATGATAGGTTAACATGTAATATATCATCTTATAAATATGCAAGTTCAAATTTGATTTCTACAAGTTGTAACAAAAATAACAAATTTTACTGTGAATATACGTAAACTAGTTAAAGTTTAATTTGTTATTTTTGTTACAACTTGTAGAAGTCGAATTTAAATCTGTATGTTTGTGAAATGAGATATTACATATTAACCTATCTTATAATTTTTTTTAGAAATTTTTTAGAATTATTTAGGTGGCATACAAGAAACGGATGGACATCCACAAAGAGATTAGTATCCATCTCCACATCCAAACCCGTTGTTGCACCATCTGTCGAATCTGTCGAATCCGGCTGTGGACGCTCGGAGGCAAGAGCTAGCTCACCCGTCCCACACACACACCCAACGACGTCACAAGCGCCTCCGAACAACGCCAACTGATAACTTGGCAGCTCCTACGTGCCGACGTCGCGGTACTTGCCGGCGCTCCTAGCGCATGCACCGTCGAACCACACCGTCACCGACCAGCTACCCACCGCCGCCGACTTCTGCCTCATCTGCCATCGTCGCCCTAGCCCAAGTTATCATCGTGGCAATTGCCGAGGCTCCTAAGTGTGCCACGGCCGAGGCAAAGTTCTAACTGAATCAGACAGCCACCACCGACACTTCTGCTTCATCTGCCATCGCCGTACTAGTTCAAGTTGTCGCTGTGGCAATCACTGTTGTTATTGCCGCGCCCTGACCCCTATCGTCGTCGCTCTTAGCGCGTCGTCGAGCCGACCAGCCACTGTCGTGCAGATGAAAAAAAAAAACACTTTTTGGCCTGAGAGATCTGCTTAGTTCCATTGCAGGTCCAACATGCTGTGAGATGCGGGCGTGCCAGTCAGTTTGATCTTGCAACTGACAAGATATATAAACAGCAGATAAAACAGCCTATCGACTAACAAGCCGATGGAGTAATTCCAGCCGATAGCCGATATTAGCCGATGCCGATTCTAGCCGATGTCGATAGGGTTTTGAACTATCGGCTATATGTCCAATGTAGGCAATGATATAAAGACAATTGGCTGATGATAATAAAATATAAAAATATAATCCAATAGAAACCAATCGGCTAATAATAAGTATTGATCCGATAGTTAAAGCATACATCGGCTAAAAGTCCGATGTCATAAAATCCAATCGATTTAGATAAACAGTGAAACCTTTGTTGCAATCGGCTAAATCCAACTTGTATGTAATCTTCGTAAGCCGATGAACGTCCAGATAACTTATCGGCTAGCACCTCGATAAAACACTAGCATGAACCTATCGGCTTAACAAGATTTATATTATCAACAACAATCTAGTAGGTCGGACCTAACCGATGCAACACGTATTAGATATGATAATCTAATACTCGATGAGCCAATAGATCTGTCTAATGTGATGGATATAACAAATCTATTTATAAAAGCATTGCGATTGTAGAGATATATCGGCTAAGACAGAATATCAGACCTAACTAAACCGATGCGTCTCTAAACACAATGCAATTAATTAGAGATATAATTGAGATATCAGCTAGGCAAATATATCAACCAAACTAGAGCGATCCAAGAGATCGGAGCAATGCAGCCTTGAACAACACCAATGTAGCCGATGGATTCACCAGGGTCGACGGAATGTAGGACTTACCCCTTCCCTGAAGATCGGGCTGAACCAATGCAGTCCCATGTCAGGTGCCAAATTCCGCCGGTTGATAAGTAAAACCTCAGAAAAGAGGATGACGATGCGCCGAGAGTAGTATTGATCGAGAGATAAATTGCAATGACCCTGGATGTACATATTTGTACCCATGGGTAGATATTAGTTCTTGTAGGACAAGAAAGAAACTTTCCTAAAGATAAAATGAAAACATAAAGTTTTTATTGGATACTAAACACACTTTCCTAAAGATAAAAGGAAACTAAACCCTGCCTAATTAATAGATAAACTGCCATGTCGTATCCTCCTTGAACTCGAACTCTTTTAGATAAGCTTCCTTTAACTAATCTTTACCCGAATCCATCAAGAATACAAATGTTGGCATTGATAGTTTTCATCGGTCAATTCTAGGACTTTGAAGCCGATACTGACTCTAAGCCGATGACTACTTTGGGCTTACCAAATTTTGTTGTTAACATGTCGCGACCACCATCACCGGCCAGCCACCCTGATCATTGTTGTTGACTCAGCATTCGCCAGGCTGAGCAGTCCACATACATGCCGCCATCTCCATGGCACTGTCGTTGCCGCCCCTTTCTCCTAGAGCCGCCGCAGCGCTCTTCGACACACCTACTGCATCGTCGAGCAGTCGTGCTACCACCTCCTCCATCGACCATAGCCGCCTCTTCTGCTGCACCGGATCCACCCACACCAACCACCAGATACAGTCAAGCCCTCATTCCCGGATCCCATATCCATCCATGCCACTACTGTGCTGCCCAGTCCAAGGAATGGAGCGAAGGAGGAAGCCCCGCCGCTGCCCTCCCGGCGGCCACATGCACTCCAGTGCCTTGCTCCGACGGCAGCGAGGTTGGAAAATGGGTGGCAGCGGCTAGGGTTTATCTGGGGAGAAGGAAAAGGAGAGGGAGGGGGGGGGGGAGGGAGGGTCCACTTCCAGCTTAATTAGCCTAGATCTTATTGACAAATCAGTTGCTGGGTGCACAAACATGTTATTTTTTTTGCATGACCAATCTTGAACACTTAGGTATGTTAGTTGAGTGGACACTGGTCTATCTGAAACATCTCTTCACATGGAGGCTGCGAATGAGTTTTCTTTTTGAGAGACCAAAGGTTCGTTGTATGTTAAGTGATAAAGCCTTGGTAAGAAATGCTACCACAAACGAACTAATAACTCCAAACGTAAAGTGGAGGAACCCGTATGGGTGACTCGAGTGGCGACAAACTCTAGCACCTCCACCTCCTTGGACGGGCTGCGGCGGTGCTTTCGGCATCCCAGTCTTCTTGGAGGCATCATCTAGAATTAAGGTCTTGTTATTGCTTAGCATGCCTTAGGGCACGTCCAGTGTTTAGTTCGACTAAAACTTCCATGAAAGCCAAACAAAAGTTCTGTTTGACCACCACAGTGTAAAAATCGATTGTGGGACCCATGCAAAAAAATCACAATCTCAGCTGCCTATGCTCTCCTCCTGGACCTGATAGCCGTGCACAACAAATATTTTTTTAAACTGGATGTGTTCGGCTTCTCTTTAAAGATCGTTTTTTCCTCTGACACTTACCAACCGGCTTTCACAGTGTGGTCAGTTCTTTTTTTTTTTACGCAAAGTTTGATTTTAGTCAGACACGGGAGGATCTGTTAAGCAGGCTTGGAAATTTCGGACCCCTCCAATACAATATTATTTTAGCCAAAATTTCTAATTTTTTAATTTTTCATGAATTTTGGTAATATTTGTTCTAATTTAACTAAATTTTGTTCAAAATTTCGGTCTATCAGTGACCTCCGATCAAATCAGTTAAACCGAGAAAATAAACCATGCTCTTAAGAGAGTTTGGTATGGTTCAATATCAAAACTTATAGTCTTGCAATTTTTTCTACCCTTTATCTTTTTCCCTGACTATTTAGTATGGATCGTTTAAAAAAAAGAAAGCCCATTGGTGACCAAGGGCTTGTTTGATTCAAGACCATCCCTAGCCTTACCAACCTTTTGGCAATGGCAAAAATTGGTTGTTGCCAAAAATATTGGCACAAATTGGCTAAGCCTATGATTGGTTTCTACCAAAGTTGAATTTTGGCATTCAATCAAGCCAAATAATTTGGCAATAACATTTTCTTATCTATGGATATAACATATGGCAAATATTTTGGCATTACCATTTTCTTTTTGCCAAACATGTTATTCCTTTTGAATGACCAATCTTGACACCTTATGTATGTTAGTAGTGGAATCGACACTATTCTATCTAAAACATCTCTTTACATAGAGGCCGCTAATAATTTTTCTTTGAGATAACCAAATTTTCCTTACAAGTTAAGCAACAAAGCCCATTGGTAAGATATGCTACGACAAATGAACTAATAACTCCAAACATAAAGCGGAGGATCCCGCATTTCCCACGTGGGTGACTCGAGCGGTGACAAACCCTAGTACCTCCACCCCCTTGGGTGGGTTGTGGTGGCACTTTCGGCACCGTATTTTCCTTGGACGGATCATTTAGAAAGTCCTATTATTGCCTAGTATGCCTTGACAGTTTAGGCAACACTCTTGGATGGTGGTGTCCTTTGCCCTGGTGATCTAGTAGCCCATGGATGTTTAGTTATTTGGACATGGTGTTGGATGGTGCGCTCGTGGGCCTGTTGTAGGTCTGGTGCCAACCAGTCATGCTTAGAAATAGCCGGATAGGTGCACAGTGCTAGTTCTTTACTTGGTGGTTTGTGCAGCGCTATCGACATGTGGTGGTGTGCTTTTTCTTTGTCCGGATAATAATCTCATAGGGCTATACTCTTGTTATTTTGCTGCTATATTATTATGATAACTTGGTATGGTTCGTTTTTTCTTTTTTTGGAAAAACACCTAGTTGATCAAGGGCTTGTTTGGTTCAAGTGCATTCCTAATCTTACCTTTTCTTTTTTTTTTCAATGGCAAGAATTGTTCATTGCAAAAAAAAAAAGAGATAAAAATTGGCTAGGCTTACGTTTTGGTTCTTACCAAAGTTGTACTTTGAGACCAAATATATGGCAAAATTTTGGCATAACCTTTTTTTTTTTTGCTTGGTTGAGCTTGGTACAAACCAATCAGTCACAAAATAGACTGTCATGAATCACGCCTACTAAATTCCTTTGAACCGAACTAGAATATATTTGCTCTTAAAAGATTTCTTGATTTCAATTGGTACCATTTACTAGTAGAAACTTAAATTTAAATTTTAAAAACAAAATCATAATATTGTTGTTATGGAAATTTTAGTCATTTTAGTAATTTTGTAATATATGAGTTGGGTTATACTTGAGATATCCTAAATTGCTTTAAGATGAACAATTGCTAGGTATATCAAAGATGAGCTAAAAACAATGCAGGCATTCCTTAGAGCTGCTGAAGTTATGAAAAAGAAAGATGAACTATTAAAGGTTTGGGCAGAGCAAATACGTGACCTGTCGTATGACATTGAAGATTCCCTTGATGAATTTAAAGTCCATATTGAAAGCCAAACCCTATTTCGTCAGTTGGTGAAACTTAGAGAGCGCCACCGGATCGCTATCCGTATCCACAACCTCAAATCAAGAGTTGAAGAAGTGAGTAGCAGGAACACACGCTACAATTTAGTCGAGCCTATTTCCTCCGGCACAGAGGATGACATGGATTCCTATGCAGAAGACATTCGCAATCAATCAGCTCGAAATGTGGATGAAGCTGAGCTTGTTGGGTTTTCTGACTCCAAGAAAAGGCTGCTTGAAATGATCGATACCAATGCTAATGATGGTCCGGCCAAGGTAATCTGTGTTGTTGGGATGGGTGGTTTAGGCAAGACAGCTCTTTCGAGGAAGATCTTTGAAAGCGAAGAAGACATTAGGAAGAACTTCCCTTGCAATGCTTGGATTACAGTGTCACAATCATTTCACAGGATTGAGCTACTTAAAGATATGATACGCCAACTTCTTGGTCCCAGTTCTCTGGATCAACTCTTGCATGAATTGCAAGGGAAGGTGGTGGTGCAAGTACATCATCTTTCTGAGTACCTGATAGAAGAGCTCAAGGAGAAGAGGTACTTTGTTGTTCTAGATGATCTATGGATTTTACATGATTGGAATTGGATAAATGAAATTGCATTTCCTAAGAACAATAAGAAGGGCAGTCGAATAGTAATAACCACTCGGAATGTTGATCTAGCGGAGAAGTGTGCCACAGCCTCACTGGTGTACCACCTTGATTTCTTGCAGATGAACGATGCCATTTCATTGCTACTGAGAAAAACAAATAAAAATCATGAAGACATGGAATCAAATAAAAATATGCAAAAGATGGTTGAACGAATTGTAAATAAATGTGGTCGTCTACCATTAGCAATACTTACAATAGGAGCTGTGCTTGCAACTAAACAGGTGTCAGAATGGGAGAAATTCTATGAACAACTTCCTTCAGAACTAGAAATAAACCCAAGCCTGGAAGCTTTGAGGAGAATGGTGACCCTAGGTTACAACCACCTACCATCCCATCTGAAACCATGCTTTTTGTATCTAAGTATCTTTCCTGAGGATTTTGAAATACAAAGGAATCGTCTAGTAGGTAGATGGATAGCAGAAGGGTTTGTTAGACCAAAGGTTGGGATGACGACTAAGGATGTCGGAGAAAGTTACTTTAATGAGCTAATCAACCGAAGTATGATTCAACGATCAAGAGTGGGCACAGCAGGAAAAATTAAGACTTGTCGAATCCATGATATCATCCGTGATATCACAGTTTCAATCTCGAGACAGGAAAATTTTGTATTATTACCAATGGGAGATGGCTCTGATTTAGTTCAGGAAAACACTCGCCACATAGCATTCCATGGGAGTATGTCCTGCAAAACAGGATTGGATTGGAGCATTATTCGATCATTAGCTATTTTTGGTGACAGACCCAAGAGTCTAGCACATGCAGTTTGTCCAGATCAATTGAGGATGTTACGGGTCTTGGATCTTGAAGATGTGACATTCTTAATCACTCAAAAAGATTTCGACCGTATTGCATTGTTGTGCCACTTGAAATACTTGAGTATTGGATATTCGTCATCCATATATTCACTTCCCAGATCCATTGGTAAACTACAGGGCCTACAGACTTTGAACATGTCAAGCACATACATTGCAGCACTACCAAGTGAGATCAGTAAACTCCAATGTCTGCATACTCTTCGTTGTATAAGAGAGCTTGAATTTGACAACTTTAGTCTAAATCACCCAATGAAGTGCATAACTAACACAATATGCCTGCCTAAAGTATTCACACCTTTAGTTAGTCGCGATAATCGTGCAAAACAAATTGCTGAATTTCACATGGCCACCAAAAGTTTCTGGTCTGAATCATTCGGTGTGAAGGTACCCAAAGGAATAGGTAAGTTGCGAGACTTACAGGTTCTAGAGTATGTAGATATCAGGCGGACCAGTAGTAGAGCAATCAAAGAGCTGGGGCAGTTAAGCAAGTTGAGGAAATTAGCTGTGATAACAAAAGGCTCGACAAAGGAAAAATGTAAGATACTTTATGCAGCCATTGAGAAGCTCTCTTCCCTCCAATCTCTCTATATGAATGCTGCGTTATTATCAGATATTGAAACACTTGAGTGCCTAGATTCTATTTCATCTCCTCCTCCCCTACTGAGGACACTCGGGTTGAATGGAAGTCTTGAAGAGATGCCTAACTGGATTGAGCAGCTCACTCACCTGAAGAAGTTCAACTTATGGAGTAGTAAACTAAAGGAAGGTAAAAACATGCTGATACTTGGGGCACTGCCCAACCTCATGTTCCTTTCTCTTTATCATAATTCTTATCTTGGGGAGAAGCTAGTATTCAAAACGGGAGCATTCCCAAATCTTAGAACACTTGTGATTTTCAATTTGGATCAGCTAAGAGAGATCAGATTTGAGGACGGCAGCTCACCCCAGTTGGAAAAGATAGAAATCTCTTGCTGCAGGTTGGAATCAGGGATTATTGGTATCATTCACCTTCCAAGGCTCAAGGAGATTTCACTTGAATACAAAAGTAAAGTGGCTAGGCTTGGTCAGCTGAAGGGAGAAGTGAACACACACCCAAATCGCCCCGTGCTGCGAATGGACAGTGACCGAAGGGATCACGACCTGGGGGCTGAAGCCGAAGGATCTTCTATAGAAGTGCAAACAGCAGATCCTGTTCCTGATGCCCAAGGATCAGTCACTGTAGCAGTGGAAGCAACGGATCCCCTTCCCGAGCAGGAGGGAGAGAGCTCGCAGTCGCAGGTGATCACGTTGACGACGAATGATAGGTCAGTCACTCCCTACATGGCAGCTTAATTAACTTGTTTCTAATTCTCTTCTTGTTCAGTATTAGCCATCAGGTGAGGGCGATGATTTCAACTCACTTTTCATCTCTCTCGTTTTCTTAACCTGACA

>Pi9-Type6

GCTTGCATATGACGTCATGTAGATAGAGATGGCCAATATAATGCGCTGGAAAGTCCAAAGTGAGGATGCAAAACATCTTATAGTGGGTAGTGGAGCCATGCAAGGACCTGGTCTAAAGCGCACCTAAACCGTAATGTGGACTGCCATTATAGTTAAAGTTAGGGGGAATATGATTCTCTTCATGTGCACCTAAACCGTAATATGCAGTGAAACGAACGCTATGATATGATGATAAGCTTAATTCCTCTCTCTGCTCAGACTGTTCAGTGCAAAAGCTACCAACGAGCTTGTCTCCTTGTGCGGTCGTGAGCTTGCTTGTGCTAAGCTTGAAGGGAGAGTCGAACGAATCCATGGCGGAGACGGTGCTGAGCATGGCGAGGTCGCTGGTGGGCAGTGCCATCAGCAAGGCCGCCTCTGCCGCTGCCAATGAGACGAGCCTCCTGCTCGGCGTCGAGAAGGACATCTGGTACGTACTGCACTGCTCTCGTTTATCCTAGCAAGTTCTTAGGCTCTTAATCTCGAAATTGAGGAACACCATGAAACACTAAAAGAGAGCTCGAAGACTAGGAAAGAAAACTAGAAGACTAAGCTTTGAAAGTCTTCTAAATCCAAGCATCTCGACATTGATCATCCTTGTGCAACATCATCCCTTCCTATTGCTTCACCAGAATCGGTGTCCCTTGTGGAGATCTCTGTCGTAGCGTCAAGGGGAGAATCCGAGAAGCAGAACTAGTCCGCGCTGCCTTCGCTACGCCATCTCCGCCATAGAGGATCTCATCCACGAAACATCCACCATCCAAACGGGAAACTGTTTTAAACACTCGGGTGGATGTTCACCCGTTTCTTGCATGTCATCTAAATGGTTATGAAAAATTTTCAAAAAAAAAACATGATAGGTTAATATATAATATATCATCTCACAAATATGCAAGTTCAAATTCAACTTTTATAAGTTGTAAGTATAACAGGACGTTCATCTCACAAATATGCAAGTTTAAATTTAACTTTTACAAGTTGTAAGTGTAACAGTACGTCCATCGGATAGATTAATATCCATCTCCCCATCCAAACCCGTTGTTGCACCATCTGTCGAATCCGGCTGTGGACGCTCGGAGGCAAGAGCTAGCTCACCCGTCCCACACACACACCCAACGACGTCACAAGCGCCTCCGAACAACGCCAACTGATAACTTGGCAGCTCCTACGTGCCGACGTCGCGGTATTTGCCGGCGCTCCTAGCGCACGCACCGTCGAACCACACCGTCACCGACCAACTACCCACCGCCGCCGACTTCTGCCTCATCTGCCATCGTCGCCCTAGCCCAAGTTATCATCGTGGCAATTGCCGAGGCTCCTAAGTGTGCCACGGCCGAGGCAAAGTTCTAACTGAATCAGACAGCCACCACCGACACTTCTGCTTCATCTGCCATCGCCGTACTAGTTCAAGTTGTCGCTGTGGCAATCATTGGCCCTCCTAGCGTGCCACCCAACCGGACAACCACGACATCCCCCATCACTGTTGTTATTGCCGCGCCCTGACCCCTATCGTCGTCGCTCTTAGCGCGTCGACGAGCCGACCAGCCACTGTCGTGCAGATGAAAAAAAAAACACATATTGGCCTGAGAGATCTGCTTAGTTCCAGTGCAGGTCCAACATGCTGTGAGATGCGGGCGTGCCAGTCAGTTTGATCTTGCAACTGACAAGATATATAAACAGCAGATAAAACAGCCGATCGACTAACAAGCCGATGGAGTAATTCCAGCCGATAGCCGATATTAGCCGATGCCGATTCTAGCCGATGTCGATAGGGTTTTGAACTATCGGCTATATGTCTAATGTAGGCAATGATATAAAGACAATTGGCTGATGATAATAAAATATAAAAATATAATCCAATAGAAACCAATCGGCTAATAATAAATATTGATCCGATGGTTAAAGCATACATCGGCTAAAAGTCCGATGTCATAAAATCCAATCGATTTAGATAAACAGTGAAACCTTTTTTGCAATCGGCTAAATCCAACTTGTATGTAATCTTCGTAAGCCGATGAACGTCCAGATAACTTATCGGTTAGCACCTCGATAAAACACTAGCATGAACCTATCGGCTTAACAAGATTTATATTATCAACAACAATCTAGTAGGTCGGACCTAACCGATGCAACACGGATTAGATATGATAATCTAATACTCGATGAGCCAATAGATCTGTCTAATGTGATGGATATAACAAATCTATTTATAACAGCATTGCGATTGTAGAGATATATCGGCTAAGACAGAATATCAGACCTAACTAAACCGATGCGTCTCTAAACACAATGCAATTAATTAGAGATATAATTGAGATATCAGCTAGGCAAATATATCAACCAAACTGGAGCGATCCAAGAGATCGGAGCAATGCAGCCTTGAACAACACCAATGTAGCCGATGGATTCACCAGGGCCGACGGAACGTAGGACTTACCCCTTCGCTGAAGATCGGGCTGAACCAATGCAGCCCCGCGTCAGGTGCCAAATTCCGCCGGTTGATAAGTAAAACCTCAGAAAAGAGGATGACGATGCGCCGAGAGTAGTATTGATCGAGAGATAAATTGCAATGACCCTGGATGTACATATTTGTACCCATGGGTAGATATTAGTTCTTGTAGGACAAGAAAGAAACTTTCCTAAAGATAAAATGAAAACATAAAGTCTTTATTGGATACTAAATACACTTTCCTAAAGATAAAAGGAAACTAAACCCTGCCTAATTAATAGATAAACTGCCATGTCGTATCCTCCTTGAACTCGGACTCTTTTAGATAAGCTACCTTTAACTAATCTTTACCCGAATCCATCAAGAATACAAATGTTGGCATTGATAGTTTCCATCGGTCAATTCTAGGACTTTGAAGCCGATACTGACTCTAAGCCGATGATTACTTTGTGCTTACCAAATTTTGTTGTTAACATGTCGCAACCACCATCACCGGCCAGCCACCCTGATCATTGTTGTTGACTCAGCATTCGCCAGGCTGAGCAGTCCACATACATGCCGCCATCTCCATGGCACTGTCGTTGCCGCCCCTTTCTCCTAGAGCCGCCGCAGCGCTCTTCGACACACCTACTGCATCGTCGAGCAGTCGTGCTACCACCTCCTCCATCGACCATAGCCGCCTCTTCTGCTGCACCGGATCCACCCACACCAACCACCAGATACAGTCAAGCCCTCATTCCCGGATCCCATATCCATCCATGCCACTACTGTGCTGCCCAGTCCAAGGAATGGAGCGAAGGAGGAAGCCCCGCCGCTGCCCTCCCGGCGGCCACATGCACTCCAGTGCCTTGCTCCGACGGCAGCGAGGTTGGAAAATGGGTGGCAGCGGCTAGGGTTTATCTGGGGAGAAGGAAAAGGAGAGGGAGGGGGGGGGGGGGTCCACTTCCAGCTTAATTAGCCTAGATCTTATTGACAAATCAGTTGCTGGGTGCACAGACATGTTATTCTTTTTGCATGACCAATCTTGAACACTTAGGTATGTTAGTTGAGTGGACACTGGTCTATCTGAAACATCTCTTCACATGGAGGCTGCGAATGAGTTTTCTTTTTGAGAGACCAAAGTTTCGTTGTATGTTAAGTGATAAAGCCTTGGTAAGAAATGCTACCACAAACGAACTAATAACTCCAAACGTAAAGTGGAGGAACCCGTATGGGTGACTCGAGTGGCGACAAACTCTAGCACCTCCACCTCCTTGGACGGGCTGCGGCGGTGCTTTCGGCATCCCAGTCTTCTTGGAGGCATCATCTAGAATTAAGGTCTTGTTATTGCTTAGCATGCCTTAGGGCACGTCCAGTGTTTAGTTCGACTAAAACTTCCATGAAAGCCAAACAAAAGTTCTGTTTGACCACCACAGTGTAAAAATCGATTGTGGGACCCATGCAAAAAAATCACAATCTCAGCTGCCTATGCTCTCCTCCTGGACCTGATAGCCGTGCACAACAAATATTTTTTTAAACTGGATGTGTTCGGCTTCTCTTTAAAGATCGTTTTTTCCTCTGACACTTACCAACCGGCTTTCACAGTGTGGTCAGTTCTTTTTTTTTTTACGCAAAGTTTGATTTTAGTCAGACACGGGAGGATCTGTTAAGCAGGCTTGGAAATTTCGGACCCCTCCAATACAATATTATTTTAGCCAAAATTTCTAATTTTTTAATTTTTCATGAATTTTGGTAATATTTGTTCTAATTTAACTAAATTTTGTTCAAAATTTCGGTCTATCAGTGACCTCCGATCAAATCAGTTAAACCGAGAAAATAAACCATGCTCTTAAGAGAGTTTGGTATGGTTCAATATCAAAACTTATAGTCTTGCAATTTTTTCTACCCTTTATCTTTTTCCCTGACTATTTAGTATGGATCGTTTAAAAAAAAGAAAGCCCATTGGTGACCAAGGGCTTGTTTGATTCAAGACCATCCCTAGCCTTACCAACCTTTTGGCAATGGCAAAAATTGGTTGTTGCCAAAAATATTGGCACAAATTGGCTAAGCCTATGATTGGTTTCTACCAAAGTTGAATTTTGGCATTCAATCAAGCCAAATAATTTGGCAATAACATTTTCTTATCTATGGATATAACATATGGCAAATATTTTGGCATTACCATTTTCTTTTTGCCAAACATGTTATTCCTTTTGAATGACCAATCTTGACACCTTATGTATGTTAGTAGTGGAATCGACACTATTCTATCTAAAACATCTCTTTACATAGAGGCCGCTAATAATTTTTCTTTGAGATAACCAAATTTTCCTTACAAGTTAAGCAACAAAGCCCATTGGTAAGATATGCTACGACAAATGAACTAATAACTCCAAACGTAAAGCGGAGGATCCCGCATTTCCCACGTGGGTGACTCGAGCGGTGACAAACCCTAGTACCTCCACCCCCTTGGGTGGGTTGTGGTGGCACTTTCGGCACCGTATTTTCCTTGGACGGATCATTTAGAAAGTCCTATTATTGCCTAGTATGCCTTGACAGTTTAGGCAACACTCTTGGATGGTGGTGTCCTTTGCCCTGGTGATCTAGTAGCCCATGGATGTTTAGTTATTTGGACATGGTGTTGGATGGTGCGCTCGTGGGCCTGTTGTAGGTCTGGTGCCAACCAGTCATGCTTAGAAATAGCCGGATAGGTGCACAGTGCTAGTTCTTTACTTGGTGGTTTGTGCAGCGCTATCGACATGTGGTGGTGTGCTTTTTCTTTGTCCGGATAATAATCTCATAGGGCTATACTCTTGTTATTTTGCTGCTATATTATTATGATAACTTGGTATGGTTCGTTTTTTCTTTTTTTGGAAAAACACCTAGTTGATCAAGGGCTTGTTTGGTTCAAGTGCATTCCTAATCTTACCTTTTCTTTTTTTTTTCAATGGCAAGAATTGTTCATTGCAAAAAAAAAAAGAGATAAAAATTGGCTAGGCTTACGTTTTGGTTCTTACCAAAGTTGTACTTTGAGACCAAATATATGGCAAAATTTTGGCATAACCTTTTTTTTTTTTGCTTGGTTGAGCTTGGTACAAACCAATCAGTCACAAAATAGACTGTCATGAATCACGCCTACTAAATTCCTTTGAACCGAACTAGAATATATTTGCTCTTAAAAGATTTCTTGATTTCAATTGGTACCATTTACTAGTAGAAACTTAAATTTAAATTTTAAAAACAAAATCATAATATTGTTGTTATGGAAATTTTAGTCATTTTAGTACTTTTGTAATATATGAGTTGGGTTATACTTGAGATATCCTAAATTGCTTTAAGATGAACAATTGCTAGGTATATCAAAGATGAGCTAAAAACAATGCAGGCATTCCTTAGAGCTGCTGAAGTTATGAAAAAGAAAGATGAACTATTAAAGGTTTGGGCAGAGCAAATACGTGACCTGTCGTATGACATTGAAGATTCCCTTGATGAATTTAAAGTCCATATTGAAAGCCAAACCCTATTTCGTCAGTTGGTGAAACTTAGAGAGCGCCACCGGATCGCTATCCGTATCCACAACCTCAAATCAAGAGTTGAAGAAGTGAGTAGCAGGAACACACGCTACAATTTAGTCGAGCCTATTTCCTCCGGCACAGAGGATGACATGGATTCCTATGCAGAAGACATTCGCAATCAATCAGCTCGAAATGTGGATGAAGCTGAGCTTGTTGGGTTTTCTGACTCCAAGAAAAGGCTGCTTGAAATGATCGATACCAATGCTAATGATGGTCCGGCCAAGGTAATCTGTGTTGTTGGGATGGGTGGTTTAGGCAAGACAGCTCTTTTGAGGAAGATCTTTGAAAGCGAAGAAGACATTAGGAAGAACTTCCCTTGCATTGCTTGGATTACAGTGTCACAATCATTTCACAGGATTGAGCTACTTAAAGATATGATACGCCAACTTCTTGGCCCCAGTTCTCTGGATCAACTCTTGCAAGAATTGCAAGGGAAGGTGGTGGTGCAAGTACATCATCTTTCTGAGTACCTGATAGAAGAGCTCAAGGAGAAGAGGTACTTTGTTATTCTAGATGATCTATGGATTTTACATGATTGGAATTGGATAAATGAAATTGCATTTCCTAAGAACAATAAGAAGGGCAGTCGAATAGTAATAACCACTCGGAATGTTGATCTAGCGGAGAAGTGTGCCACAGCCTCACTGGTGTACCACCTTGATTTCTTGCAGATGAACGATGCCATAACATTGCTACTGAGAAAAACAAATAAAAATCATGAAGACATGGAATCAAATAAAAATATGCAAAAGATGGTTGAACGAATTGTAAATAAATGTGGTCGTCTACCATTAGCAATACTTACAATAGGAGCTGTGCTTGCAACTAAACATGTGTCAGAATGGGAGAAATTCTATGAACAACTTCCTTCAGAACTAGAAATAAACCCAAGCCTGGAAGCTTTGAGGAGAATGGTGACCCTAGGTTACAACCACCTACCATCCCATCTGAAACCATGCTTTTTGTATCTAAGTATCTTTCCTGAGGATTTTGAAATCAAAAGGAATCGTCTAGTAGGTAGATGGATAGCAGAAGGGTTTGTTAGACCAAAGGTTGGGATGACGACTAAGGATGTCGGAGAAAGTTACTTTAATGAGCTAATCAACCGAAGTATGATTCAACGATCAAGAGTGGGCATAGCAGGAAAAATTAAGACTTGTCGAATCCATGATATCATCCGTGATATCACAGTTTCAATCTCGAGACAGGAAAATTTTGTATTATTACCAATGGGAGATGGCTCTGATTTAGTTCAGGAAAACACTCGCCACATAGCATTCCATGGGAGTATGTCCTGCAAAACAGGATTGGATTGGAGCATTATTCGATCATTAGCTATTTTTGGTGACAGACCCAAGAGTCTAGCACATGCAGTTTGTCCAGATCAATTGAGGATGTTACGGGTCTTGGATCTTGAAGATGTGACATTCTTAATCACTCAAAAAGATTTCGACCGTATTGCATTGTTGTGCCACTTGAAATACTTGAGTATTGGATATTCGTCATCCATATATTCACTTCCCAGATCCATTGGTAAACTACAGGGCCTACAAACTTTGAACATGCCGAGCACATACATTGCAGCACTACCAAGTGAGATCAGTAAACTCCAATGTCTGCATACTCTTCGTTGTAGTAGAAAGTTTGTTTATGACAACTTTAGTCTAAACCACCCAATGAAGTGCATAACTAACACAATATGCCTGCCTAAAGTATTCACACCTTTAGTTAGTCGCGATGATCGTGCAATACAAATTGCTGAATTGCACATGGCCACCAAAAGTTGCTGGTCTGAATCATTCGGTGTGAAGGTACCCAAAGGAATAGGTAAGTTGCGAGACTTACAGGTTCTAGAGTATGTAGATATCAGGCGGACCAGTAGTAGAGCAATCAAAGAGCTGGGGCAGTTAAGCAAGCTGAGGAAATTAGGTGTGACAACAAACGGGTCGACAAAGGAAAAATGTAAGATACTTTATGCAGCCATTGAGAAGCTCTCTTCCCTCCAATCTCTCCATGTGGATGCTGTGTTATTCTCAGGTATTATTGGAACACTTGAGTGCCTAGATTCTATTTCATCTCCTCCTCCCCTACTGAGGACACTCGGGTTGAATGGAATTCTTGAAGAGATGCCTAACTGGATTGAGCAGCTCACTCACCTGAAGAAGTTCTACTTATTAAGCAGCAAACTAAAGGAAGGTAAAACCATGCTGATACTTGGGGCATTGCCCAACCTCATGGTCCTTTATCTTTATTGGAATGCTTACCTTGGGGAGAAGCTAGTATTCAAAACGGGAGCATTCCCAAATCTTAGAACACTTCATATTTACGAATCGGATCAGCTAAGAGAGATGAGATTTGAGGATGGCAGCTCACCCCTGTTGGAAAAGATAGAAATATTCAGGTGCAGGTTGGAATCAGGGATTATTGGTATCATTCACCTTCCAAGGCTCAAGGAGATTTCACTTGAATACAAAAGTAAAGTGGCTAGGCTTGGTCAGCTGGAGGGAGAAGTGAGCACACACCCAAATCGCCCCGTGCTGCGAATGGACAGTGACCGAAGGGATCACGACCTGGGGGCTGAAGCCGAAGGATCTTCTATAGAAGTGCAAACAGCAGATCCTGTTCCTGATGCCCAAGGATCAGTCACTGTAGCAGTGGAAGCAACGGATCCCCTTCCCGAGCAGGAGGGAGAGAGCTCGCAGTCGCAGGTGATCATGTTGACGACGAACGATAGGTCAGTCACTCCCTACATGGCAGCTTAATTAACTTGTTTCTAATTCTCTTCTTGTTCAGTATTAGCCATCAGGTGAGGGCGATGATTTCAACTCACTTTTCATCTCTCTCGTTTTCTTAACCTGACA

>Pi9-Type7

GCTTGCATATGACGTTATGTAGATAGAGATGGCCAATATAATGCGCTGGAAAGTCCAAAGTGAGGATGCAAAACATCTTATAGTGGGTAGTGGAGCCATGCAAGGACCTGGTCTAAAGCGCACCTAAACCGTAATGTGGACTTTAAAGTTAGGGGGAATATGATTCTCTTCATGTGCACCTAAACCGTAATATGCAGTGAAACGAACGCTATGATATGATGATAAGCTTAATTCCTCTCTCTGCTCAGACTGTTCAGTGCAAAAGCTACCAACGAGCTTGTCTCCTTGTGCGGTCGTGAGCTTGCTTGTGCTAAGCTTGAAGGGAGAGTCGAACGAATCCATGGCGGAGACGGTGCTGAGCATGGCGAGGTCGCTGGTGGGCAGTGCCATCAGCAAGGCCGCCTCTGCCGCTGCCAATGAGACGAGCCTCCTGCTCGGCGTCGAGAAGGACATCTGGTACGTACTGCACTGCTCTCGTTTATCCTAGCAAGTTCTTAGGCTCTTAATCTCGAAATTGAGGAACACCATGAAACACTAAAAGAGAGCTCGAAGACTAGGAAAGAAAACTAGAAGACTAAGCTTTGAAAGTCTTCTAAATCCAAGCATCTCGACATTGATCATCCTTGTGCAACATCATCCCTTCCTATTGCTTCACCAGAATCGGTGTCCCTTGTGGAGATCTCTGTCGTAGCGTCAAGGGGAGAATCCGAGAAGCAGAACTAGTCCGCGCTGCCTTCGCTACGCCATCTCCGCCATAGAGGATCTCATCCACGAAACATCCACCATCCAAACGGGAAACTGTTTTAAACACTCGGGTGGATGTTCACCCGTTTCTTGCATATCATCTAAATGGTTATGAAAAATTTTCAAAAAAAAAACATGATAGGTTAATATATAATATATCATCTCACAAATATGCAAGTTCAAATTCAACTTTTATAAGTTGTAAGTATAACAGGACGTTCATCTCACAAATATGCAAGTTTAAATTTAACTTTTACAAGTTGTAAGTGTAACAGTACGTCCATCGGATAGATTAATATCCATCTCCCCATCCAAACCCGTTGTTGCACCATCTGTCGAATCCGGCTGTGGACGCTCGGAGGCAAGAGCTAGCTCACCCGTCCCACACACACACCCAACGACGTCACAAGCGCCTCCGAACAACGCCAACTGATAACTTGGCAGCTCCTACGTGCCGACGTCGCGGTACTTGCCGGCGCTCCTAGCGCACGCACCGTCGAACCACACCGTCACCGACCAACTACCCACCGCCGCCGACTTCTGCCTCATCTGCCATCGTGGCCCTAGCCCAAGTTATCATCGTGGCAATTGCCGAGGCTCCTAAGTGTGCCACGGCCGAGGCAAAGTTCTAACTGAATCAGAGTATCAGACAGCCACCACCGACGCTACTTCTGCTTCATCTGCCATCGCCGTACTAGTTCAAGTTGTCGCTGTGGCAATCATGGGCCCTCCTAGCGTGCCACCCAACCGGACAGCCACGACATCCCCCATCACTGTTGTTATTGCCGCGCCCTGACCCCTATCGTCGTCGCTCTTAGCGCGTCGACGAGCCGACCAGCCACTGTCGTGCAGATGAAAAAAAAAACACATATTGGCCTGAGAGATCTGCTTAGTTCCAGTGCAGGTCCAACATGCTGTGAGATGCGGGCGTGCTAGTCAGTTTGATCTTGCAACTGACAAGATATATAAACAGCAGATAAAACAGCCGATCGACTAACAAGCCGATGGAGTAATTCCAGCCGATAGCCGATATTAGCCGATGCCGATTCTAGCCGATGTCGATAGGGTTTTGAACTATCGGCTATATGTCTAATGTAGGCAATGATATAAAGACAATTGGCTGATGATAATAAAATATAAAAATATAATCCAATAGAAACCAATCGGCTAATAATAAATATTGATCCGATGGTTAAAGCATACATCGGCTAAAAGTCCGATGTCATAAAATCCAATCGATTTAGATAAACAGTGAAACCTTTTTTGCAATCGGCTAAATCCAACTTGTATGTAATCTTCGTAAGCCGATGAACGTCCAGATAACTTATCGGCTAGCACCTCGATAAAACACTAGCATGAACCTATCGGCTTAACAAGATTTATATTATCAACAACAATCTAGTAGGTCGGACCTAACCGATGCAACACGGATTAGATATGATAATCTAATACTCGATGAGCCAATAGATCTGTCTAATGTGATGGATATAACAAATCTATTTATAACAGCATTGCGATTGTAGAGATATATCGGCTAAGACAGAATATCAGACCTAACTAAACCGATGCGTCTCTAAACACAATGCAATTAATTAGAGATATAATTGAGATATCAGCTAGGCAAATATATCAACCAAACTGGAGCGATCCAAGAGATCGGAGCAATGCAGCCTTGAACAACACCAATGTAGCCGATGGATTCACCAGGGCCGACGGAACGTAGGACTTACCCCTTCGCTGAAGATCGGGCTGAACCAATGCAGCCCCGCGTCAGGTGCCAAATTCCGCCGGTTGATAAGTAAAACCTCAGAAAAGAGGATGACGATGCGCCGAGAGTAGTATTGATCGAGAGATAAATTGCAATGACCCTGGATGTACATATTTGTACCCATGGGTAGATATTAGTTCTTGTAGGACAAGAAAGAAACTTTCCTAAAGATAAAATGAAAACATAAAGTCTTTATTGGATACTAAATACACTTTCCTAAAGATAAAAGGAAACTAAACCCTGCCTAATTAATAGATAAACTGCCATGTCGTATCCTCCTTGAACTCGGACTCTTTTAGATAAGCTACCTTTAACTAATCTTTACCCGAATCCATCAAGAATACAAATGTTGGCATTGATAGTTTCCATCGGTCAATTCTAGGACTTTGAAGCCGATACTGACTCTAAGCCGATGATTACTTTGTGCTTACCAAATTTTGTTGTTAACATGTCGCAACCACCATCACCGGCCAGCCACCCTGATCATTGTTGTTGACTCAGCATTCGCCAGGCTGAGCAGTCCACATACATGCCGCCATCTCCATGGCACTGTCGTTGCCGCCCCTTTCTCCTAGAGCCGCCGCAGCGCTCTTCGACACACCTACTGCATCGTCGAGCAGTCGTGCTACCACCTCCTCCATCGACCATAGCCGCCTCTTCTGCTGCACCGGATCCACCCACACCAACCACCAGATACAGTCAAGCCCTCATTCCCGGATCCCATATCCATCCATGCCACTACTGTGCTGCCCAGTCCAAGGAATGGAGCGAAGGAGGAAGCCCCGCCGCTGCCCTCCCGGCGGCCACATGCACTCCAGTGCCTTGCTCCGACGGCAGCGAGGTTGGAAAATGGGTGGCAGCGGCTAGGGTTTATCTGGGGAGAAGGAAAAGGAGAGGGAGGGGGGGGGGGTCCACTTCCAGCTTAATTAGCCTAGATCTTATTGACAAATCAGTTGCTGGGTGCACAGACATGTTATTCTTTTTGCATGACCAATCTTGAACACTTAGGTATGTTAGTTGAGTGGACACTGGTCTATCTGAAACATCTCTTCACATGGAGGCTGCGAATGAGTTTTCTTTTTGAGAGACCAAAGTTTCGTTGTATGTTAAGTGATAAAGCCTTGGTAAGAAATGCTACCACAAACGAACTAATAACTCCAAACGTAAAGTGGAGGAACCCGTATGGGTGACTCGAGTGGCGACAAACTCTAGCACCTCCACCTCCTTGGACGGGCTGCGGCGGTGCTTTCGGCATCCCAGTCTTCTTGGAGGCATCATCTAGAATTAAGGTCTTGTTATTGCTTAGCATGCCTTAGGGCACGTCCAGTGTTTAGTTCGACTAAAACTTCCATGAAAGCCAAACAAAAGTTCTGTTTGACCACCACAGTGTAAAAATCGATTGTGGGACCCATGCAAAAAAATCACAATCTCAGCTGCCTATGCTCTCCTCCTGGACCTGATAGCCGTGCACAACAAATATTTTTTTAAACTGGATGTGTTCGGCTTCTCTTTAAAGATCGTTTTTTCCTCTGACACTTACCAACCGGCTTTCACAGTGTGGTCAGTTCTTTTTTTTTTTACGCAAAGTTTGATTTTAGTCAGACACGGGAGGATCTGTTAAGCAGGCTTGGAAATTTCGGACCCCTCCAATACAATATTATTTTAGCCAAAATTTCTAATTTTTTAATTTTTCATGAATTTTGGTAATATTTGTTCTAATTTAACTAAATTTTGTTCAAAATTTCGGTCTATCAGTGACCTCCGATCAAATCAGTTAAACCGAGAAAATAAACCATGCTCTTAAGAGAGTTTGGTATGGTTCAATATCAAAACTTATAGTCTTGCAATTTTTTCTACCCTTTATCTTTTTCCCTGACTATTTAGTATGGATCGTTTAAAAAAAAGAAAGCCCATTGGTGACCAAGGGCTTGTTTGATTCAAGACCATCCCTAGCCTTACCAACCTTTTGGCAATGGCAAAAATTGGTTGTTGCCAAAAATATTGGCACAAATTGGCTAAGCCTATGATTGGTTTCTACCAAAGTTGAATTTTGGCATTCAATCAAGCCAAATAATTTGGCAATAACATTTTCTTATCTATGGATATAACATATGGCAAATATTTTGGCATTACCATTTTCTTTTTGCCAAACATGTTATTCCTTTTGAATGACCAATCTTGACACCTTATGTATGTTAGTAGTGGAATCGACACTATTCTATCTAAAACATCTCTTTACATAGAGGCCGCTAATAATTTTTCTTTGAGATAACCAAATTTTCCTTACAAGTTAAGCAACAAAGCCCATTGGTAAGATATGCTACGACAAATGAACTAATAACTCCAAACGTAAAGCGGAGGATCCCGCATTTCCCACGTGGGTGACTCGAGCGGTGACAAACCCTAGTACCTCCACCCCCTTGGGTGGGTTGTGGTGGCACTTTCGGCACCGTATTTTCCTTGGACGGATCATTTAGAAAGTCCTATTATTGCCTAGTATGCCTTGACAGTTTAGGCAACACTCTTGGATGGTGGTGTCCTTTGCCCTGGTGATCTAGTAGCCCATGGATGTTTAGTTATTTGGACATGGTGTTGGATGGTGCGCTCGTGGGCCTGTTGTAGGTCTGGTGCCAACCAGTCATGCTTAGAAATAGCCGGATAGGTGCACAGTGCTAGTTCTTTACTTGGTGGTTTGTGCAGCGCTATCGACATGTGGTGGTGTGCTTTTTCTTTGTCCGGATAATAATCTCATAGGGCTATACTCTTGTTATTTTGCTGCTATATTATTATGATAACTTGGTATGGTTCGTTTTTTCTTTTTTTGGAAAAACACCTAGTTGATCAAGGGCTTGTTTGGTTCAAGTGCATTCCTAATCTTACCTTTTCTTTTTTTTTTTAATGGCAAGAATTGTTCATTGCAAAAAAAAAAAGAGATAAAAATTGGCTAGGCTTACGTTTTGGTTCTTACCAAAGTTGTACTTTGAGACCAAATATATGGCAAAATTTTGGCATAACCTTTTTTTTTTTTGCTTGGTTGAGCTTGGTACAAACCAAACAGTCACAAAATAGACTGTCATGAATCACGCCTACTAAATTCCTTTGAACCGAACTAGAATATATTTGCTCTTAAAAGATTTCTTGATTTCAATTGGTACCATTTACTAGTAGAAACTTAAATTTAAATTTTAAAAACAAAATCATAATATTGTTGTTATGGAAATTTTAGTCATTTTAGTACTTTTGTAATATATGAGTTGGGTTATACTTGAGATATCCTAAATTGCTTTAAGATGAACAATTGCTAGGTATATCAAAGATGAGCTAAAAACAATGCAGGCATTCCTTAGAGCTGCTGAAGTTATGAAAAAGAAAGATGAACTATTAAAGGTTTGGGCAGAGCAAATACGTGACCTGTCGTATGACATTGAAGATTCCCTTGATGAATTTAAAGTCCATATTGAAAGCCAAACCCTATTTCGTCAGTTGGTGAAACTTAGAGAGCGCCACCGGATCGCTATCCGTATCCACAACCTCAAATCAAGAGTTGAAGAAGTGAGTAGCAGGAACACACGCTACAATTTAGTCGAGCCTATTTCCTCCGGCACAGAGGATGACATGGATTCCTATGCAGAAGACATTCGCAATCAATCAGCTCGAAATGTGGATGAAGCTGAGCTTGTTGGGTTTTCTGACTCCAAGAAAAGGCTGCTTGAAATGATCGATACCAATGCTAATGATGGTCCGGCCAAGGTAATCTGTGTTGTTGGGATGGGTGGTTTAGGCAAGACAGCTCTTTCGAGGAAGATCTTTGAAAGCGAAGAAGACATTAGGAAGAACTTCCCTTGCAATGCTTGGATTACAGTGTCACAATCATTTCACAGGATTGAGCTACTTAAAGATATGATACGCCAACTTCTTGGCCCCAGTTCTCTGGATCAACTCTTGCAAGAATTGCAAGGGAAGGTGGTGGTGCAAGTACATCATCTTTCTGAGTACCTGATAGAAGAGCTCAAGGAGAAGAGGTACTTTGTTGTTCTAGATGATCTATGGATTTTACATGATTGGAATTGGATAAATGAAATTGCATTTCCTAAGAACAATAAGAAGGGCAGTCGAATAGTAATAACCACTCGGAATGTTGATCTTGCGGAGAAGTGTGCCACAGCCTCACTGGTGTACCACCTTGATTTCTTGCAGATGAACGATGCCATAACATTGCTACTGAGAAAAACAAATAAAAATCATGAAGACATGGAATCAAATAAAAATATGCAAAAGATGGTTGAACGAATTGTAAATAAATGTGGTCGTCTACCATTAGCAATACTTACAATAGGAGCTGTGCTTGCAACTAAACAGGTGTCAGAATGGGAGAAATTCTATGAACACCTTCCTTCAGAACTAGAAATAAACCCAAGCCTGGAAGCTTTGAGGAGAATGGTGACCCTAGGTTACAACCACCTACCATCCCATCTGAAACCATGCTTTTTGTATCTAAGTATCTTTCCTGAGGATTTTGAAATCAAAAGGAATCGTCTAGTAGGTAGATGGATAGCAGAAGGGTTTGTTAGACCAAAGGTTGGGATGACGACTAAGGATGTCGGAGAAAGTTACTTTAATGAGCTAATCAACCGAAGTATGATTCAACGATCAAGAGTGGGCATAGCAGGAAAAATTAAGACTTGTCGAATTCATGATATCATCCGTGATATCACAGTTTCAATCTCGAGACAGGAAAATTTTGTATTGTTACCAATGGGAGATGGCTCTGATTTAGTTCAGGAAAACACTCGCCACATAGCATTCCATGGGAGTATGTCCTGCAAAACAGGATTGGATTGGAGCATTATTCGATCATTAGCTATTTTTGGTGACAGACCCAAGAGTCTAGCACATGCAGTTTGTCCAGATCAATTGAGGATGTTACGGGTCTTGGATCTTGAAGATGTGACATTCTTAATCACTCAAAAAGATTTCGACCATATTGCATTGTTGTGCCACTTGAAATACTTGAGTATTGGATATTCGTCATCCATATATTCACTTCCCAGATCCATTGGTAAACTACAGGGCCTACAAACTTTGAACATGCCGAGCACATACATTGCAGCACTACCAAGTGAGATCAGTAAACTCCAATGTCTGCATACTCTTCGTTGTAGTAGAAAGTTTGTTTATGACAACTTTAGTCTAAACCACCCAATGAAGTGCATAACTAACACAATATGCCTGCCTAAAGTATTCACACCTTTAGTTAGTCGCGATGATCGTGCAAAACAAATTGCTGAATTGCACATGGCCACCAAAAGTTGCTGGTCTGAATCATTCGGTGTGAAGGTACCCAAAGGAATAGGTAAGTTGCGAGACTTGCAGGTTCTAGAGTATGTAGATATCAGGCGGACCAGTAGTAGAGCAATCAAAGAGCTGGGGCACTTAAGCAAGTTGAGGAAATTAGGTGTGATAACAAAAGGCTCGACAAAGGAAAAATGTAAGATACTTTATGCAGCCATTGAGAAGCTCTCTTCCCTCCAATCTCTCTATGTGAATGCTGCGTTATTATCAGATATTGAAACACTTGAGTGCCTAGATTCTATTTCATCTCCTCCTCCCCTACTGAGGACACTCGGGTTGAATGGAAGTCTTGAAGAGATGCCTAACTGGATTGAGCAGCTCACTCACCTGAAGAAGATCTACTTATTGAGGAGCAAACTAAAGGAAGGTAAAACCATGCTGATACTTGGGGCATTGCCCAACCTCATGGTCCTTTATCTTTATCGGAATGCTTACCTTGGGGAGAAGCTAGTATTCAAAACGGGAGCATTCCCAAATCTTAGAACACTTTGTATTTACGAATTGGATCAGCTAAGAGAGATCAGATTTGAGGACGGCAGCTCACCCCTGTTGGAAAAGATAGAAATAGGCAAGTGCAGGTTGGAATCTGGGATTATTGGTATCATTCACCTTCCAAAGCTCAAGGAGATTCCAATTACATACGGAAGTAAAGTGGCTGGGCTTGGTCAGCTGGAGGGAGAAGTGAACACACACCCAAATCGCCCCGTGCTGCTAATGTACAGTGACCGAAGGTATCACGACCTGGGGGCTGAAGCCGAAGGATCTTCTATAGAAGTGCAAACAGCAGATCCTGTTCCTGATGCCGAAGGATCAGTCACTGTAGCAGTGGAAGCAACGGATCCCCTTCCCGAGCAGGAGGGAGAGAGCTCGCAGTCGCAGGTGATCACGTTGACGACGAATGATAGGTCAGTCACTCCCTACATGGCAGCTTAATTAACTTGTTTCTAATTCTCTTCTTGTTCAGTATTAGCCATCAGGTGAGGGCGATGATTTCAACTCACTTTTCATCTCTCTCGTTTTCTTAACCTGACA

>Pi9-Type8

GCTTGCATATGACGTTATGTAGATAGAGATGGCCAATATAATGCGCTGGAAAGTCCAAAGTGAGGATGCAAAACATCTTATAGTGGGTAGTGGAGCCATGCAAGGACCTGGTCTAAAGCGCACCTAAACCGTCATGTGGACTGCCATTATAGTTAAAGTTAGGGGGAATATGATTCTCTTCATGTGCACCTAAACTTTAATATGCAGTGAAACGAACGCTATGATATGATGATAAGCTTAATTCCTCTCTCTGCTCAGACTGTTCAGTGCAAAAGCTACCAACGAGCTTGTCTCCTTGTGCGGTCGTGAGCTTGCTTGTGCTAAGCTTGAAGGGAGAGTCGAACGAATCCATGGCGGAGACGGTGCTGAGCATGGCGAGGTCGCTGGTGGGCAGTGCCATCAGCAAGGCCGCCTCTGCCGCTGCCAATGAGACGAGCCTCCTGCTCGGCGTCGAGAAGGACATCTGGTACGTACTGCACTGCGCTCTCGTTTATCCTAGCTCGGTTGTATCGACTTCCAGCTTAATCTTTTTAATAATGAATAAAAACCCGGACTTGTTATCCATAAGGGCACCCACAATGGTTATCTATAGGCTCTCTATAAGAGATCTATGTCAGCATATTTTCCTACTTGGAAGGTATTAAATGAAGAGAGAGAGCAAAGCTATCTACTAACTTAGAGATAGTCTATAGAGAAAAACGAGGCAATGCATGAGAGAGTTATAGATACCAATGTAGACATACTATTAAGGTGGTTTACTATTAATCGAGTCTATTGCTGAGATGTACATGTTTTATAGATAGCACCTTACTTTACCATTGCGGGTGCTCTAAGTGGATATACACAGTCAAAACACGCGACAAGTTCTTAGGCTCTTAATTAATCTCGAAATTGAGGAACACCATGAAACACTAAAAGAGAGCTCGAAGACTAGGAAAGAAAACTAGAAGACTAAGCTTTGAAAGTCTTCTAAATCCAAGCATCTCGACATTGATCATCCTTGTGCAACATCAACCCTTCCTATTGCTTCACCAGAATCGGCGTCCCTTGTGGAGATCTCTGTTGTAACGTCAAGGGGAAAATCGGAGAAGCAGAACTAGTCCGCGCTGCCTTCGCTACGCCATCTCCGCCTTAGAGGATCTCATCCACGAAACATCCACCATCCAAACGGGAAACAGTTTTAAACACTCGTGGACGTTCACCCGTTCATCTAAATGGTTATGAAAAATTTTCAAAAAAAATAACATGATAGGTTAACATGTAATATATCATCTTATAAATATGCAAGTTCAAATTTGATTTCTACAAGTTGTAACAAAAATAACAAATTTTACTGTGAATATACGTAAACTAGTTAAAGTTTAATTTGTTATTTTTGTTACAACTTGTAGAAGTCGAATTTAAATCTGTATGTTTGTGAAATGAGATATTACATATTAACCTATCTTATAATTTTTTTTAGAAATTTTTTAGAATTATTTAGGTGGCATACAAGAAACGGATGGACATCCACAAAGAGATTAGTATCCATCTCCACATCCAAACCCGTTGTTGCACCATCTGTCGAATCTGTCGAATCCGGCTGTGGACGCTCGGAGGCAAGAGCTAGCTCACCCGTCCCACACACACACCCAACGACGTCACAAGCGCCTCCGAACAACGCCAACTGATAACTTGGCAGCTCCTACGTGCCGACGTCGCGGTACTTGCCGGCGCTCCTAGCGCATGCACCGTCGAACCACACCGTCACCGACCAGCTACCCACCGCCGCCGACTTCTGCCTCATCTGCCATCGTCGCCCTAGCCCAAGTTATCATCGTGGCAATTGCCGAGGCTCCTAAGTGTGCCACGGCCGAGGCAAAGTTCTAACTGAATCAGACAGCCACCACCGACACTTCTGCTTCATCTGCCATCGCCGTACTAGTTCAAGTTGTCGCTGTGGCAATCACTGTTGTTATTGCCGCGCCCTGACCCCTATCGTCGTCGCTCTTAGCGCGTCGTCGAGCCGACCAGCCACTGTCGTGCAGATGAAAAAAAAAAACACATTTTGGCCTGAGAGATCTGCTTAGTTCCATTGCAGGTCCAACATGCTGTGAGATGCGGGCGTGCCAGTCAGTTTGATCTTGCAACTGACAAGATATATAAACAGCAGATAAAACAGCCTATCGACTAACAAGCCGATGGAGTAATTCCAGCCGATAGCCGATATTAGCCGATGCCGATTCTAGCCGATGTCGATAGGGTTTTGAACTATCGGCTATATGTCCAATGTAGGCAATGATATAAAGACAATTGGCTGATGATAATAAAATATAAAAATATAATCCAATAGAAACCAATCGGCTAATAATAAGTATTGATCCGATAGTTAAAGCATACATCGGCTAAAAGTCCGATGTCATAAAATCCAATCGATTTAGATAAACAGTGAAACCTTTGTTGCAATCGGCTAAATCCAACTTGTATGTAATCTTCGTAAGCCGATGAACGTCCAGATAACTTATCGGCTAGCACCTCGATAAAACACTAGCATGAACCTATCGGCTTAACAAGATTTATATTATCAACAACAATCTAGTAGGTCGGACCTAACCGATGCAACACGTATTAGATATGATAATCTAATACTCGATGAGCCAATAGATCTGTCTAATGTGATGGATATAACAAATCTATTTATAAAAGCATTGCGATTGTAGAGATATATCGGCTAAGACAGAATATCAGACCTAACTAAACCGATGCGTCTCTAAACACAATGCAATTAATTAGAGATATAATTGAGATATCAGCTAGGCAAATATATCAACCAAACTAGAGCGATCCAAGAGATCGGAGCAATGCAGCCTTGAACAACACCAATGTAGCCGATGGATTCACCAGGGTCGACGGAATGTAGGACTTACCCCTTCCCTGAAGATCGGGCTGAACCAATGCAGTCCCATGTCAGGTGCCAAATTCCGCCGGTTGATAAGTAAAACCTCAGAAAAGAGGATGACGATGCGCCGAGAGTAGTATTGATCGAGAGATAAATTGCAATGACCCTGGATGTACATATTTGTACCCATGGGTAGATATTAGTTCTTGTAGGACAAGAAAGAAACTTTCCTAAAGATAAAATGAAAACATAAAGTTTTTATTGGATACTAAACACACTTTCCTAAAGATAAAAGGAAACTAAACCCTGCCTAATTAATAGATAAACTGCCATGTCGTATCCTCCTTGAACTCGAACTCTTTTAGATAAGCTTCCTTTAACTAATCTTTACCCGAATCCATCAAGAATACAAATGTTGGCATTGATAGTTTTCATCGGTCAATTCTAGGACTTTGAAGCCGATACTGACTCTAAGCCGATGACTACTTTGGGCTTACCAAATTTTGTTGTTAACATGTCGCGACCACCATCACCGGCCAGCCACCCTGATCATTGTTGTTGACTCAGCATTCGCCAGGCTGAGCAGTCCACATACATGCCGCCATCTCCATGGCACTGTCGTTGCCGCCCCTTTCTCCTAGAGCCGCCGCAGCGCTCTTCGACACACCTACTGCATCGTCGAGCAGTCGTGCTACCACCTCCTCCATCGACCATAGCCGCCTCTTCTGCTGCACCGGATCCACCCACACCAACCACCAGATACAGTCAAGCCCTCATTCCCGGATCCCATATCCATCCATGCCACTACTGTGCTGCCCAGTCCAAGGAATGGAGCGAAGGAGGAAGCCCCGCCGCTGCCCTCCCGGCGGCCACATGCACTCCAGTGCCTTGCTCCGACGGCAGCGAGGTTGGAAAATGGGTGGCAGCGGCTAGGGTTTATCTGGGGAGAAGGAAAAGGAGAGGGAGGGGGGGGGGGGTCCACTTCCAGCTTAATTAGCCTAGATCTTATTGACAAATCAGTTGCTGGGTGCACAAACATGTTATTTTTTTTGCATGACCAATCTTGAACACTTAGGTATGTTAGTTGAGTGGACACTGGTCTATCTGAAACATCTCTTCACATGGAGGCTGCGAATGAGTTTTCTTTTTGAGAGACCAAAGGTTCGTTGTATGTTAAGTGATAAAGCCTTGGTAAGAAATGCTACCACAAACGAACTAATAACTCCAAACGTAAAGTGGAGGAACCCGTATGGGTGACTCGAGTGGCGACAAACTCTAGCACCTCCACCTCCTTGGACGGGCTGCGGCGGTGCTTTCGGCATCCCAGTCTTCTTGGAGGCATCATCTAGAATTAAGGTCTTGTTATTGCTTAGCATGCCTTAGGGCACGTCCAGTGTTTAGTTCGACTAAAACTTCCATGAAAGCCAAACAAAAGTTCTGTTTGACCACCACAGTGTAAAAATCGATTGTGGGACCCATGCAAAAAAATCACAATCTCAGCTGCCTATGCTCTCCTCCTGGACCTGATAGCCGTGCACAACAAATATTTTTTTAAACTGGATGTGTTCGGCTTCTCTTTAAAGATCGTTTTTTCCTCTGACACTTACCAACCGGCTTTCACAGTGTGGTCAGTTCTTTTTTTTTTTACGCAAAGTTTGATTTTAGTCAGACACGGGAGGATCTGTTAAGCAGGCTTGGAAATTTCGGACCCCTCCAATACAATATTATTTTAGCCAAAATTTCTAATTTTTTAATTTTTCATGAATTTTGGTAATATTTGTTCTAATTTAACTAAATTTTGTTCAAAATTTCGGTCTATCAGTGACCTCCGATCAAATCAGTTAAACCGAGAAAATAAACCATGCTCTTAAGAGAGTTTGGTATGGTTCAATATCAAAACTTATAGTCTTGCAATTTTTTCTACCCTTTATCTTTTTCCCTGACTATTTAGTATGGATCGTTTAAAAAAAAGAAAGCCCATTGGTGACCAAGGGCTTGTTTGATTCAAGACCATCCCTAGCCTTACCAACCTTTTGGCAATGGCAAAAATTGGTTGTTGCCAAAAATATTGGCACAAATTGGCTAAGCCTATGATTGGTTTCTACCAAAGTTGAATTTTGGCATTCAATCAAGCCAAATAATTTGGCAATAACATTTTCTTATCTATGGATATAACATATGGCAAATATTTTGGCATTACCATTTTCTTTTTGCCAAACATGTTATTCCTTTTGAATGACCAATCTTGACACCTTATGTATGTTAGTAGTGGAATCGACACTATTCTATCTAAAACATCTCTTTACATAGAGGCCGCTAATAATTTTTCTTTGAGATAACCAAATTTTCCTTACAAGTTAAGCAACAAAGCCCATTGGTAAGATATGCTACGACAAATGAACTAATAACTCCAAACATAAAGCGGAGGATCCCGCATTTCCCACGTGGGTGACTCGAGCGGTGACAAACCCTAGTACCTCCACCCCCTTGGGTGGGTTGTGGTGGCACTTTCGGCACCGTATTTTCCTTGGACGGATCATTTAGAAAGTCCTATTATTGCCTAGTATGCCTTGACAGTTTAGGCAACACTCTTGGATGGTGGTGTCCTTTGCCCTGGTGATCTAGTAGCCCATGGATGTTTAGTTATTTGGACATGGTGTTGGATGGTGCGCTCGTGGGCCTGTTGTAGGTCTGGTGCCAACCAGTCATGCTTAGAAATAGCCGGATAGGTGCACAGTGCTAGTTCTTTACTTGGTGGTTTGTGCAGCGCTATCGACATGTGGTGGTGTGCTTTTTCTTTGTCCGGATAATAATCTCATAGGGCTATACTCTTGTTATTTTGCTGCTATATTATTATGATAACTTGGTATGGTTCGTTTTTTCTTTTTTTGGAAAAACACCTAGTTGATCAAGGGCTTGTTTGGTTCAAGTGCATTCCTAATCTTACCTTTTCTTTTTTTTTTCAATGGCAAGAATTGTTCATTGCAAAAAAAAAAAGAGATAAAAATTGGCTAGGCTTACGTTTTGGTTCTTACCAAAGTTGTACTTTGAGACCAAATATATGGCAAAATTTTGGCATAACCTTTTTTTTTTTTGCTTGGTTGAGCTTGGTACAAACCAATCAGTCACAAAATAGACTGTCATGAATCACGCCTACTAAATTCCTTTGAACCGAACTAGAATATATTTGCTCTTAAAAGATTTCTTGATTTCAATTGGTACCATTTACTAGTAGAAACTTAAATTTAAATTTTAAAAACAAAATCATAATATTGTTGTTATGGAAATTTTAGTCATTTTAGTAATTTTGTAATATATGAGTTGGGTTATACTTGAGATATCCTAAATTGCTTTAAGATGAACAATTGCTAGGTATATCAAAGATGAGCTAAAAACAATGCAGGCATTCCTTAGAGCTGCTGAAGTTATGAAAAAGAAAGATGAACTATTAAAGGTTTGGGCAGAGCAAATACGTGACCTGTCGTATGACATTGAAGATTCCCTTGATGAATTTAAAGTCCATATTGAAAGCCAAACCCTATTTCGTCAGTTGGTGAAACTTAGAGAGCGCCACCGGATCGCTATCCGTATCCACAACCTCAAATCAAGAGTTGAAGAAGTGAGTAGCAGGAACACACGCTACAATTTAGTCGAGCCTATTTCCTCCGGCACAGAGGATGACATGGATTCCTATGCAGAAGACATTCGCAATCAATCAGCTCGAAATGTGGATGAAGCTGAGCTTGTTGGGTTTTCTGACTCCAAGAAAAGGCTGCTTGAAATGATCGATACCAATGCTAATGATGGTCCGGCCAAGGTAATCTGTGTTGTTGGGATGGGTGGTTTAGGCAAGACAGCTCTTTCGAGGAAGATCTTTGAAAGCGAAGAAGACATTAGGAAGAACTTCCCTTGCAATGCTTGGATTACAGTGTCACAATCATTTCACAGGATTGAGCTACTTAAAGATATGATACGCCAACTTCTTGGTCCCAGTTCTCTGGATCAACTCTTGCATGAATTGCAAGGGAAGGTGGTGGTGCAAGTACATCATCTTTCTGAGTACCTGATAGAAGAGCTCAAGGAGAAGAGGTACTTTGTTGTTCTAGATGATCTATGGATTTTACATGATTGGAATTGGATAAATGAAATTGCATTTCCTAAGAACAATAAGAAGGGCAGTCGAATAGTAATAACCACTCGGAATGTTGATCTAGCGGAGAAGTGTGCCACAGCCTCACTGGTGTACCACCTTGATTTCTTGCAGATGAACGATGCCATTTCATTGCTACTGAGAAAAACAAATAAAAATCATGAAGACATGGAATCAAATAAAAATATGCAAAAGATGGTTGAACGAATTGTAAATAAATGTGGTCGTCTACCATTAGCAATACTTACAATAGGAGCTGTGCTTGCAACTAAACAGGTGTCAGAATGGGAGAAATTCTATGAACAACTTCCTTCAGAACTAGAAATAAACCCAAGCCTGGAAGCTTTGAGGAGAATGGTGACCCTAGGTTACAACCACCTACCATCCCATCTGAAACCATGCTTTTTGTATCTAAGTATCTTTCCTGAGGATTTTGAAATACAAAGGAATCGTCTAGTAGGTAGATGGATAGCAGAAGGGTTTGTTAGACCAAAGGTTGGGATGACGACTAAGGATGTCGGAGAAAGTTACTTTAATGAGCTAATCAACCGAAGTATGATTCAACGATCAAGAGTGGGCACAGCAGGAAAAATTAAGACTTGTCGAATCCATGATATCATCCGTGATATCACAGTTTCAATCTCGAGACAGGAAAATTTTGTATTATTACCAATGGGAGATGGCTCTGATTTAGTTCAGGAAAACACTCGCCACATAGCATTCCATGGGAGTATGTCCTGCAAAACAGGATTGGATTGGAGCATTATTCGATCATTAGCTATTTTTGGTGACAGACCCAAGAGTCTAGCACATGCAGTTTGTCCAGATCAATTGAGGATGTTACGGGTCTTGGATCTTGAAGATGTGACATTCTTAATCACTCAAAAAGATTTCGACCGTATTGCATTGTTGTGCCACTTGAAATACTTGAGTATTGGATATTCGTCATCCATATATTCACTTCCCAGATCCATTGGTAAACTACAGGGCCTACAGACTTTGAACATGTCAAGCACATACATTGCAGCACTACCAAGTGAGATCAGTAAACTCCAATGTCTGCATACTCTTCGTTGTATAAGAGAGCTTGAATTTGACAACTTTAGTCTAAATCACCCAATGAAGTGCATAACTAACACAATATGCCTGCCTAAAGTATTCACACCTTTAGTTAGTCGCGATAATCGTGCAAAACAAATTGCTGAATTTCACATGGCCACCAAAAGTTTCTGGTCTGAATCATTCGGTGTGAAGGTACCCAAAGGAATAGGTAAGTTGCGAGACTTACAGGTTCTAGAGTATGTAGATATCAGGCGGACCAGTAGTAGAGCAATCAAAGAGCTGGGGCAGTTAAGCAAGTTGAGGAAATTAGCTGTGATAACAAAAGGCTCGACAAAGGAAAAATGTAAGATACTTTATGCAGCCATTGAGAAGCTCTCTTCCCTCCAATCTCTCTATATGAATGCTGCGTTATTATCAGATATTGAAACACTTGAGTGCCTAGATTCTATTTCATCTCCTCCTCCCCTACTGAGGACACTCGGGTTGAATGGAAGTCTTGAAGAGATGCCTAACTGGATTGAGCAGCTCACTCACCTGAAGAAGTTCAACTTATGGAGTAGTAAACTAAAGGAAGGTAAAAACATGCTGATACTTGGGGCATTGCCCAACCTCATGTTCCTTTCTCTTTATCATAATTCTTATCTTGGGGAGAAGCTAGTATTCAAAACGGGAGCATTCCCAAATCTTAGAACACTTGTGATTTTCAATTTGGATCAGCTAAGAGAGATCAGATTTGAGGACGGCAGCTCACCCCAGTTGGAAAAGATAGAAATCTCTTGCTGCAGGTTGGAATCAGGGATTATTGGTATCATTCACCTTCCAAGGCTCAAGGAGATTTCACTTGAATACAAAAGTAAAGTGGCTAGGCTTGGTCAGCTGAAGGGAGAAGTGAACACACACCCAAATCGCCCCGTGCTGCGAATGGACAGTGACCGAAGGGATCACGACCTGGGGGCTGAAGCCGAAGGATCTTCTATAGAAGTGCAAACAGCAGATCCTGTTCCTGATGCCCAAGGATCAGTCACTGTAGCAGTGGAAGCAACGGATCCCCTTCCCGAGCAGGAGGGAGAGAGCTCGCAGTCGCAGGTGATCACGTTGACGACGAATGATAGGTCAGTCACTCCCTACATGGCAGCTTAATTAACTTGTTTCTAATTCTCTTCTTGTTCAGTATTAGCCATCAGGTGAGGGCGATGATTTCAACTCACTTTTCATCTCTCTCGTTTTCTTAACCTGACA

>Pi9-Type9

GCTTGCATATGACGTTATGTAGATAGAGATGGCCAATATAATGCGCTGGAAAGTCCAAAGTGAGGATGCAAAACATCTTATAGTGGGTAGTGGAGCCATGCAAGGACCTGGTCTAAAGCGCACCTAAACCGTCATGTGGACTGCCATTATAGTTAAAGTTAGGGGGAATATGATTCTCTTCATGTGCACCTAAACTTTAATATGCAGTGAAACGAACGCTATGATATGATGATAAGCTTAATTCCTCTCTCTGCTCAGACTGTTCAGTGCAAAAGCTACCAACGAGCTTGTCTCCTTGTGCGGTCGTGAGCTTGCTTGTGCTAAGCTTGAAGGGAGAGTCGAACGAATCCATGGCGGAGACGGTGCTGAGCATGGCGAGGTCGCTGGTGGGCAGTGCCATCAGCAAGGCCGCCTCTGCCGCTGCCAATGAGACGAGCCTCCTGCTCGGCGTCGAGAAGGACATCTGGTACGTACTGCACTGCGCTCTCGTTTATCCTAGCTCGGTTGTATCGACTTCCAGCTTAATCTTTTTAATAATGAATAAAAACCCGGACTTGTTATCCATAAGTGGATATACACAGTCAAAACACGCGACAAGTTCTTAGGCTCTTAATTAATCTCGAAATTGAGGAACACCATGAAACACTAAAAGAGAGCTCGAAGACTAGGAAAGAAAACTAGAAGACTAAGCTTTGAAAGTCTTCTAAATCCAAGCATCTCGACGTTGATCATCCTTGTGCAACATCAACCCTTCCTATTGCTTCACCAGAATCGGCGTCCCTTGTGGAGATCTCTGTTGTAACGTCAAGGGGAAAATCGGAGAAGCAGAACTAGTCCGCGCTGCCTTCGCTACGCCATCTCCGCCTTAGAGGATCTCATCCACGAAACATCCACCATCCAAACGGGAAACAGTTTTAAACACTCGTGGACGTTCACCCGTTCATCTAAATGGTTATGAAAAATTTTCAAAAAAAATAACATGATAGGTTAACATGTAATATATCATCTTATAATTATGCAAGTTCAAATTTGATTTCTACAAGTTGTAACAAAAATAACAAATTTTACTATGAATATACGTAAACTAGTTAAAGTTTAATTTGTTATTTTTGTTACAACTTGTAGAAGTCGAATTTAAATCTGCATGTTTGTGAAATGATATATTACATATTAACCTATCTTATAATTTTTTTAGAAAATTTTTAAAATTATTTATGTGGCATACAAGAAACGGGTGGATATCCACAAAGAGATTAGAATCCATCTCCCCATCCAAACCCGTTGTTGCACCATCTGTCGAATCTGTCGAATCCGGCTGTGGATGCTCGGAGGCAAGAGCCAGCTCACCCGTCCCCCCACACACACCCAACGACGTCACAAGCGCCTCCGAACAACGCCAACTGATAAGTTGGCAGCTCCTACGTGCCGACGTCGCCGTACTTGCCGGCGCTCCTAGCGCACGCATCGTCGAACCACACCGTCACCGACCAACTACCCACCGCCGCCGACTTCTGCCTCATCTGCCATCGTCGCCCTAGCCCAAGTTATCATCGTGGCAATTGCCGAGGCTCCTAAGTGTGCCACGGCCGAGGCAAAGTTCTAACTGAATCAGACAGCCACCACCGACACTTCTGCTTCATCTGCCATCGCCGTACTAGTTCAAGTTGTCGCTGTGGCAATCATTGGCCCTCCTAGCGTGCCACCCAACCGGACAACCACGACATCCCCCATCACTGTTGTTATTGCCGCCCTGACACCTATCGTCGTCGCTCTTAGGGCGTCGTCGAGCCGACCAGCCACTGTCGTGCAGATGAACGTCCGAGAGATCTGCTTAGCTCCAGTGCAGGTCCAAGATGCTGATGAGATCGGACGTACCAGTTAGTTTGATCTTGCAATTGACAAGATATATAAACAGCAGATAAAACAGCTGATCGACTAACAAGCCGATGGAGTAATTCCAGCCGATAGCCGATATTAGCCGATGCCGATTCCAGCCGATGTCGATAGGGTTTTGAACTATAGGCTATATGTTCAATGTAGGCAATGATATAAAGACAATTGGTTGATGATAATAAAATATAAAAATATAATCCAATAGAAACCAATCGGCTAATAATAAGTATTGATCCAACGGTTAAAGCATACATCGGCTAAAAGTCCGATGTCATAAAATCCAATCGATTTAGATAAACAGTGAAACCTTTGTTGCAATCGGCTAAATCCAACTTGTATGTAATCTTCGTAAGCCGATGAACGTCCAGATAACTTATCGGCTAGCACCTCGATAAAACACTAGCATGAACCTATCGGCTTAACAAGATTTATATTATCAACAACAATCTAGTAGGTCGGACCTAACCGATGCAGCACGAGATTAGATATGATAATCTAATACTCGATGAGCCGATAGATCTGTCTAATGTGATGGATATAACAAATCTATTTATAACAACATTGCGATTGTAGAGATATGTCGGCTAAGACAGAATATCAGACCTAACTAAACCGATGTGTCTCTAAACACAATGCAATTAATTAGAGATAGAATTAAGATATCAGGTAGGCAAATATATCAACCAAACTAGAGCGATTCAAGCGATTAGAGCAATGCAGCCTTGAACAACACCAATATAGCCGATGGATTCACCAGGGCCAATGGAACATAGGACTTACCCCTTCGCTGAAGATCGGGCTGAATCAATGTAGCCCCGCATCAGGTGCCAAATTCCGCCAGTTGATAAGTAAAACCTTAGAAAAGAGGATGACGATGCGCCGAGAGTAGTATTGATCGAGAGATAAATTGCAATGACCCGATGCGCCGAGAGTAGTATTGATCGAGAGATAAATTGCAATGACCTCGGATGTATATATTTGTACCCATGGGTATATATTAGTTCTTGTAGGACAAGAAAGAAACTTTCCTAAAGATAAAAGGAAAACATAAAGTCCTTATCGGATACTAAACACACTTTCCTAAAGATAAAAGGAAACTAAACCCTGCCTAATTAATAGATAAACTGCTATGTCGTATCCTCCTTGAACTCGGACTCTTTTAGATAAGCTTCCTTTAACTAATCTTTACCCGAATCCATCAAGAATACAAATGTTGGCATTGATAGTTTTCATCGGTCAATTATAGGACTTTGAAGCCGATACTGACTCTAAGCCGATGACTACTTTGGGCTTACCAAATTTTGTTGTTAATATGTCGCGACCACCATCACCGGCCAGCCACCCTGATCATTGTTGTTGACTCAGCATTCGCCAGGCTGAGCAGTCCACATACATGCCGCCATCTCCATGGCAGTGTCGTTGCCGCCCCTTTCTCCTAGAGCCGCCGCAGCGCTCTTCGACACACCTACTGCATCGTCGAGCAGTCGTGCTACCACCTCCTCCATCGACCATAGCCGCCTCTTCTGCTGCACCGGATCCACCCACACCAACCACCAGATACAGTCAAGCCCTCATTCCTGGATCCCATATCCATCCATGCCACTACTGTGCTGCCCAGTCCAAGGAATGGAGCGAAGGAGGAAGCCCCGCCGCTGCCCTCCCGGCGGCCACATGCACTCCAGTGCCTTGCTCCGACGGCAGCGAGGTTGGAAAATGGGTGGCAGCGGCTAGGGTTTATCTGGGGAGAAGGAAAAGGAGAGGGAGGGGGGGGGAGGGTCCACTTCCAGCTTAATTAGCCTAGATCTTATTGACAAATCAGTTGCTGGGTGCACAAACATGTTATTTTTTTTGCATGACCAATCTTGAACACTTAGGTATGTTAGTTGAGTGGACACTGGTCTATCTGAAACATCTCTTCACATGGAGGCTGCGAATGAGTTTTCTTTTTGAGAGACCAAAGTTTCGTTGTATGTTAAGTGATAAAGCCTTGGTAAGAAATGCTACCACAAACGAACTAATAACTCCAAACGTAAAGTGGAGGAACCCGTATGGGTGACTCGAGTGGCGACAAACTCTAGCACCTCCACCTCCTTGGACGGGCTGCGGCGGTGCTTTCGGCATCCCAGTCTTCTTGGAGGCATCATCTAGAATTAAGGTCTTGTTATTGCTTAGCATGCCTTAGGGCACGTCCAGTGTTTAGTTCGACTAAAACTTCCATGAAAGCCAAACAAAAGTTCTGTTTGACCACCACAGTGTAAAAATCGATTGTGGGACCCATGCAAAAAAATCACAATCTCAGCTGCCTATGCTCTCCTCCTGGACCTGATAGCCGTGCACAACAAATATTTTTTTAAACTGGATGTGTTCGGCTTCTCTTTAAAGATCGTTTTTTCCTCTGACACTTACCAACCGGCTTTCACAGTGTGGTCAGTTCTTTTTTTTTTTACGCAAAGTTTGATTTTAGTCAGACACGGGAGGATCTGTTAAGCAGGCTTGGAAATTTCGGACCCCTCCAATACAATATTATTTTAGCCAAAATTTCTAATTTTTTAATTTTTCATGAATTTTGGTAATATTTGTTCTAATTTAACTAAATTTTGTTCAAAATTTCGGTCTATCAGTGACCTCCGATCAAATCAGTTAAACCGAGAAAATAAACCATGCTCTTAAGAGAGTTTGGTATGGTTCAATATCAAAACTTATAGTCTTGCAATTTTTTCTACCCTTTATCTTTTTCCCTGACTATTTAGTATGGATCGTTTAAAAAAAAGAAAGCCCATTGGTGACCAAGGGCTTGTTTGATTCAAGACCATCCCTAGCCTTACCAACCTTTTGGCAATGGCAAAAATTGGTTGTTGCCAAAAATATTGGCACAAATTGGCTAAGCCTATGATTGGTTTCTACCAAAGTTGAATTTTGGCATTCAATCAAGCCAAATAATTTGGCAATAACATTTTCTTATCTATGGATATAACATATGGCAAATATTTTGGCATTACCATTTTCTTTTTGCCAAACATGTTATTCCTTTTGAATGACCAATCTTGACACCTTATGTATGTTAGTAGTGGAATCGACACTATTCTATCTAAAACATCTCTTTACATAGAGGCCGCTAATAATTTTTCTTTGAGATAACCAAATTTTCCTTACAAGTTAAGCAACAAAGCCCATTGGTAAGATATGCTACGACAAATGAACTAATAACTCCAAACGTAAAGCGGAGGATCCCGCGTTTCCCACGTGGGGAACTCGAGCGGTGACAAACCCTAGCACCTCCACCCCCTTGGGTGGGTTGTGGTGGCACTTTCGGCACCGCATTTTCCTTGGATGGATCATTTAGAAGGTCCTATTATTGCCTAGTCTGCCTTGACAGTTTAGGCAACACTCTTGGATGGTAGTGTCCTTCGCCCTGGTGATCTAGTAGCCCATGGATGTTTAGTTATTTGGACATGGTGTTGGATGGTGCGCTCGTGGGCCTGTTGTAGGTCTGGTGCCAACCAGTCATGCTTAGAAATAGCCGGATAGGTGCATGGTGCTAGTTCTTTACTTGGTGGTTTGTGCAGCGCTACCGACATGTGGTGGTGTGCTTTTTCTTTGTCCGGATAATAATCTCATAGGGCTATACTCTTGTTATTTTGCTGCTATATTATTATGATAACTTGGTATGGTTCGTTTTTTTTTTCGGAAAAACACCTAGTTGATCAAGGGCTTGTTTGGTTCAAGTGCATTCTAAAAAACTAGAATATTAGGGAAAGACCTATGGCAAGAATTGTTCATTGCAAAAAAAAAAAGAGATAAAAATTGGCTAGGCTTACGTTTTGGTTCTTACCAAAGTTGTACTTTGAGACCAAATATATGGCAAAATTTTGGCATAACCTTTTTTTTTTTTGCTTGGTTGAGCTTGGTACAAACCAAACAGACCCAAAATAGACTGTCATGAATCACGCCTACTAAATTCCTTTGAACCAAACTAGAATATACTTGCTCTTAAAAGATTTCTTGATTTCACTCGGTACCATTTACTAGTACAAACTTAAATTTAAATTTTAAAAACAAAATCATAATATTGTTATTATGGAAATTTTAGTCATTTTAGTACTTTTGTAATATATGAGTTGGGTTATACTTGAGATATCCTAAATTGCTTTAAGATGAACAATTGCTAGGTATATCAAAGATGAGCTAAAAGCGATGCAGGCATTCCTTAGAGCTGCTGAAGTTATGAAAAAGAAAGATGAACTATTAAAGGTTTGGGCAGAGCAAATATGTGACCTGTCGTATGACATTGAGGATTCCCTTGATGAATTTAAAGTCCATATTGAAAGCCAAAATCTGTTTCGTCAGATGGTGAAGCTCAGAGAACGCCACCGGATTGCTATCCGGATCCACAACCTTAAATCAAGAGTTGAAGAAGTGAGTAGTAGGAACACACGCTACAGTTTAGTCAAGCCTATTTCCTCTAGCACAGAGGATGACATAGATTCCTATGCAGAAGACATTCGTAATCTGTCAGCTCGCAATGTGGATGAAGCTGAGCTTGTTGGGTTTTCTGACTCCAAGAAAAGGTTGCTTGAAATGATCGATACCAATGCTAATGATGGTCCGGCCAAAGTAATCTGTGTTGTTGGGATGGGTGGTTTAGGCAAGACAGCTCTTTCAAGGAAGATCTTTGAAAGCGAAGAAGACATTAGGAAGAACTTCCCTTGCAATGCTTGGATTACTGTGTCACAATCATTTCACAGGATTGAGCTACTCAAAGATATGATACGCCAACTTCTAGGCCCCATTTCTCTGAATCTACTCTTGAAAGAATTGCAAGGGAAGGTGGTGGTGCAAGTACATCATCTTTCTGAGTACCTGCTAGAAGAGCTCAAGGAGAAGAGGTACTTTGTTGTTCTAGATGATCTATGGTTTTTACATGATTGGAATTGGATAAATGATATTGCATTTCCTAAGAACAATAAGATGGGCAGTCGAATAGTAATAACCACTCGGAGTGTTGATCTAGCAGAGAAGTGTGCCACAGCCTCACTGGTCTACCACCTTGATTTCTTGCAGATGAACGATGCCATAACATTGCTACTAAGAAAAACAAATAAAAAACATGAAGACATGGAATCAAATAAAAATATGCAAAATATGGTTGAACGAATTGTAAATAAGTGTGGTCGTCTACCATTAGCAATACTTACAATAGGAGCTGTGCTTGCAACTAAACATGTGTCAGAATGGGAGAAATTCTATGAAAAGCTTCCTTCAGAACTAGAAATAAACCCAAGCCTGGAAGCTTTGAGGAGAATGGTGATCCTAGGTTACAACCACCTACCATCCCATCTGAAACCATGCTTTTTGTATCTAAGTATCTTTCCTGAGGATTTTGAAATCAAAAGGAATCGTCTGGTAGGTAGATGGATAGCAGAAGGGTTTGTTAGACCGCAGGTTGGGATGATGACTAAGGATGTCGGAGAAAGTTACTTTAATGAGCTAATCAGCCGAAGTATGATTCAACGATCAAGAGTGGGCATAGCAGGAAAAATTCAGAGTTGTCGAGTCCATGATATCATCCGTGATATCACAGTTTCAATCTCGAGACAGGAAAATTTTGTATTGTTACCAATGGGAGATGGCTCTGATTTAGTTCAGGAAAACACTCGCCACATAGCATTCCATGGGAGTATGTCCTGCAAAACAGGATTGGATTGGAGCATTATTCGATCATTAGCTATTTTTGGTGGACCCAAGAGTCTAGCACATGCAGTTTGTCCAGATCAATTGAGGATGTTACGGGTCTTGGATCTTGAAGATGTGACATTCTTAATCACTCAAAAAGATTTCGACCGTATTGCATTGTTGTGCCACTTGAAATACTTGAGTATTGGATATTCGTCATGCATATATTCACTTCCCAGATCCATTGGTAAACTACAGGGCCTACAAACTTTGAACATGCCGAGCACATACATTGCAGCACTACCAAGTGAGATCAGTAAACTCCAATGTCTGCATACTCTTCGTTGTATAAGAGAGTTTCATTATGACAACTTTAGTCTAAACCACCCAATGAAGTGCATAACTAACACAATATGCCTGCCTAAAGTATTCACACCTTTAGTTAGTCGCGATGATCGTGCAAAACAAATTGCTGAATTTCACATTGCCACCAAAAGATTCTGGTCTGAATCATTCGGTGTGAAGGTACCCAAAGGAATAGGTAAGTTGCGAGACTTACAGGTTCTAGAGTATGTAGATATCAGGCGGACCAGTAGTAGAGCAATCAAAGAGCTGGGGCAGTTAAGCAAGTTGAGGAAATTAGGTGTGATAACAAAAGGCTCGACAAAGGAAAAATGTAAGATACTTTATGCAGCCATTGAGAAGCTCTCTTCCCTCCAATCTCTCTATGTGAATGCTGCGTTATTATCAGATATTGAAACATTTGAGTGCCTAGATTCTATTTCATCTCCTCCTCCCCTACTGAGGACACTCAGGTTGAATGGAAGTCTTGAAGAGATGCCTAACTGGATTGAGCAGCTCACTCACCTGAAGAAGATCTACTTATTGAAGAGCAAACTAAAGGAAGGTAAAACCATGCTGATACTTGGGGCATTGCCCAACCTCATGGTCCTTCATCTTTATCGGAATGCTTACCTTGGGGAGAAGCTAGTATTCAAAACAGGAGCATTCCCAAATCTTAGAACACTTCGTATTTACGAATTGGATCAGTTAAGAGAGATGAGATTTGAGGACGGCAGCTCACCCCTGTTGGAAAAGATAGAAATAGGCAACTGCAGGTTGGAATCAGGGATTATTGGTATCATCCACCATCCAAAGCTCAAGGAGATTTCAATTAGATACGGAAGTAAAGTGGCTGGGCTTGGTCAGCTGGAGGGAGAAGTGAACACACACCCAAATCGCCCCGTGCTGCGAATGGACAGTGACCGAAGGGATCATGACCTGGGGGCTGAAGCCGAAGGATCTTCTATAGAAGTGCAAACAGCAGATCCTGTTCCTGATGCCGAATGATCAGTCACTGTAGCAGTGGAAGCAACGGATCCCCTTCCCGAGCAGGAGGGAGAGAGCTCGCAGTCGCAGGTGATCACGTTGACGACGAATGATAGTCAGTCGCTCCCTACATGGCAGCTTAATTAACTTGTTTCTAATTCTCTTCTTGTTCAGTATTAGCCATCAGGTGAGGGCGATGATTTCAACTCACTTTTCATCTCTCTCGTTTTCTTAACCTGACA

>Pi9-Type10

GCTTGCATTTGACGTTATGTAGATAGAGGTGGAGAATATAATGCGCTGGAAAGTCCAAAGTGAGGATGCAAAACATCTTAGAGCAGAGCAGGTATAATAGCAGGCTACAAACCAGCTGCAAACATATTTTAAGGAGATAAATGAGGAGAGAGAAGGACAGCGGGCTGCAGATTTGTAGCCAGCTGTAGCACGGACTCTAAGACACAGTGTGTGTATGACAGGCGGGATCATATATTAATAGTGTAGTATGTAATTATTGTATGAATGAGCTATTAGATTGGCTATAGATGAATTGGAGCTAGTAGTTGGCTATACTATTGAACTTGCTATTATAGTGGGTAGTGGAGCCATGCAAGGACCTGGTATCTAAAGCGCACCTAAACCGTAATGTGGACAGCCATTATAGTTAAAGTTAGGGGGAATATGATTCTCTTCATGTGCACCTAAACCGTAATGTGCAGTGAAACGAACGCTATGATATGATGATAAGCTTAATTCCTCTCTCTGCTCAGACTGTTCAGGGCAAAAACTACCAACGAGAGCTTGTCTCCTTGTGCGGTCGTGAGCTTGCTTGTGCTAAGCTTGAAGGGAGAGTCGAACGAATCCATGGCGGAGACGGTGCTGAGCATGGCGAGGTCGCTGGTGGGCAGTGCCATCAGCAAGGCCGCCTCTGCCGCTGCCAATGAGACGAGCCTCCTGCTCGGCGTCGAGAAGGACATCTGGTACGTACTGCACTGCTCTCGTTTATCCTAGCAAGTTCTTAGGCTCTTAATCTCGAAATTGAGGAACACCATGAAACACTAAAAGAGAGCTCGAAGACTAGGAAAGAAAACTAGAAGACTAAGCTTTGAAAGTCTTCTAAATCCAAGCATCTCGACATTGATCATCCTTGTGCAACATCATCCCTTCCTATTGCTTCACCAGAATCGGTGTCCCTTGTGGAGATCTCTGTCGTAGCGTCAAGGGGAGAATCCGAGAAGCAGAACTAGTCCGCGCTGCCTTCGCTACGCCATCTCCGCCATAGAGGATCTCATCCACGAAACATCCACCATCCAAACGGGAAACTGTTTTAAACACTCGGTTGGATGTTCACCCGTTTCTTGCCTGTCATCTAAATGGTTATGAAAAATTTTCAAAAAAAAACATGATAGGTTAATATATAATATATCATCTCACAAATATGCAAGTTCAAATTCAACTTTTATAAGTTGTAAGTATAACAGGACGTTCATCTCACAAATATGCAAGTTTAAATTTAACTTTTACAAGTTGTAAGTGTAACAGGACGTCCATCGGATAGATTAATATCCATCTCCCCATCCAAACCCGTTGTTGCACCATCTGTCGAATCCGGCTGTGGACGCTCGGAGGCAAGAGCTAGCTCACCCGTCCCACACACACACCCAACGACGTCACAAGCGCCTCCGAACAACGCCAACTGATAACTTGGCAGCTCCTACGTGCCGACGTCGCGGTACTTGCCGGCGCTCCTAGCGCACGCACCGTCGAACCACACCGTCACCGACCAACTACCCACCGCCGCCGACTTCTGCCTCATCTGCCATCGTCGCCCTAGCCCAAGTTATCATCGTGGCAATTGCCGAGGCTCCTAAGTGTGCCACGGCCGAGGCAAAGTTCTAATTAACTGAATCAGAGTATCAGACAGCCACCACCGACGCTACTTCTGCTTCATCTGCCATCGCCGTACTAGTTCAAGTTGTCGCTGTGGCAATCATGGGCCCTCCTAGCGTGCCACCCAACCGGACAGCCACGACATCCCCCATCACTGTTGTTATTGCCGCGCCCTGACCCCTATCGTCGTCGCTCTTAGCGCGTCGTCGAGCCGACCAGCCACTGTCGTGCAGATGAAAAAAAAAAACACATATGGGCCTGAGAGATCTGCTTAGTTCCAGTGCAGGTCCAACATGCTGTGAGATGCGGGCGTGCCAGTCAGTTTGATCTTGCAACTGACAAGATATATAAATAGCAGATAAAACAGCCTATCGACTAACAAGCCGATGGAGTAATTCCAGCCGATAGCCGATATTAGCCGATGCCGATTCTAGCCGATGTCGATAGGGTTTTGAACTATCGGCTATATGTCCAATGTAGGCAATGATATAAAGACAATTGGCTGATGATAATAAAATATAAAAATATAATCCAATAGAAACCAATCGGCTAATAATAAGTATTGATCCGATAGTTAAAGCATACATCGGCTAAAAGTCCGATGTCATAAAATCCAATCGATTTAGATAAACAGTGAAACCTTTGTTGCAATCGGCTAAATCCAACTTGTATGTAATCTTCGTAAGCCGATGAACGTCCAGATAACTTATCGGCTAGCACCTCGATAAAACACTAGCATGAACCTATCGGCTTAACAAGATTTATATTATCAACAACAATCTAGTAGGTCGGACCTAACCGATGCAACACGTATTAGATATGATAATCTAATACTCGATGAGCCAATAGATCTGTCTAATGTGATGGATATAACAAATCTATTTATAAAAGCATTGCGATTGTAGAGATATATCGGCTAAGACAGAATATCAGACCTAACTAAACCGATGCGTCTCTAAACACAATGCAATTAATTAGAGATATAATTGAGATATCAGCTAGGCAAATATATCAACCAAACTGGAGCGATCCAAGAGATCGGAGCAATGCAGCCTTGAACAACACCAATGTAGCCGATGGATTCACCAGGGCCGACGGAACGTAGGACTTACCCCTTCCCTGAAGATCGGGCTGAACCAATGCAGTCCCACGTCAGGTGCCAAATTCCGCCGGTTGACAAGTAAAACCTCAGAAAAGAGGATGACGATGCGCCGAGAGTAGTATTGATCGAGAGATAAATTGCAATGACCCTGGATGTACATATTTGTACCCATGGGTAGATATTAGTTCTTGTAGGACAAGAAAGAAACTTTCCTAAAGATAAAATGAAAACATAAAGTCTTTATTGGATACTAAACACACTTTCCTAAAGATAAAAGGAAACTAAACCCTGCCTAATTAATAGATAAACTGCCATGTCGTATCCTCCTTGAACTCGGACTCTTTTAGATAAGCTTCCTTTAACTAATCTTTACCCGAATCCATCAAGAATACAAATGTTGGCATTGATAGTTTTCATCGGTCAATTCTAGGACTTTGAAGCCGATACTGACTCTAAGCCGATGACTACTTTGGGCTTACCAAATTTTGTTGTTAACATGTCGCGACCACCATCACCGGCCAGCCACCCTGATCATTGTTGTTGACTCAGCATTCGCCAGGCTGAGCAGTCCACATACATGCCGCCATCTCCATGGCAGTGTCGTTGCCGCCCCTTTCTCCTAGAGCCGCCGCAGCGCTCTTCGACACACCTACTGCATCGTCGAGCAGTCGTGCTACCACCTCCTCCATCGACCATAGCCGCCTCTTCTGCTGCACCGGATCCACCCACACCAACCACCAGATACAGTCAAGCCCTCATTCCCGGATCCCATATCCATCCATGCCACTACTGTGCTGCCCAGTCCAAGGAATGGAGCGAAGGAGGAAGCCCCGCCGCTGCCCTCCCGGCGGCCACATGCACTCCAGTGCCTTGCTCCGACGGCAGCGAGGTTGGAAAATGGGTGGCAGCGGCTAGGGTTTATCTGGGGAGAAGGAAAAGGAGAGGGAGGGGGGGGGGGGAGGGTCCACTTCCAGCTTAATTAGCCTAGATCTTATTGACAAATCAGTTGCTGGGTGCACAAACATGTTATTTTTTTTGCATGACCAATCTTGAACACTTAGGTATGTTAGTTGAGTGGACACTGGTCTATCTGAAACATCTCTTCACATGGAGGCTGCGAATGAGTTTTCTTTTTGAGAGACCAAAGTTTCGTTGTATGTTAAGTGATAAAGCCTTGGTAAGAAATGCTACCACAAACGAACTAATAACTCCAAACGTAAAGTGGAGGAACCCGTATGGGTGACTCGAGTGGCGACAAACTCTAGCACCTCCACCTCCTTGGACGGGCTGCGGCGGTGCTTTCGGCATCCCAGTCTTCTTGGAGGCATCATCTAGAATTAAGGTCTTGTTATTGCTTAGCATGCCTTAGGGCACGTCCAGTGTTTAGTTCGACTAAAACTTCCATGAAAGCCAAACAAAAGTTCTGTTTGACCACCACAGTGTAAAAATCGATTGTGGGACCCATGCAAAAAAATCACAATCTCAGCTGCCTATGCTCTCCTCCTGGACCTGATAGCCGTGCACAACAAATATTTTTTTAAACTGGATGTGTTCGGCTTCTCTTTAAAGATCGTTTTTTCCTCTGACACTTACCAACCGGCTTTCACAGTGTGGTCAGTTCTTTTTTTTTTTACGCAAAGTTTGATTTTAGTCAGACACGGGAGGATCTGTTAAGCAGGCTTGGAAATTTCGGACCCCTCCAATACAATATTATTTTAGCCAAAATTTCTAATTTTTTAATTTTTCATGAATTTTGGTAATATTTGTTCTAATTTAACTAAATTTTGTTCAAAATTTCGGTCTATCAGTGACCTCCGATCAAATCAGTTAAACCGAGAAAATAAACCATGCTCTTAAGAGAGTTTGGTATGGTTCAATATCAAAACTTATAGTCTTGCAATTTTTTCTACCCTTTATCTTTTTCCCTGACTATTTAGTATGGATCGTTTAAAAAAAAGAAAGCCCATTGGTGACCAAGGGCTTGTTTGATTCAAGACCATCCCTAGCCTTACCAACCTTTTGGCAATGGCAAAAATTGGTTGTTGCCAAAAATATTGGCACAAATTGGCTAAGCCTATGATTGGTTTCTACCAAAGTTGAATTTTGGCATTCAATCAAGCCAAATAATTTGGCAATAACATTTTCTTATCTATGGATATAACATATGGCAAATATTTTGGCATTACCATTTTCTTTTTGCCAAACATGTTATTCCTTTTGAATGACCAATCTTGACACCTTATGTATGTTAGTAGTGGAATCGACACTATTCTATCTAAAACATCTCTTTACATAGAGGCCGCTAATAATTTTTCTTTGAGATAACCAAATTTTCCTTACAAGTTAAGCAACAAAGCCCATTGGTAAGATATGCTACGACAAATGAACTAATAACTCCAAACGTAAAGCGGAGGATCCCGCATTTCCCACGTGGGTGACTCGAGCGGTGACAAACCCTAGTACCTCCACCCCCTTGGGTGGGTTGTGGTGGCACTTTCGGCACCGTATTTTCCTTGGACGGATCATTTAGAAAGTCCTATTATTGCCTAGTATGCCTTGACAGTTTAGGCAACACTCTTGGATGGTGGTGTCCTTTGCCCTGGTGATTTAGTAGCCCATGGATGTTTAGTTATTTGGACATGGTGTTGGATGGTGCGCTCGTGGGCCTGTTGTAGGTCTGGTGCCAACCAGTCATGCTTAGAAATAGCCGGATAGGTGCACAGTGCTAGTTCTTTACTTGGTGGTTTGTGCAGCGCTATCGACATGTGGTGGTGTGCTTTTTCTTTGTCCGGATAATAATCTCATAGGGCTATACTCTTGTTATTTTGCTGCTATATTATTATGATAACTTGGTATGGTTCGTTTTTTCTTTTTTTGGAAAAACACCTAGTTGATCAAGGGCTTGTTTGGTTCAAGTGCATTCCTAATCTTACCTTTTCTTTTTTTTTTCAATGGCAAGAATTGTTCATTGCAAAAAAAAAAAGAGATAAAAATTGGCTAGGCTTACGTTTTGGTTCTTACCAAAGTTGTACTTTGAGACCAAATATATGGCAAAATTTTGGCATAACCTTTTTTTTTTTTGCTTGGTTGAGCTTGGTACAAACCAATCAGTCACAAAATAGACTGTCATGAATCACGCCTACTAAATTCCTTTGAACCGAACTAGAATATATTTGCTCTTAAAAGATTTCTTGATTTCAATTGGTACCATTTACTAGTAGAAACTTAAATTTAAATTTTAAAAACAAAATCATAATATTGTTGTTATGGAAATTTTAGTCATTTTAGTACTTTTGTAATATATGAGTTGGGTTATACTTGAGATATCCTAAATTGCTTTAAGATGAACAATTGCTAGGTATATCAAAGATGAGCTAAAAACAATGCAGGCATTCCTTAGAGCTGCTGAAGTTATGAAAAAGAAAGATGAACTATTAAAGGTTTGGGCAGAGCAAATACGTGACCTGTCGTATGACATTGAAGATTCCCTTGATGAATTTAAAGTCCATATTGAAAGCCAAACCCTATTTCGTCAGTTGGTGAAACTTAGAGAGCGCCACCGGATCGCTATCCGTATCCACAACCTCAAATCAAGAGTTGAAGAAGTGAGTAGCAGGAACACACGCTACAATTTAGTCGAGCCTATTTCCTCCGGCACAGAGGATGACATGGATTCCTATGCAGAAGACATTCGCAATCACTCAGCTCGAAATGTGGATGAAGCTGAGCTTGTTGGGTTTTCTGACTCCAAGAAAAGGCTGCTTGAAATGATCGATACCAATGCTAATGATGGTCCGGCCAAGGTAATCTGTGTTGTTGGGATGGGTGGTTTAGGCAAGACAGCTCTTTCGAGGAAGATCTTTGAAAGCGAAGAAGACATTAGGAAGAACTTCCCTTGCATTGCTTGGATTACAGTGTCACAATCATTTCACAGGATTGAGCTACTTAAAGATATGATACGCCAACTTCTTGGCCCCAGTTCTCTGGATCAACTCTTGCAAGAATTGCAAGGGAAGGTGGTGGTGCAAGTACATCATCTTTCTGAGTACCTGATAGAAGAGCTCAAGGAGAAGAGGTACTTTGTTATTCTAGATGATCTATGGATTTTACATGATTGGAATTGGATAAATGAAATTGCATTTCCTAAGAACAATAAGAAGGGCAGTCGAATAGTAATAACCACTCGGAATGTTGATCTAGCGGAGAAGTGTGCCACAGCCTCACTGGTGTACCACCTTGATTTCTTGCAGATGAACGATGCCATAACATTGCTACTGAGAAAAACAAATAAAAATCATGAAGACATGGAATCAAATAAAAATATGCAAAAGATGGTTGAACGAATTGTAAATAAATGTGGTCGTCTACCATTAGCAATACTTACAATAGGAGCTGTGCTTGCAACTAAACATGTGTCAGAATGGGAGAAATTCTATGAACAACTTCCTTCAGAACTAGAAATAAACCCAAGCCTGGAAGCTTTGAGGAGAATGGTGACCCTAGGTTACAACCACCTACCATCCCATCTGAAACCATGCTTTTTGTATCTAAGTATCTTTCCTGAGGATTTTGAAATCAAAAGGAATCGTCTAGTAGGTAGATGGATAGCAGAAGGGTTTGTTAGACCAAAGGTTGGGATGACGACTAAGGATGTCGGAGAAAGTTACTTTAATGAGCTAATCAACCGAAGTATGATTCAACGATCAAGAGTGGGCATAGCAGGAAAAATTAAGACTTGTCGAATCCATGATATCATCCGTGATATCACAGTTTCAATCTCGAGACAGGAAAATTTTGTATTATTACCAATGGGAGATGGCTCTGATTTAGTTCAGGAAAACACTCGCCACATAGCATTCCATGGGAGTATGTCCTGCAAAACAGGATTGGATTGGAGCATTATTCGATCATTAGCTATTTTTGGTGACAGACCCAAGAGTCTAGCACATGCAGTTTGTCCAGATCAATTGAGGATGTTACGGGTCTTGGATCTTGAAGATGTGACATTCTTAATCACTCAAAAAGATTTCGACCGTATTGCATTGTTGTGCCACTTGAAATACTTGAGTATTGGATATTCGTCATCCATATATTCACTTCCCAGATCCATTGGTAAACTACAGGGCCTACAAACTTTGAACATGCTGAGAACATACATTGCAGCACTACCAAGTGAGATCAGTAAACTCCAATGTCTGCATACTCTTCGTTGTATAGGACAGTTTCCTTATGACAACTTTAGTCTAAACCACCCAATGAAGTGCATAACTAACACAATATGCCTGCCTAAAGTATTCACACCTTTAGTTAGTCGCGATGATCGTGCAAAACAAATTGCTGAATTGCACATGGCCACCAAAAGTTGCTGGTCTGAATCATTCGGTGTGAAAGTACCCAAAGGAATAGGTAAGTTGCGAGACTTGCAGGTTCTAGAGTATGTAGATATCAGGCGGACCAGTAGTAGAGCAATCAAAGAGCTGGGGCAGTTAAGCAAGTTGAGGAAATTAGGTGTGATAACAAAAGGCTCGACAAAGGAAAAATGTAAGATACTTTATGCAGCCATTGAGAAGCTCTCTTCCCTCCAATCTCTCTATGTGAATGCTGCGTTATTATCAGATATTGAAACACTTGAGTGCCTAGATTCTATTTCATCTCTCCCTCCTCCCCTACTGAGGACACTCGGGTTGAATGGAAGTCTTGAAGAGATGCCTAACTGGATTGAGCAGCTCACTCACCTGAAGAAGTTCTACTTATTAGGCAGCAAACTAAAGGAAGGTAAAACCATGCTGATACTTGGGGCATTGCCCAACCTCATGGTCCTTTATCTTTATGGGAATGCTTACCTTGGGGAGAAGCTAGTATTCAAAACGGGAGCATTCCCAAATCTTAGAACACTTCGTATTTACGAATTGGCTCAGCTAAGAGAGATGAGATTTGAGGATGGCAGCTCACCCCTGTTGGAAAAGATAGAAATCTCTTGCTGCAGGTTGGAATCAGGGATTATTGGTATCATTCACCTTCCAAGGCTCAAGGAGATTTCACTTGAATACAAAAGTAAAGTGGCTAGGCTTGGTCAGCTGGAGGGAGAAGTGAACACACACCCAAATCGCCCCGTGCTGCGAATGGACAGTGACCGAAGGGATCACGACCTGGGGGCTGAAGCCGAAGGATCTTCTATAGAAGTGCAAACAGCAGATCCTGTTCCTGATGCCGAAGGATCAGTCACTGTAGCAGTGGAAGCAACGGATCCCCTTCCCGAGCAGGAGGGAGAGAGCTCGCAGTCGCAGGTGATCACGTTGACGACGAACGATAGGTCAGTCACTCCCTACATGGCAGCTTAATTAACTTGTTTCTAATTCTCTTCTTGTTCAGTATTAGCCATCAGGTGAGGGCGATGATTTCAACTCACTTTTCATCTCTCTCGTTTTCTTAACCTGACA

>Pi9-Type11

GCTTGCATGACGTTATGTAGATAGAGATGGCGAATATAATGCGCTGGAAAGTCCCAAGTGAGGATGCAAAACATCTTATAGTGGGTAGTGGAGCCCTGCAAGGACCCTAATATCTAAAGCGCACCTAAACTGATATGGACATCCATTATAGTTAAAGTTAGGGGGAATATGATTCTCTTCCTGTGCACCTAAACCGTAATATGCAGTGAAACGAACGCTATGATACGATGATAAGCTTAATTCCTCTCTCTGCTCAGACTGTTCAGTGCAAAAGCTACCAACGAGAGCTTGGGCCTGTTTGGGGGAGCTTTAGATTCTGAGAAGCAGCTGTTTGGTAGCCAGCTTCTGAGAATCTGGAAAAGCTCTGAAACCCAGCTTCTCCAGCTTCTGACTTCTTAGTTCATTTTTCAGAATCTGTAACTACAGATTCTCAAAAGCTGTGGACTGTTTGGAGCAGCTTCTAGCAGAAGCAGCTTTTGGGAAAAGCTGCAGCTGGGATAAGCTCCCCCAAACAGGGCCCTTGTCTCCTTGTGCGGTCGTGAGCTTGCTTGTGCTAAGCTTGAAGGGAGAGTCGAACGAATCCATGGCGGAGACGGTGCTGAGCATGGCGAGGTCGCTGGTGGGCAGTGCCATCAGCAAGGCCGCCTCTGCCGCTGCCAATGAGACGAGCCTCCTGCTCGGCGTCGAGAAGGACATCTGGTACGTACTGCACTGCGCTCTCGTTTATCCTAGCTCGGTTGTATCGACTTCCAGCTTAATCTTTTTAATAATGAATAAAAACCCGGACTTTTTATCCATACGTGGATATACACAGTCAAAACACGCACAAGTTCTTAGGCTCTTAATCTCGAAATTCAGGAACACCATGAAACACTAAAAGAGAGCTCGAAGACTAGGAAAGAAAACTAGAAGACTAAGCTTTGAAAGTCTTCTAAATCCAAGCATCTCGACATTGATCATCCTTGTGCAACATCAACCCCTTCCTATTGCTTCACCAGAATCGGCGTCCCTTGTGGAGATCTCTCTGTCGTAGCGTCAAGGGGAGAATCCGAGAAGCAGAACTAGTCCGCGCTGCCTTCGCTACGCCATCTCCGCCATAGAGGATCTCATCCACGAAACATCCACCATCTAAACGGGAAACTGTTTTAAACACTCGGGTGGACGTTCACCCGTTTCTTGCATGTCATCTAAATGGTTATGAAAAATTTTAAAAAAAAAAAACATGATAGGTTAATATATAATATATCATTTCACAAATATGCAAGTTCAAATTCAACTTTTATAAGTTGTAAGTATAACAGGACGTTCATTTCACAAATATGCAAGTTTAAATTTAACTTTTACAAATTGTAAGTGTAACAGGACGTCCATCGGATAGATTAATATCCATCTCCCCATCCAAACCCGTTGTTGCACCATCTGTCGAATCCGGCTGTGGACGCTCGGAGGCAAGAGCTAGCTCACCCGTCCCACACACACACCCAACGACGTCACAAGCGCCTCCGAACAACGCCAACTGATAACTTGGCAGCTCCTACGTGCCGACGTCGCGGTACTTGCCGGCGCTCCTAGCGCACGCACCGTCGAACCACACCGTCACCGACCAACTACCCACCGCCGCCGACTTCTGCCTCATCTGCCATCGTCGCCCTAGCCCAAGTTATCATCGTGGCAATTGCCGAGGCTCCTAAGTGTGCCACGGCCGAGGCAAAGTTCTAACTGAATCAGAGTATCAGACAGCCACCACCGACGCTACTTCTGCTTCATCTGCCATCGCCGTACTAGTTCAAGTTGTCGCTGTGGCAATCATGGGCCCTCCTAGCGTGCCACACAACCGGACAGCCACGACATCCCCCATCACTGTTGTTATTGCCGCGCCCTGACCCCTATCGTCGTCGCTCTTAGCGCGTCGTCGAGCCGACCAGCCACTGTCGTGCAGATGAAAAAAAAACACATATTGGCATGAGAGATCTGCTTAGTTCCAGTGCAGGTCCAACATGCTGTGAGATGCGGGCGTGCCAGTCAGTTTGATCTTACAACTGACAAGATATATAAATAGCAGATAAAACAGCCTATCGACTAACAAGCCGATGGAGTAATTCCAGCCGATAGCCGATATTAGCCGATGCCGATTCTAGCCGATGTCGATAGGGTTTTGAACTATCGGCTATATGTCCAATGTAGGCAATGATATAAAGACAATTGGCTGATGATAATAAAATATAAAAATATAATCCAATAGAAACCAATCGGCTAATAATAAGTATTGATCCGATAGTTAAAGCATACATCGGCTAAAAGTCCGATGTCATAAAATCCAATCGATTTAGATAAACAGTGAAACCTTTGTTGCAATCGGCTAAATCCAACTTGTATGTAATCTTCGTAAGCCGATGAACGTCCAGATAACTTATCGGCTAGCACCTCGATAAAACACTAGCATGAACCTATCGGCTTAACAAGATTTATATTATCAACAACAATCTAGTAGGTCGGACCTAACCGATGCAACACGTATTAGATATGATAATCTAATACTCGATGAGCCAATAGATCTGTCTAATGTGATGGATATAACAAATCTATTTATAAAAGCATTGCGATTGTAGAGATATATCGGCTAAGACAGAATATCAGACCTAACTAAACCGATGCGTCTCTAAACACAATGCAATTAATTAGAGATATAATTGAGATATCAGCTAGGCAAATATATCAACCAAACTGGAGCGATCCAAGAGATCGGAGCAATGCAGCCTTGAACAACACCAATGTAGCCGATGGATTCACCAGGGCCGATGGAACGTAGGACTTACCCCTTCCCTGAAGATCGGGCTGAACCAATGCAGTCCCACGTCAGGTGCCAAATTCCGCCGGTTGACAAGTAAAACCTCAGAAAAGAGGATGACGATGCGCCGAGAGTAGTATTGATCGAGAGATAAATTGCAATGACCCTGGATGTACATATTTGTACCCATGGGTAGATATTAGTTCTTGTAGGACAAGAAAGAAACTTTCCTAAAGATAAAATGAAAACATAAAGTCTTTATTGGATACTAAACACACTTTCCTAAAGATAAAAGGAAACTAAACCCTGCCTAATTAATAGATAAACTGCCATGTCGTATCCTCCTTGAACTCGGACTCTTTTAGATAAGCTTCCTTTAACTAATCTTTACCCGAATCCATCAAGAATACAAATGTTGGCATTGATAGTTTTCATCGGTCAATTCTAGGACTTTGAAGCCGATACTGACTCTAAGCCGATGACTACTTTGGGCTTACCAAATTTTGTTGTTAACATGTCGCGACCACCATCACCGGCCAGCCACCCTGATCATTGTTGTTGACTCAGCATTCGCCAGGCTGAGCAGTCCACATACATGCCGCCATCTCCATGGCACTGTCGTTGCCGCCCCTTTCTCCTAGAGCCGCCGCAGCGCTCTTCGACACACCTACTGCATCGTCGAGCAGTCGTGCTACCACCTCCTCCATCGACCATAGCCGCCTCTTCTGCTGCACCGGATCCACCCACACCAACCACCAGATACAGTCAAGCCCTCATTCCCGGATCCCATATCCATCCATGCCACTACTGTGCTGCCCAGTCCAAGGAATGGAGCGAAGGAGGAAGCCCCGCCGCTGCCCTCCCGGCGGCCACATGCACTCCAGTGCCTTGCTCCGACGGCAGCGAGGTTGGAAAATGGGTGGCAGCGGCTAGGGTTTATCTGGGGAGAAGGAAAAGGAGAGGGAGGGGGGGGGGGGGGAAGGGTCCACTTCCAGCTTAATTAGCCTAGATCTTATTGACAAATCAGTTGCTGGGTGCACAAACATGTTATTTTTTTTGCATGACCAATCTTGAACACTTAGGTATGTTAGTTGAGTGGACACTGGTCTATCTGAAACATCTCTTCACATGGAGGCTGCGAATGAGTTTTCTTTTTGAGAGACCAAAGTTTCGTTGTATGTTAAGTGATAAAGCCTTGGTAAGAAATGCTACCACAAACGAACTAATAACTCCAAACGTAAAGTGGAGGAACCCGTATGGGTGACTCGAGTGGCGACAAACTCTAGCACCTCCACCTCCTTGGACGGGCTGCGGCGGTGCTTTCGGCATCCCAGTCTTCTTGGAGGCATCATCTAGAATTAAGGTCTTGTTATTGCTTAGCATGCCTTAGGGCACGTCCAGTGTTTAGTTCGACTAAAACTTCCATGAAAGCCAAACAAAAGTTCTGTTTGACCACCACAGTGTAAAAATCGATTGTGGGACCCATGCAAAAAAATCACAATCTCAGCTGCCTATGCTCTCCTCCTGGACCTGATAGCCGTGCACAACAAATATTTTTTTAAACTGGATGTGTTCGGCTTCTCTTTAAAGATCGTTTTTTCCTCTGACACTTACCAACCGGCTTTCACAGTGTGGTCAGTTCTTTTTTTTTTTACGCAAAGTTTGATTTTAGTCAGACACGGGAGGATCTGTTAAGCAGGCTTGGAAATTTCGGACCCCTCCAATACAATATTATTTTAGCCAAAATTTCTAATTTTTTAATTTTTCATGAATTTTGGTAATATTTGTTCTAATTTAACTAAATTTTGTTCAAAATTTCGGTCTATCAGTGACCTCCGATCAAATCAGTTAAACCGAGAAAATAAACCATGCTCTTAAGAGAGTTTGGTATGGTTCAATATCAAAACTTATAGTCTTGCAATTTTTTCTACCCTTTATCTTTTTCCCTGACTATTTAGTATGGATCGTTTAAAAAAAAGAAAGCCCATTGGTGACCAAGGGCTTGTTTGATTCAAGACCATCCCTAGCCTTACCAACCTTTTGGCAATGGCAAAAATTGGTTGTTGCCAAAAATATTGGCACAAATTGGCTAAGCCTATGATTGGTTTCTACCAAAGTTGAATTTTGGCATTCAATCAAGCCAAATAATTTGGCAATAACATTTTCTTATCTATGGATATAACATATGGCAAATATTTTGGCATTACCATTTTCTTTTTGCCAAACATGTTATTCCTTTTGAATGACCAATCTTGACACCTTATGTATGTTAGTAGTGGAATCGACACTATTCTATCTAAAACATCTCTTTACATAGAGGCCGCTAATAATTTTTCTTTGAGATAACCAAATTTTCCTTACAAGTTAAGCAACAAAGCCCATTGGTAAGATATGCTACGACAAATGAACTAATAACTCCAAACGTAAAGCGGAGGATCCCGCATTTCCCACGTGGGTGACTCGAGCGGTGACAAACCCTAGTACCTCCACCCCCTTGGGTGGGTTGTGGTGGCACTTTCGGCACCGTATTTTCCTTGGACGGATCATTTAGAAAGTCCTATTATTGCCTAGTATGCCTTGACAGTTTAGGCAACACTCTTGGATGGTGGTGTCCTTTGCCCTGGTGATCTAGTAGCCCATGGATGTTTAGTTATTTGGACATGGTGTTGGATGGTGCGCTCGTGGGCCTGTTGTAGGTCTGGTGCCAACCAGTCATGCTTAGAAAAAGCCGGATAGGTGCACAGTGCTAGTTCTTTACTTGGTGGTTTGTGCAGCGCTATCGACATGTGGTGGTGTGCTTTTTCTTTGTCCGGATAATAATCTCATAGGGCTATACTCTTGTTATTTTGCTGCTATATTATTATGATAACTTGGTATGGTTCGTTTTTTCTTTTTTTTGGAAAAACACCTAGTTGATCAAGGGCTTGTTTGGTTCAAGTGCATTCCTAATCTTACCTTTTCTTTTTTTTTTCAATGGCAAGAATTGTTCATTGCAAAAAAAAAAAGAGATAAAAATTGGCTAGGCTTACGTTTTGGTTCTTACCAAAGTTGTACTTTGAGACCAAATATATGGCAAAATTTTGGCATAACCTTTTTTTTTTTTGCTTGGTTGAGCTTGGTACAAACCAATCAGTCACAAAATAGACTGTCATGAATCACGCCTACTAAATTCCTTTGAACCGAACTAGAATATATTTGCTCTTAAAAGATTTCTTGATTTCAATTGGTACCATTTACTAGTAGAAACTTAAATTTAAATTTTAAAAACAAAATCATAATATTGTTGTTATGGAAATTTTAGTCATTTTAGTACTTTTGTAATATATGAGTTGGGTTATACTTGAGATATCCTAAATTGCTTTAAGATGAACAATTGCTAGGTATATCAAAGATGAGCTAAAAACAATGCAGGCATTCCTTAGAGCTGCTGAAGTTATGAAAAAGAAAGATGAACTATTAAAGGTTTGGGCAGAGCAAATACGTGACCTGTCGTATGACATTGAAGATTCCCTTGATGAATTTAAAGTCCATATTGAAAGCCAAACCCTATTTCGTCAGTTGGTGAAACTTAGAGAGCGCCACCGGATCGCTATCCGTATCCACAACCTCAAATCAAGAGTTGAAGAAGTGAGTAGCAGGAACACACGCTACAATTTAGTCGAGCCTATTTCCTCCGGCACAGAGGATGACATGGATTCCTATGCAGAAGACATTCGCAATCAATCAGCTCGAAATGTGGATGAAGCTGAGCTTGTTGGGTTTTCTGACTCCAAGAAAAGGCTGCTTGAAATGATCGATACCAATGCTAATGATGGTCCGGCCAAGGTAATCTGTGTTGTTGGGATGGGTGGTTTAGGCAAGACAGCTCTTTCGAGGAAGATCTTTGAAAGCGAAGAAGACATTAGGAAGAGCTTCCCTTGCATTGCTTGGATTACAGTGTCACAATCATTTCACAGGATTGAGCTACTTAAAGATATGATACGCCAACTTCTTGGCCCCAGTTCTCTGGATCAACTCTTGCAAGAATTGCAAGGGAAGGTGGTGGTGCAAGTACATCATCTTTCTGAGTACCTGATAGAAGAGCTCAAGGAGAAGAGGTACTTTGTTATTCTAGATGATCTATGGATTTTACATGATTGGAATTGGATAAATGAAATTGCATTTCCTAAGAACAATAAGAAGGGCAGTCGAATAGTAATAACCACTCGGAATGTTGATCTAGCGGAGAAGTGTGCCACAGCCTCACTGGTGTACCACCTTGATTTCTTGCAGATGAACGATGCCATAACATTGCTACTGAGAAAAACAAATAAAAATCATGAAGACATGGAATCAAATAAAAATATGCAAAAGATGGTTGAACGAATTGTAAATAAATGTGGTCGTCTACCATTAGCAATACTTACAATAGGAGCTGTGCTTGCAACTAAACATGTGTCAGAATGGGAGAAATTCTATGAACAACTTCCTTCAGAACTAGAAATAAACCCAAGCCTGGAAGCTTTGAGGAGAATGGTGACCCTAGGTTACAACCACCTACCATCCCATCTGAAACCATGCTTTTTGTATCTAAGTATCTTTCCTGAGGATTTTGAAATCAAAAGGAATCGTCTAGTAGGTAGATGGATAGCAGAAGGGTTTGTTAGACCAAAGGTTGGGATGACGACTAAGGATGTCGGAGAAAGTTACTTTAATGAGCTAATCAACCGAAGTATGATTCAACGATCAAGAGTGGGCATAGCAGGAAAAATTAAGACTTGTCGAATCCATGATATCATGCGTGATATCACAGTTTCAATCTCGAGACAGGAAAATTTTGTATTATTACCAATGGGAGATGGCTCTGATTTAGTTCAGGAAAACACTCGCCACATAGCATTCCATGGGAGTATGTCCTGCAAAACAGGATTGGATTGGAGCATTATTCGATCATTAGCTATTTTTGGTGACAGACCCAAGAGTCTAGCACATGCAGTTTGTCCAGATCAATTGAGGATGTTACGGGTCTTGGATCTTGAAGATGTGACATTCTTAATCACTCAAAAAGATTTCGACCGTATTGCATTGTTGTGCCACTTGAAATACTTGAGTATTGGATATTCGTCATCCATATATTCACTTCCCAGATCCATTGGTAAACTACAGGGCCTACAAACTTTGAACATGCCGAGCACATACATTGCAGCACTACCAAGTGAGATCAGTAAACTCCAATGTCTGCATACTCTTCGTTGTATAGGACAGTTTCCTTATGACAACTTTAGTCTAAACCACCCAATGAAGTGCATAACTAACACAATATGCCTGCCTAAAGTATTCACACCTTTAGTTAGTCGCGATGATCGTGCAAAACAAATTGCTGAATTGCACATGGCCACCAAAAGTTGCTGGTCTGAATCATTCGGTGTGAAGGTACCCAAAGGAATAGGTAAGTTGCGAGACTTGCAGGTTCTAGAGTATGTAGATATCAGGCGGACCAGTAGTAGAGCAATCAAAGAGCTGGGGCAGTTAAGCAAGTTGAGGAAATTAGGTGTGATAACAAAAGGCTCGACAAAGGAAAAATGTAAGATACTTTATGCAGCCATTGAGAAGCTCTCTTCCCTCCAATCTCTCTATGTGAATGCTGCGTTATTATCAGATATTGAAACACTTGAGTGCCTAGATTCTATTTCATCTCCTCCTCCCCTACTGTGGACACTCGTGTTGATTGGAAGTCTTGAAGAGATGCCTAACTGGATTGAGCAGCTCACTCACCTGAAGAAGTTCTACTTATTAAGCAGCAAACTAAAGGAAGGTAAAACCATGCTGATACTTGGGGCATTGCCCAACCTCATGGTCCTTTATCTTTATTGGAATGCTTACCTTGGGGAGAAGCTAGTATTCAAAACGGGAGCATTCCCAAATCTTAGAACACTTCATATTTACAAATCGGATCAGCTAAGAGAGATGAGATTTGAGGATGGCAGCTCACCCCTGTTGGAAAAGATAGAAATCTCTTGCTGCAGGTTGGAATCAGGGATTATTGGTATCATTCACCTTCCAAGGCTCAAGGAGATTTCACTTGAATACAAAAGTAAAGTGGCTAGGCTTGGTCAGCTGGAGGGAGAAGTGAACACACACCCAAATCGCCCCGTGCTGCGAATGGACAGTGACCGAAGGGATCACGACCTGGGGGCTGAAGCCGAAGGATCTTCTATAGAAGTGCAAACAGCAGATCCTGTTCCTGATGCCCAAGGATCAGTCACTGTAGCAGTGGAAGCAACGGATCCCCTTCCCGAGCAGGAGGGAGAGAGCTCGCAGTCGCAGGTGATCACGTTGACGACGAACGATAGGTCAGTCACTCCCTACATGGCAGCTTAATTAACTTGTTTCTAATTCTCTTCTTGTTCAGTATTAGCCATCAGGTGAGGGCGATGATTTCAACTCACTTTTCATCTCTCTCGTTTTCTTAACCTGACA

>Pi9-Type12

GCTTGCATATGACGTCATGTAGATAGAGATGGCCAATATAATGCGCTGGAAAGTCCAAAGTGAGGATGCAAAACATCTTATAGTGGGTAGTGGAGCCATGCAAGGACCTGGTCTAAAGCGCACCTAAACCGTAATGTGGACTGCCATTATAGTTAAAGTTAGGGGGAATATGATTCTCTTCATGTGCACCTAAACCGTAATATGCAGTGAAACGAACGCTATGATATGATGATAAGCTTAATTCCTCTCTCTGCTCAGACTGTTCAGTGCAAAAGCTACCAACGAGAGCTTGTCTCCTTGTGCGGTCGTGAGCTTGCTTGTGCTAAGCTTGAAGGGAGAGTCGAACGAATCCATGGCGGAGACGGTGCTGAGCATGGCGAGGTCGCTGGTGGGCAGTGCCATCAGCAAGGCCGCCTCTGCCGCTGCCAATGAGACGAGCCTCCTGCTCGGCGTCGAGAAGGACATCTGGTACGTACTGCACTGCTCTCGTTTATCCTAGCAAGTTCTTAGGCTCTTAATCTCGAAATTGAGGAACACCATGAAACACTAAAAGAGAGCTCGAAGACTAGGAAAGAAAACTAGAAGACTAAGCTTTGAAAGTCTTCTAAATCCAAGCATCTCGACATTGATCATCCTTGTGCAACATCATCCCTTCCTATTGCTTCACCAGAATCGGTGTCCCTTGTGGAGATCTCTGTCGTAGCGTCAAGGGGAGAATCCGAGAAGCAGAACTAGTCCGCGCTGCCTTCGCTACGCCATCTCCGCCATAGAGGATCTCATCCACGAAACATCCACCATCCAAACGGGAAACTGTTTTAAACACTCGGGTGGATATTCACCCGTTTCTTGCATGTCATCTAAATGGTTATGAAAAATTTTCAAAAAAAAAACATGATAGGTTAATATATAATATATCATCTCACAAATATGCAAGTTCAAATTCAACTTTTATAAGTTGTAAGTATAACAGGACGTTCATTTCACAAATATGCAAGTTTAAATTTAACTTTTACAAATTGTAAGTGTAACAGGACGTCCATCGGATAGATTAATATCCATCTCCCCATCCAAACCCGTTGTTGCACCATCTGTCGAATCCGGCTGTGGACGCTCGGAGGCAAGAGCTAGCTCACCCGTCCCACACACACACCCAACGACGTCACAAGCGCCTCCGAACAACGCCAACTGATAACTTGGCAGCTCCTACGTGCCGACGTCGCGGTACTTGCCGGCGCTCCTAGCGCACGCACCGTCGAACCACACCGTCACCGACCAACTACCCACCGCCGCCGACTTCTGCCTCATCTGCCATCGTCGCCCTAGCCCAAGTTATCATCGTGGCAATTGCCGAGGCTCCTAAGTGTGCCACGGCCGAGGCAAAGTTCTAACTGAATCAGAGTATCAGACAGCCACCACCGACGCTACTTCTGCTTCATCTGCCATCGCCGTACTAGTTCAAGTTGTCGCTGTGGCAATCATGGGCCCTCCTAGCGTGCCACACAACCGGACAGCCACGACATCCCCCATCACTGTTGTTATTGCCGCGCCCTGACCCCTATCGTCGTCGCTCTTAGCGCGTCGTCGAGCCGACCAGCCACTGTCGTGCAGATGAAAAAAAAACACATATTGGCCTGAGAGATCTGCTTAGTTCCAGTGCAGGTCCAACATGCTGTGAGATGCGGGCGTGCCAGTCAGTTTGATCTTGCAACTGACAAGATATATAAATAGCAGATAAAACAGCCTATCGACTAACAAGCCGATGGAGTAATTCCAGCCGATAGCCGATATTAGCCGATGCCGATTCTAGCCGATGTCGATAGGGTTTTGAACTATCGGCTATATGTCCAATGTAGGTAATGATATAAAGACAATTGGCTGATGATAATAAAATATAAAAATATAATCCAATAGAAACCAATCGGCTAATAATAAGTATTGATCCGATAGTTAAAGCATACATCGGCTAAAAGTCCGATGTCATAAAATCCAATCGATTTAGATAAACAGTGAAACCTTTGTTGCAATCGGCTAAATCCAACTTGTATGTAATCTTCGTAAGCCGATGAACGTCCAGATAACTTATCGGCTAGCACCTCGATAAAACACTAGCATGAACCTATCGGCTTAACAAGATTTATATTATCAACAACAATCTAGTAGGTCGGACCTAACCGATGCAACACGTATTAGATATGATAATCTAATACTTGATGAGCCAATAAATCTGTCTAATGTGATGGATATAACAAATCTATTTATAAAAGCATTGCGATTGTAGAGATATATCGGCTAAGACAGAATATCAGACCTAACTAAACCGATGCGTCTCTAAACACAATGCAATTAATTAGAGATATAATTGAGATATCAGCTAGGCAAATATATCAATCAAACTAGAGCGATCCAAGAGATCGGAGCAATGCAGCCTTGAACAACACCAATGTAGCCGATGGATTCACCAGGGCCGACGGAACGTAGGACTTACCCCTTCCCTGAAGATCGGGCTGAACCAATGCAGTCCCACGTCAGGTGCCAAATTCCGCCGGTTGATAAGTAAAACCTCAGAAAAGAGGATGACGATGCGCCGAGAGTAGTATTGATCGAGAGATAAATTGCAATGACCCTGGATGTACATATTTGTACCCATGGGTAGATATTAGTTCTTGTAGGACAAGAAAGAAACTTTCCTAAAGATAAAATGAAAACATAAAGTCTTTATTGGATACTAAACACACTTTCCTAAAGATAAAAGGAAACTAAACCCTGCCTAATTAATAGATAAACTGCCATGTCGTATCCTCCTTGAACTCGGACTCTTTTAGATAAGCTTCCTTTAACTAATCTTTACCCGAATCCATCAAGAATACAAATGTTGGCATTGATAGTTTTCATCGGTCAATTCTAGGACTTTGAAGCCGATACTGACTCTAAGCCGATGACTACTTTGGGCTTACCAAATTTTGTTGTTAATATGTCGCGACCACCATCACCGGCCAGCCACCCTGATCATTGTTGTTGACTCAGCATTCGCCAGGCTGAGCAGTCCACATACATGCCGCCATCTCCATGGCAGTGTCGTTGCCGCCCCTTTCTCCTAGAGCCGCCGCAGCGCTCTTCGACACACCTACTGCATCGTCGAGCAGTCGTGCTACCACCTCCTCCATCGACCATAGCCGCCTCTTCTGCTGCACCGGATCCACCCACACCAACCACCAGATACAGTCAAGCCCTCATTCCTGGATCCCATATCCATCCATGCCACTACTGTGCTGCCCAGTCCAAGGAATGGAGCGAAGGAGGAAGCCCCGCCGCTGCCCTCCCGGCGGCCACATGCACTCCAGTGCCTTGCTCCGACGGCAGCGAGGTTGGAAAATGGGTGGCAGCGGCTAGGGTTTATCTGGGGAGAAGGAAAAGGAGAGGGAGGGGGGGGGGAGGGTCCACTTCCAGCTTAATTAGCCTAGATCTTATTGACAAATCAGTTGCTGGGTGCACAAACATGTTATTTTTTTTGCATGACCAATCTTGAACACTTAGGTATGTTAGTTGAGTGGACACTGGTCTATCTGAAACATCTCTTCACATGGAGGCTGCGAATGAGTTTTCTTTTTGAGAGACCAAAGTTTCGTTGTATGTTAAGTGATAAAGCCTTGGTAAGAAATGCTACCACAAACGAACTAATAACTCCAAACGTAAAGTGGAGGAACCCGTATGGGTGACTCGAGTGGCGACAAACTCTAGCACCTCCACCTCCTTGGACGGGCTGCGGCGGTGCTTTCGGCATCCCAGTCTTCTTGGAGGCATCATCTAGAATTAAGGTCTTGTTATTGCTTAGCATGCCTTAGGGCACGTCCAGTGTTTAGTTCGACTAAAACTTCCATGAAAGCCAAACAAAAGTTCTGTTTGACCACCACAGTGTAAAAATCGATTGTGGGACCCATGCAAAAAAATCACAATCTCAGCTGCCTATGCTCTCCTCCTGGACCTGATAGCCGTGCACAACAAATATTTTTTTAAACTGGATGTGTTCGGCTTCTCTTTAAAGATCGTTTTTTCCTCTGACACTTACCAACCGGCTTTCACAGTGTGGTCAGTTCTTTTTTTTTTTACGCAAAGTTTGATTTTAGTCAGACACGGGAGGATCTGTTAAGCAGGCTTGGAAATTTCGGACCCCTCCAATACAATATTATTTTAGCCAAAATTTCTAATTTTTTAATTTTTCATGAATTTTGGTAATATTTGTTCTAATTTAACTAAATTTTGTTCAAAATTTCGGTCTATCAGTGACCTCCGATCAAATCAGTTAAACCGAGAAAATAAACCATGCTCTTAAGAGAGTTTGGTATGGTTCAATATCAAAACTTATAGTCTTGCAATTTTTTCTACCCTTTATCTTTTTCCCTGACTATTTAGTATGGATCGTTTAAAAAAAAGAAAGCCCATTGGTGACCAAGGGCTTGTTTGATTCAAGACCATCCCTAGCCTTACCAACCTTTTGGCAATGGCAAAAATTGGTTGTTGCCAAAAATATTGGCACAAATTGGCTAAGCCTATGATTGGTTTCTACCAAAGTTGAATTTTGGCATTCAATCAAGCCAAATAATTTGGCAATAACATTTTCTTATCTATGGATATAACATATGGCAAATATTTTGGCATTACCATTTTCTTTTTGCCAAACATGTTATTCCTTTTGAATGACCAATCTTGACACCTTATGTATGTTAGTAGTGGAATCGACACTATTCTATCTAAAACATCTCTTTACATAGAGGCCGCTAATAATTTTTCTTTGAGATAACCAAATTTTCCTTACAAGTTAAGCAACAAAGCCCATTGGTAAGATATGCTACGACAAATGAACTAATAACTCCAAACGTAAAGCGGAGGATCCCGCATTTCCCACGTGGGTGACTCGAGCGGTGACAAACCCTAGTACCTCCACCCCCTTGGGTGGGTTGTGGTGGCACTTTCGGCACCGTATTTTCCTTGGACGGATCATTTAGAAAGTCCTATTATTGCCTAGTATGCCTTGACAGTTTAGGCAACACTCTTGGATGGTGGTGTCCTTTGCCCTGGTGATCTAGTAGCCCATGGATGTTTAGTTATTTGGACATGGTGTTGGATGGTGCGCTCGTGGGCCTGTTGTAGGTCTGGTGCCAACCAGTCATGCTTAGAAATAGCCGGATAGGTGCACAGTGCTAGTTCTTTACTTGGTGGTTTGTGCAGCGCTATCGACATGTGGTGGTGTGCTTTTTCTTTGTCCGGATAATAATCTCATAGGGCTATACTCTTGTTATTTTGCTGCTATATTATTATGATAACTTGCTATGGTTCGTTTTTTCTTTTTTTGGAAAAACACCTAGTTGATCAAGGGCTTGTTTGGTTCAAGTGCATTCCTAATCTTACCTTTTCTTTTTTTTTTTCAATGGCAAGAATTGTTCATTGCAAAAAAAAAAAGAGATAAAAATTGGCTAGGCTTACGTTTTGGTTCTTACCAAAGTTGTACTTTGAGACCAAATATATGGCAAAATTTTGGCATAACCTTTTTTTTTTTTGCTTGGTTGAGCTTGGTACAAACCAATCAGTCACAAAATAGACTGTCATGAATCACGCCTACTAAATTCCTTTGAACCGAACTAGAATATATTTGCTCTTAAAAGATTTCTTGATTTCAATTGGTACCATTTACTAGTAGAAACTTAAATTTAAATTTTAAAAACAAAATCATAATATTGTTGTTATGGAAATTTTAGTCATTTTAGTACTTTTGTAATATATGAGTTGGGTTATACTTGAGATATCCTAAATTGCTTTAAGATGAACAATTGCTAGGTATATCAAAGATGAGCTAAAAACAATGCAGGCATTCCTTAGAGCTGCTGAAGTTATGAAAAAGAAAGATGAACTATTAAAGGTTTGGGCAGAGCAAATACGTGACCTATCGTATGACATTGAAGATTCCCTTGATGAATTTAAAGTCCATATTGAAAGCCAAACCCTATTTCGTCAGTTGGTGAAACTTAGAGAGCGCCACCGGATCGCTATCCGTATCCACAACCTCAAATCAAGAGTTGAAGAAGTGAGTAGCAGGAACACACGCTACAATTTAGTCGAGCCTATTTCCTCCGGCACAGAGGATGACATGGATTCCTATGCAGAAGACATTCGCAATCAATCAGCTCGAAATGTGGATGAAGCTGAGCTTGTTGGGTTTTCTGACTCCAAGAAAAGGCTGCTTGAAATGATCGATACCAATGCTAATGATGGTCCGGCCAAGGTAATCTGTGTTGTTGGGATGGGTGGTTTAGGCAAGACAGCTCTTTCGAGGAAGATCTTTGAAAGCGAAGAAGACATTAGGAAGAACTTCCCTTGCATTGCTTGGATTACAGTGTCACAATCATTTCACAGGATTGAGCTACTTAAAGATATGATACGCCAACTTCTTGGCCCCAGTTCTCTGGATCAACTCTTGCAAGAATTGCAAGGGAAGGTGGTGGTGCAAGTACATCATCTTTCTGAGTACCTGATAGAAGAGCTCAAGGAGAAGAGGTACTTTGTTATTCTAGATGATCTATGGATTTTACATGATTGGAATTGGATAAATGAAATTGCATTTCCTAAGAACAATAAGAAGGGCAGTCGAATAGTAATAACCACTCGGAATGTTGATCTAGCGGAGAAGTGTGCCACAGCCTCACTGGTGTACCACCTTGATTTCTTGCAGATGAACGATGCCATAACATTGCTACTGAGAAAAACAAATAAAAATCATGAAGACATGGAATCAAATAAAAATATGCAAAAGATGGTTGAACGAATTGTAAATAAATGTGGTCGTCTACCATTAGCAATACTTACAATAGGAGCTGTGCTTGCAACTAAACATGTGTCAGAATGGGAGAAATTCTATGAACAACTTCCTTCAGAACTAGAAATAAACCCAAGCCTGGAAGCTTTGAGGAGAATGGTGACCCTAGGTTACAACCACCTACCATCCCATCTGAAACCATGCTTTTTGTATCTAAGTATCTTTCCTGAGGATTTTGAAATCAAAAGGAATCGTCTAGTAGGTAGATGGATAGCAGAAGGGTTTGTTAGACCAAAGGTTGGGATGACGACTAAGGATGTCGGAGAAAGTTACTTTAATGAGCTAATCAACCGAAGTATGATTCAACGATCAAGAGTGGGCATAGCAGGAATAATTAAGACTTGTCGAATCCATGATATCATCCGTGATATCACAGTTTCAATCTCGAGACAGGAAAATTTTGTATTATTACCAATGGGAGATGGCTCTGATTTAGTTCAGGAAAACACTCGCCACATAGCATTCCATGGGAGTATGTCCTGCAAAACAGGATTGGATTGGAGCATTATTCGATCATTAGCTATTTTTGGTGACAGACCCAAGAGTCTAGCACATGCAGTTTGTCTAGATCAATTGAGGATGTTACGGGTCTTGGATCTTGAAGATGTGACATTCTTAATCACTCAAAAAGATTTCGACCGTATTGCATTGTTGTGCCACTTGAAATACTTGAGTATTGGATATTCGTCATCCATATATTCACTTCCCAGATCCATTGGTAAACTACAGGGCCTACAAACTTTGAACATGCTGAGAACATACATTGCAGCACTACCAAGTGAGATCAGTAAACTCCAATGTCTGCATACTCTTCGTTGTAGTAGAAAGTTTGTTTATGACAACTTTAGTCTAAACCACCCAATGAAGTGCATAACTAACACAATATGCCTGCCTAAAGTATTCACACCTTTAGTTAGTCGCGATGATCGTGCAAAACAAATTGCTGAATTGCACATGGCCACCAAAAGTTGCTGGTCTGAATCATTCGGTGTGAAGGTACCCAAAGGAATAGGTAAGTTGCGAGACTTGCAGGTTCTAGAGTATGTAGATATCAGGCGGACCAGTAGTAGAGCAATCAAAGAGCTGGGGCAGTTAAGCAAGTTGAGGAAATTAGGTGTGATAACAAAAGGCTCGACAAAGGAAAAATGTAAGATACTTTATGCAGCCATTGAGAAGCTCTCTTCCCTCCAATCTCTCTATGTGAATGCTGCGTTATTATCAGATATTGAAACACTTGAGTGCCTAGATTCTATTTCATCTCCTCCTCCCCTACTGTGGACACTCGGGTTGAATGGAAGTCTTGAAGAGATGCCTAACTGGATTGAGCAGCTCACTCACCTGAAGAAGATCTACTTATTGAGGAGCAAACTAAAGGAAGGTAAAACCATGCTGATACTTGGGGCATTGCCCAACCTCATGGTCCTTTATCTTTATTGGAATGCTTACCTTGGGGAGAAGCTAGTATTCAAAACGGGAGCATTCCCAAATCTTAGAACACTTCGTATTTACGAATTGGATCAGCTAAGAGAGATGAGATTTGAGGATGGCAGCTCACCCCTGTTGGAAAAGATAGAAATCTCTTGCTGCAGGTTGGAATCAGGGATTATTGGTATCATTCACCTTCCAAGGCTCAAGGAGATTTCACTTGAATACAAAAGTAAAGTGGCTAGGCTTGGTCAGCTGGAGGGAGAAGTGAACACACACCCAAATCGCCCCGTGCTGCGAATGGACAGTGACCGAAGGGATCACGACCTGGGGGCTGAAGCCGAAGGATCTTCTATAGAAGTGCAAACAGCAGATCCTGTTCCTGATGCCGAAGGATCAGTCACTGTAGCAGTGGAAGCAACGGATCCCCTTCCCGAGCAGGAGGGAGAGAGCTCGCAGTCGCAGGTGATCACGTTGACGACGAACGATAGGTCAGTCACTCCCTACATGGCAGCTTAATTAACTTGTTTCTAATTCTCTTCTTGTTCAGTATTAGCCATCAGGTGAGGGCGATGATTTCAACTCACTTTTCATCTCTCTCGTTTTCTTAACCTGACA

>Pi9-Type13

GCTTGCATATGACGTCATGTAGATAGAGATGGCCAATATAATGCGCTGGAAAGTCCAAAGTGAGGATGCAAAACATCTTATAGTGGGTAGTGGAGCCATGCAAGGACCTGGTCTAAAGCGCACCTAAACCGTAATGTGGACTGCCATTATAGTTAAAGTTAGGGGGAATATGATTCTCTTCATGTGCACCTAAACCGTAATATGCAGTGAAACGAACGCTATGATATGATGATAAGCTTAATTCCTCTCTCTGCTCAGACTGTTCAGTGCAAAAGCTACCAACGAGGCTTTCTCCTTGTGCGGTCGTGAGCTTGCTTGTGCTAAGCTTGAAGGGAGAGTCGAACGAATCCATGGCGGAGACGGTGCTGAGCATGGCGAGGTCGCTGGTGGGCAGTGCCATCAGCAAGGCCGCCTCTGCCGCTGCCAATGAGACGAGCCTCCTGCTCGGCGTCGAGAAGGACATCTGGTACGTACTGCACTGCTCTCGTTTATCCTAGCAAGTTCTTAGGCTCTTAATCTCGAAATTGAGGAACACCATGAAACACTAAAAGAGAGCTCGAAGACTAGGAAAGAAAACTAGAAGACTAAGCTTTGAAAGTCTTCTAAATCCAAGCATCTCGACATTGATCATCCTTGTGCAACATCATCCCTTCCTATTGCTTCACCAGAATCGGTGTCCCTTGTGGAGATCTCTGTCGTAGCGTCAAGGGGAGAATCCGAGAAGCAGAACTAGTCCGCGCTGCCTTCGCTACGCCATCTCCGCCATAGAGGATCTCATCCACGAAACATCCACCATCCAAACGGGAAACTGTTTTAAACACTCGGGTGGATGTTCACCCGTTTCTTGCATGTCATCTAAATGGTTATGAAAAATTTTCAAAAAAAAAACATGATAGGTTAATATATAATATATCATCTCACAAATATGCAAGTTCAAATTCAACTTTTATAAGTTGTAAGTATAACAGGACGTTCATCTCACAAATATGCAAGTTTAAATTTAACTTTTACAAGTTGTAAGTGTAACAGTACGTCCATCGGATAGATTAATATCCATCTCCCCATCCAAACCCGTTGTTGCACCATCTGTCGAATCCGGCTGTGGACGCTCGGAGGCAAGAGCTAGCTCACCCGTCCCACACACACACCCAACGACGTCACAAGCGCCTCCGAACAACGCCAACTGATAACTTGGCAGCTCCTACGTGCCGACGTCGCGGTATTTGCCGGCGCTCCTAGCGCACGCACCGTCGAACCACACCGTCACCGACCAACTACCCACCGCCGCCGACTTCTGCCTCATCTGCCATCGTCGCCCTAGCCCAAGTTATCATCGTGGCAATTGCCGAGGCTCCTAAGTGTGCCACGGCCGAGGCAAAGTTCTAACTGAATCAGACAGCCACCACCGACACTTCTGCTTCATCTGCCATCGCCGTACTAGTTCAAGTTGTCGCTGTGGCAATCATTGGCCCTCCTAGCGTGCCACCCAACCGGACAACCACGACATCCCCCATCACTGTTGTTATTGCCGCGCCCTGACCCCTATCGTCGTCGCTCTTAGCGCGTCGACGAGCCGACCAGCCACTGTCGTGCAGATGAAAAAAAAAACACATATTGGCCTGAGAGATCTGCTTAGTTCCAGTGCAGGTCCAACATGCTGTGAGATGCGGGCGTGCCAGTCAGTTTGATCTTGCAACTGACAAGATATATAAACAGCAGATAAAACAGCCGATCGACTAACAAGCCGATGGAGTAATTCCAGCCGATAGCCGATATTAGCCGATGCCGATTCTAGCCGATGTCGATAGGGTTTTGAACTATCGGCTATATGTCTAATGTAGGCAATGATATAAAGACAATTGGCTGATGATAATAAAATATAAAAATATAATCCAATAGAAACCAATCGGCTAATAATAAATATTGATCCGATGGTTAAAGCATACATCGGCTAAAAGTCCGATGTCATAAAATCCAATCGATTTAGATAAACAGTGAAACCTTTTTTGCAATCGGCTAAATCCAACTTGTATGTAATCTTCGTAAGCCGATGAACGTCCAGATAACTTATCGGTTAGCACCTCGATAAAACACTAGCATGAACCTATCGGCTTAACAAGATTTATATTATCAACAACAATCTAGTAGGTCGGACCTAACCGATGCAACACGGATTAGATATGATAATCTAATACTCGATGAGCCAATAGATCTGTCTAATGTGATGGATATAACAAATCTATTTATAACAGCATTGCGATTGTAGAGATATATCGGCTAAGACAGAATATCAGACCTAACTAAACCGATGCGTCTCTAAACACAATGCAATTAATTAGAGATATAATTGAGATATCAGCTAGGCAAATATATCAACCAAACTGGAGCGATCCAAGAGATCGGAGCAATGCAGCCTTGAACAACACCAATGTAGCCGATGGATTCACCAGGGCCGACGGAACGTAGGACTTACCCCTTCGCTGAAGATCGGGCTGAACCAATGCAGCCCCGCGTCAGGTGCCAAATTCCGCCGGTTGATAAGTAAAACCTCAGAAAAGAGGATGACGATGCGCCGAGAGTAGTATTGATCGAGAGATAAATTGCAATGACCCTGGATGTACATATTTGTACCCATGGGTAGATATTAGTTCTTGTAGGACAAGAAAGAAACTTTCCTAAAGATAAAATGAAAACATAAAGTCTTTATTGGATACTAAATACACTTTCCTAAAGATAAAAGGAAACTAAACCCTGCCTAATTAATAGATAAACTGCCATGTCGTATCCTCCTTGAACTCGGACTCTTTTAGATAAGCTACCTTTAACTAATCTTTACCCGAATCCATCAAGAATACAAATGTTGGCATTGATAGTTTCCATCGGTCAATTCTAGGACTTTGAAGCCGATACTGACTCTAAGCCGATGATTACTTTGTGCTTACCAAATTTTGTTGTTAACATGTCGCAACCACCATCACCGGCCAGCCACCCTGATCATTGTTGTTGACTCAGCATTCGCCAGGCTGAGCAGTCCACATACATGCCGCCATCTCCATGGCACTGTCGTTGCCGCCCCTTTCTCCTAGAGCCGCCGCAGCGCTCTTCGACACACCTACTGCATCGTCGAGCAGTCGTGCTACCACCTCCTCCATCGACCATAGCCGCCTCTTCTGCTGCACCGGATCCACCCACACCAACCACCAGATACAGTCAAGCCCTCATTCCCGGATCCCATATCCATCCATGCCACTACTGTGCTGCCCAGTCCAAGGAATGGAGCGAAGGAGGAAGCCCCGCCGCTGCCCTCCCGGCGGCCACATGCACTCCAGTGCCTTGCTCCGACGGCAGCGAGGTTGGAAAATGGGTGGCAGCGGCTAGGGTTTATCTGGGGAGAAGGAAAAGGAGAGGGAGGGGGGGGGGGTCCACTTCCAGCTTAATTAGCCTAGATCTTATTGACAAATCAGTTGCTGGGTGCACAGACATGTTATTCTTTTTGCATGACCAATCTTGAACACTTAGGTATGTTAGTTGAGTGGACACTGGTCTATCTGAAACATCTCTTCACATGGAGGCTGCGAATGAGTTTTCTTTTTGAGAGACCAAAGTTTCGTTGTATGTTAAGTGATAAAGCCTTGGTAAGAAATGCTACCACAAACGAACTAATAACTCCAAACGTAAAGTGGAGGAACCCGTATGGGTGACTCGAGTGGCGACAAACTCTAGCACCTCCACCTCCTTGGACGGGCTGCGGCGGTGCTTTCGGCATCCCAGTCTTCTTGGAGGCATCATCTAGAATTAAGGTCTTGTTATTGCTTAGCATGCCTTAGGGCACGTCCAGTGTTTAGTTCGACTAAAACTTCCATGAAAGCCAAACAAAAGTTCTGTTTGACCACCACAGTGTAAAAATCGATTGTGGGACCCATGCAAAAAAATCACAATCTCAGCTGCCTATGCTCTCCTCCTGGACCTGATAGCCGTGCACAACAAATATTTTTTTAAACTGGATGTGTTCGGCTTCTCTTTAAAGATCGTTTTTTCCTCTGACACTTACCAACCGGCTTTCACAGTGTGGTCAGTTCTTTTTTTTTTTACGCAAAGTTTGATTTTAGTCAGACACGGGAGGATCTGTTAAGCAGGCTTGGAAATTTCGGACCCCTCCAATACAATATTATTTTAGCCAAAATTTCTAATTTTTTAATTTTTCATGAATTTTGGTAATATTTGTTCTAATTTAACTAAATTTTGTTCAAAATTTCGGTCTATCAGTGACCTCCGATCAAATCAGTTAAACCGAGAAAATAAACCATGCTCTTAAGAGAGTTTGGTATGGTTCAATATCAAAACTTATAGTCTTGCAATTTTTTCTACCCTTTATCTTTTTCCCTGACTATTTAGTATGGATCGTTTAAAAAAAAGAAAGCCCATTGGTGACCAAGGGCTTGTTTGATTCAAGACCATCCCTAGCCTTACCAACCTTTTGGCAATGGCAAAAATTGGTTGTTGCCAAAAATATTGGCACAAATTGGCTAAGCCTATGATTGGTTTCTACCAAAGTTGAATTTTGGCATTCAATCAAGCCAAATAATTTGGCAATAACATTTTCTTATCTATGGATATAACATATGGCAAATATTTTGGCATTACCATTTTCTTTTTGCCAAACATGTTATTCCTTTTGAATGACCAATCTTGACACCTTATGTATGTTAGTAGTGGAATCGACACTATTCTATCTAAAACATCTCTTTACATAGAGGCCGCTAATAATTTTTCTTTGAGATAACCAAATTTTCCTTACAAGTTAAGCAACAAAGCCCATTGGTAAGATATGCTACGACAAATGAACTAATAACTCCAAACGTAAAGCGGAGGATCCCGCATTTCCCACGTGGGTGACTCGAGCGGTGACAAACCCTAGTACCTCCACCCCCTTGGGTGGGTTGTGGTGGCACTTTCGGCACCGTATTTTCCTTGGACGGATCATTTAGAAAGTCCTATTATTGCCTAGTATGCCTTGACAGTTTAGGCAACACTCTTGGATGGTGGTGTCCTTTGCCCTGGTGATCTAGTAGCCCATGGATGTTTAGTTATTTGGACATGGTGTTGGATGGTGCGCTCGTGGGCCTGTTGTAGGTCTGGTGCCAACCAGTCATGCTTAGAAATAGCCGGATAGGTGCACAGTGCTAGTTCTTTACTTGGTGGTTTGTGCAGCGCTATCGACATGTGGTGGTGTGCTTTTTCTTTGTCCGGATAATAATCTCATAGGGCTATACTCTTGTTATTTTGCTGCTATATTATTATGATAACTTGGTATGGTTCGTTTTTTCTTTTTTTGGAAAAACACCTAGTTGATCAAGGGCTTGTTTGGTTCAAGTGCATTCCTAATCTTACCTTTTCTTTTTTTTTTCAATGGCAAGAATTGTTCATTGCAAAAAAAAAAAGAGATAAAAATTGGCTAGGCTTACGTTTTGGTTCTTACCAAAGTTGTACTTTGAGACCAAATATATGGCAAAATTTTGGCATAACCTTTTTTTTTTTTGCTTGGTTGAGCTTGGTACAAACCAATCAGTCACAAAATAGACTGTCATGAATCACGCCTACTAAATTCCTTTGAACCGAACTAGAATATATTTGCTCTTAAAAGATTTCTTGATTTCAATTGGTACCATTTACTAGTAGAAACTTAAATTTAAATTTTAAAAACAAAATCATAATATTGTTGTTATGGAAATTTTAGTCATTTTAGTACTTTTGTAATATATGAGTTGGGTTATACTTGAGATATCCTAAATTGCTTTAAGATGAACAATTGCTAGGTATATCAAAGATGAGCTAAAAACAATGCAGGCATTCCTTAGAGCTGCTGAAGTTATGAAAAAGAAAGATGAACTATTAAAGGTTTGGGCAGAGCAAATACGTGACCTGTCGTATGACATTGAAGATTCCCTTGATGAATTTAAAGTCCATATTGAAAGCCAAACCCTATTTCGTCAGTTGGTGAAACTTAGAGAGCGCCACCGGATCGCTATCCGTATCCACAACCTCAAATCAAGAGTTGAAGAAGTGAGTAGCAGGAACACACGCTACAATTTAGTCGAGCCTATTTCCTCCGGCACAGAGGATGACATGGATTCCTATGCAGAAGACATTCGCAATCAATCAGCTCGAAATGTGGATGAAGCTGAGCTTGTTGGGTTTTCTGACTCCAAGAAAAGGCTGCTTGAAATGATCGATACCAATGCTAATGATGGTCCGGCCAAGGTAATCTGTGTTGTTGGGATGGGTGGTTTAGGCAAGACAGCTCTTTCGAGGAAGATCTTTGAAAGCGAAGAAGACATTAGGAAGAACTTCCCTTGCATTGCTTGGATTACAGTGTCACAATCATTTCACAGGATTGAGCTACTTAAAGATATGATACGCCAACTTCTTGGCCCCAGTTCTCTGGATCAACTCTTGCAAGAATTGCAAGGGAAGGTGGTGGTGCAAGTACATCATCTTTCTGAGTACCTGATAGAAGAGCTCAAGGAGAAGAGGTACTTTGTTATTCTAGATGATCTATGGATTTTACATGATTGGAATTGGATAAATGAAATTGCATTTCCTAAGAACAATAAGAAGGGCAGTCGAATAGTAATAACCACTCGGAATGTTGATCTAGCGGAGAAGTGTGCCACAGCCTCACTGGTGTACCACCTTGATTTCTTGCAGATGAACGATGCCATAACATTGCTACTGAGAAAAACAAATAAAAATCATGAAGACATGGAATCAAATAAAAATATGCAAAAGATGGTTGAACGAATTGTAAATAAATGTGGTCGTCTACCATTAGCAATACTTACAATAGGAGCTGTGCTTGCAACTAAACATGTGTCAGAATGGGAGAAATTCTATGAACAACTTCCTTCAGAACTAGAAATAAACCCAAGCCTGGAAGCTTTGAGGAGAATGGTGACCCTAGGTTACAACCACCTACCATCCCATCTGAAACCATGCTTTTTGTATCTAAGTATCTTTCCTGAGGATTTTGAAATCAAAAGGAATCGTCTAGTAGGTAGATGGATAGCAGAAGGGTTTGTTAGACCAAAGGTTGGGATGACGACTAAGGATGTCGGAGAAAGTTACTTTAATGAGCTAATCAACCGAAGTATGATTCAACGATCAAGAGTGGGCATAGCAGGAAAAATTAAGACTTGTCGAATCCATGATATCATCCGTGATATCACAGTTTCAATCTCGAGACAGGAAAATTTTGTATTATTACCAATGGGAGATGGCTCTGATTTAGTTCAGGAAAACACTCGCCACATAGCATTCCATGGGAGTATGTCCTGCAAAACAGGATTGGATTGGAGCATTATTCGATCATTAGCTATTTTTGGTGACAGACCCAAGAGTCTAGCACATGCAGTTTGTCCAGATCAATTGAGGATGTTACGGGTCTTGGATCTTGAAGATGTGACATTCTTAATCACTCAAAAAGATTTCGACCGTATTGCATTGTTGTGCCACTTGAAATACTTGAGTATTGGATATTCGTCATCCATATATTCACTTCCCAGATCCATTGGTAAACTACAGGGCCTACAAACTTTGAACATGCCGAGCACATACATTGCAGCACTACCAAGTGAGATCAGTAAACTCCAATGTCTGCATACTCTTCGTTGTAGTAGAAAGTTTGTTTATGACAACTTTAGTCTAAACCACCCAATGAAGTGCATAACTAACACAATATGCCTGCCTAAAGTATTCACACCTTTAGTTAGTCGCGATGATCGTGCAATACAAATTGCTGAATTGCACATGGCCACCAAAAGTTGCTGGTCTGAATCATTCGGTGTGAAGGTACCCAAAGGAATAGGTAAGTTGCGAGACTTACAGGTTCTAGAGTATGTAGATATCAGGCGGACCAGTAGTAGAGCAATCAAAGAGCTGGGGCAGTTAAGCAAGCTGAGGAAATTAGGTGTGACAACAAACGGGTCGACAAAGGAAAAATGTAAGATACTTTATGCAGCCATTGAGAAGCTCTCTTCCCTCCAATCTCTCCATGTGGATGCTGTGTTATTCTCAGGTATTATTGGAACACTTGAGTGCCTAGATTCTATTTCATCTCCTCCTCCCCTACTGAGGACACTCGGGTTGAATGGAATTCTTGAAGAGATGCCTAACTGGATTGAGCAGCTCACTCACCTGAAGAAGTTCTACTTATTAAGCAGCAAACTAAAGGAAGGTAAAACCATGCTGATACTTGGGGCATTGCCCAACCTCATGGTCCTTTATCTTTATTGGAATGCTTACCTTGGGGAGAAGCTAGTATTCAAAACGGGAGCATTCCCAAATCTTAGAACACTTCATATTTACGAATCGGATCAGCTAAGAGAGATGAGATTTGAGGATGGCAGCTCACCCCTGTTGGAAAAGATAGAAATATTCAGGTGCAGGTTGGAATCAGGGATTATTGGTATCATTCACCTTCCAAGGCTCAAGGAGATTTCACTTGAATACAAAAGTAAAGTGGCTAGGCTTGGTCAGCTGGAGGGAGAAGTGAGCACACACCCAAATCGCCCCGTGCTGCGAATGGACAGTGACCGAAGGGATCACGACCTGGGGGCTGAAGCCGAAGGATCTTCTATAGAAGTGCAAACAGCAGATCCTGTTCCTGATGCCCAAGGATCAGTCACTGTAGCAGTGGAAGCAACGGATCCCCTTCCCGAGCAGGAGGGAGAGAGCTCGCAGTCGCAGGTGATCATGTTGACGACGAACGATAGGTCAGTCACTCCCTACATGGCAGCTTAATTAACTTGTTTCTAATTCTCTTCTTGTTCAGTATTAGCCATCAGGTGAGGGCGATGATTTCAACTCACTTTTCATCTCTCTCGTTTTCTTAACCTGACA
