# Supplementary material for: Identification of Novel Alleles of the Rice Blast-Resistance Gene Pi9 through Sequence-Based Allele Mining
Source: Rice (N Y). 2020 Dec 7;13:80. doi: 10.1186/s12284-020-00442-z (PMC7721961; doi:10.1186/s12284-020-00442-z)
Supplement: Supplementary file 5 — Additional file 5: Supplementary Table 5. Sequence of Pi9 allele proteins [file 12284_2020_442_MOESM5_ESM.doc]

>Pi9

MAETVLSMARSLVGSAISKAASAAANETSLLLGVEKDIWYIKDELKTMQAFLRAAEVMKK

KDELLKVWAEQIRDLSYDIEDSLDEFKVHIESQTLFRQLVKLRERHRIAIRIHNLKSRVE

EVSSRNTRYNLVEPISSGTEDDMDSYAEDIRNQSARNVDEAELVGFSDSKKRLLEMIDTN

ANDGPAKVICVVGMGGLGKTALSRKIFESEEDIRKNFPCIAWITVSQSFHRIELLKDMIR

QLLGPSSLDQLLQELQGKVVVQVHHLSEYLIEELKEKRYFVILDDLWILHDWNWINEIAF

PKNNKKGSRIVITTRNVDLAEKCATASLVYHLDFLQMNDAITLLLRKTNKNHEDMESNKN

MQKMVERIVNKCGRLPLAILTIGAVLATKHVSEWEKFYEQLPSELEINPSLEALRRMVTL

GYNHLPSHLKPCFLYLSIFPEDFEIKRNRLVGRWIAEGFVRPKVGMTTKDVGESYFNELI

NRSMIQRSRVGIAGKIKTCRIHDIIRDITVSISRQENFVLLPMGDGSDLVQENTRHIAFH

GSMSCKTGLDWSIIRSLAIFGDRPKSLAHAVCLDQLRMLRVLDLEDVTFLITQKDFDRIA

LLCHLKYLSIGYSSSIYSLPRSIGKLQGLQTLNMLRTYIAALPSEISKLQCLHTLRCSRK

FVYDNFSLNHPMKCITNTICLPKVFTPLVSRDDRAKQIAELHMATKSCWSESFGVKVPKG

IGKLRDLQVLEYVDIRRTSSRAIKELGHLSKLRKLGVITKGSTKEKCKILYAAIEKLSSL

QSLYVNAALLSDIETLECLDSISSPPPLLRTLGLNGSLEEMPNWIEQLTHLKKIYLLRSK

LKEGKTMLILGALPNLMVLYLYWNAYLGEKLVFKTGAFPNLRTLRIYELDQLREMRFEDG

SSPLLEKIEISCCRLESGIIGIIHLPRLKEISLEYKSKVARLGQLEGEVNTHPNRPVLRM

DSDRRDHDLGAEAEGSSIEVQTADPVPDAEGSVTVAVEATDPLPEQEGESSQSQVITLTT

NDSEEIGTAQAG*

>Pi9-Type01

MAETVLSMARSLVGSAISKAASAAANETSLLLGVEKDIWYIKDELKTMQAFLRAAEVMKK

KDELLKVWAEQIRDLSYDIEDSLDEFKVHIESQTLFRQLVKLRERHRIAIRIHNLKSRVE

EVSSRNTRYNLVEPISSGTEDDMDSYAEDIRNQSARNVDEAELVGFSDSKKRLLEMIDTN

ANDGPAKVICVVGMGGLGKTALSRKIFESEEDIRKNFPCNAWITVSQSFHRIELLKDMIR

QLLGPSSLDQLLQELQGKVVVQVHHLSEYLIEELKEKRYFVVLDDLWILHDWNWINEIAF

PKNNKKGSRIVITTRNVDLAEKCATASLVYHLDFLQMNDAITLLLRKTNKNHEDMESNKN

MQKMVERIVNKCGRLPLAILTIGAVLATKHVSEWEKFYEQLPSELEINPSLEALRRMVTL

GYNHLPSHLKPCFLYLSIFPEDFEIKRNRLVGRWIAEGFVRPKVGMTTKDVGESYFNELI

NRSMIQRSRVGIAGIIKTCRIHDIIRDITVSISRQENFVLLPMGDGSDLVQENTRHIAFH

GSMSCKTGLDWSIIRSLAIFGDRPKSLAHAVCPDQLRMLRVLDLEDVTFLITQKDFDRIA

LLCHLKYLSIGYSSSIYSLPRSIGKLQGLQTLNMSSTYIAALPSEISKLQCLHTLRCSRK

FVYDNFSLNHPMKCITNTICLPKVFTPLVSRDDRAKQIAELHMATKSCWSESFGVKVPKG

IGKLRDLQVLEYVDIRRTSSRAIKELGQLSKLRKLGVITKGSTKEKCKILYAAIEKLSSL

QSLYVNAALFSGIGTLQCIDSISSPPPLLRTLGLNGSLEEMPNWIEQLTHLKKIYLLRSK

LKEGKTMLILGALPNLMVLDLYHNSYLGEKLVFKTGAFPNLRTLWIYELDQLREIRFEDG

SSPQLEKIEIRFCRLESGIIGIIHLPRLKEISRGYESKVAGLAQLEGEVNAHPNRPVLLM

YSDGRYHDLGAEAEGSSIEVQTADPVPDAEGSVTVAVEATDPLLEQEGESSQSQVITLTT

NDSEEIGTAQAG*

>Pi9-Type02

MAETVLSMARSLVGSAISKAASAAANETSLLLGVEKDIWYIKDELKTMQAFLRAAEVMKK

KDELLKVWAEQIRDLSYDIEDSLDEFKVHIESQTLFRQLVKLRERHRIAIRIHNLKSRVE

EVSSRNTRYNLVEPISSGTEDDMDSYAEDIRNQSARNVDEAELVGFSDSKKRLLEMIDTN

ANDGPAKVICVVGMGGLGKTALSRKIFESEEDIRKNFPCNAWITVSQSFHRIELLKDMIR

QLLGPSSLDQLLQELQGKVVVQVHHLSEYLIEELKEKRYFVVLDDLWILHDWNWINEIAF

PKNNKKGSRIVITTRNVDLAEKCATASLVYHLDFLQMNDAITLLLRKTNKNHEDMESNKN

MQKMVERIVNKCGRLPLAILTIGAVLATKQVSEWEKFYEQLPSELEINPSLEALRRMVTL

GYNHLPSHLKPCFLYLSIFPEDFEIKRNRLVGRWIAEGFVGPKVGMTIKDVGKSYFYELI

NRSMIQRSRVGIEGKIKSCRVHDIMRDITVSISRQENFVLLPMDDGSDLVQENTRHIAFH

GSMSCKTGLDWSIIRSLTIFGDRPKSLAHAVCSDQLRMLRVLDLEDVKFLSTQKDFNNIA

LLRHLKYLSIGNSSCIYSLPRSIGKLQGLQTLNMSSTYIAALPSEISKLQCLHTLRCIRE

LDFDKFSLNRPMKCITNTICLPKVFTPLVSRDNRAKQIAEFHMATKSFWSESFGVKVPKG

IGKLRDLQVLEYVDIRRTSSRAIKELGQLSKLRKLGVITKGSTKEKCKILYAAIEKLSSL

QSLYVNAALLSDIETLECLDSISSPPPLLRTLGLNGSLEEMPNWIEQLTHLKKFYLWRSK

LKEGKTMLILGALPNLMFLSLYHNSYLGEKLVFKTGAFPNLRTLWIYELDQLREIRFEDG

SSPLLEKIEIGECRLESGIIGIIHLPRLKEISLRYESKVAGLAQLEGEVNAHPNRPVLLM

YSDRRYHDLGAEAEGSSIEVQTADPVPDAEGSVTVAVEATDPLPEQEGESSQSQVITLTT

NDSEEIGTAQAG*

>Pi9-Type03

MAETVLSMARSLVGSAISKAASAAANETSLLLGVEKDIWYIKDELKTMQAFLRAAEVMKK

KDELLKVWAEQIRDLSYDIEDSLDEFKVHIESQTLFRQLVKLRERHRIAIRIHNLKSRVE

EVSSRNTRYNLVEPISSGTEDDMDSYAEDIRNQSARNVDEAELVGFSDSKKRLLEMIDTN

ANDGPAKVICVVGMGGLGKTALSRKIFESEEDIRKNFPCNAWITVSQSFHRIELLKDMIR

QLLGPSSLDQLLQELQGKVVVQVHHLSEYLIEELKEKRYFVVLDDLWILHDWNWINEIAF

PKNNKKGSRIVITTRNVDLAEKCATASLVYHLDFLQMNDAITLLLRKTNKNHEDMESNKN

MQKMVERIVNKCGRLPLAILTIGAVLATKQVSEWEKFYEHLPSELEINPSLEALRRMVTL

GYNHLPSHLKPCFLYLSIFPEDFEIKRNRLVGRWIAEGFVRPKVGMTTKDVGESYFNELI

NRSMIQRSRVGIAGKIKTCRIHDIIRDITVSISRQENFVLLPMGDGSDLVQENTRHIAFH

GSMSCKTGLDWSIIRSLAIFGDRPKSLAHAVCPDQLRMLRVLDLEDVTFLITQKDFDHIA

LLCHLKYLSIGYSSSIYSLPRSIGKLQGLQTLNMPSTYIAALPSEISKLQCLHTLRCIGQ

FHYDNFSLNHPMKCITNTICLPKVFTPLVSRDDRAKQIAELHMATKSCWSESFGVKVPKG

IGKLRDLQVLEYVDIRRTSSRAIKELGQLSKLRKLGVTTNGSTKEKCKILYAAIEKLSSL

QSLHVDAVLFSGIIGTLECLDSISSPPPLLRTLRLNGSLEEMPNWIEQLTHLKKFDLRRS

KLKEGKTMLILGALPNLMVLYLYRNAYLGEKLVFKTGAFPNLRTLCIYELDQLREIRFED

GSSPLLEKIEIGKCRLESGIIGIIHLPKLKEIPITYGSKVAGLGQLEGEVNTHPNRPVLL

MYSDRRYHDLGAEAEGSSIEVQTADPVPDAEGSVTVAVEATDPLPEQEGESSQSQVITLT

TNDSEEIGTAQAG*

>Pi9-Type04

MAETVLSMARSLVGSAISKAASAAANETSLLLGVEKDIWYIKDELKTMQAFLRAAEVMKK

KDELLKVWAEQIRDLSYDIEDSLDEFKVHIESQTLFRQLVKLRERHRIAIRIHNLKSRVE

EVSSRNTRYNLVEPISSDTEDDMDSYAEDIRNQSARNVDEAELVGFSDSKKRLLEMIDTN

ANDGPAKVICVVGMGGLGKTALSRKIFESEEDIRKNFPCNAWITVSQSFHRIELLKDMIR

QLLGPSSLDQLLQELQGKVVVQVHHLSEYLIEELKEKRYFVVLDDLWILHDWNWINEIAF

PKNNKKGSRIVITTRNVDLAEKCATASLVYHLDFLQMNDAITLLLRKTNKNHEDMESNKN

MQKMVERIVNKCGRLPLAILTIGAVLATKQVSEWEKFYEHLPSELEINPSLEALS*MVTL

GYNHLPSHLKPCFLYLSIFPEDFEIKRNRLVGRWIAEGFVRPKVGMTTKDVGESYFNELI

NRSMIQRSRVGIAGKIKTCRIHDIIRDITVSISRQENFVLLPMGDGSDLVQENTRHIAFH

GSMSCKTGLDWSIIRSLAIFGDRPKSLAHAVCPDQLRMLRVLDLEDVTFLITQKDFDRIA

LLCHLKYLSIGYSSSIYSLPRSIGKLQGLQTLNMPSTYIAALPSEISKLQCLHTLRCIGQ

FHYDNFSLNHPMKCITNTICLPKVFTPLVSRDDRAKQIAELHMATKSCWSESFGVKVPKG

IGKLRDLQVLEYVDIKRTSSRAIKELGQLSKLRKLGVITKGSTKEKCKILYAAIEKLSSL

QYLYVNAALLSDIETLECLDSISSPPPLLSTLRLNGSLEEMPNWIEQLTHLKKFYLRRSK

LKEGKTMLILGALPNLMVLYLYRNAYLGEKLVFKTGAFPNLRTLCIYELDQLREIRFEDG

SSPLLEKIEIGKCRLESGIIGIIHLPKLKEIPITYGSKVAGLGQLEGEVNAHPNRPVLLM

YSDRRYHDLGAEAEGSSIEVQTADPVPDAEGSVTVAVEATDPLPEQEGESSQSQVITLTT

NDSEEIGTAQAG*

>Pi9-Type05

MAETVLSMARSLVGSAISKAASAAANETSLLLGVEKDIWYIKDELKTMQAFLRAAEVMKK

KDELLKVWAEQIRDLSYDIEDSLDEFKVHIESQTLFRQLVKLRERHRIAIRIHNLKSRVE

EVSSRNTRYNLVEPISSGTEDDMDSYAEDIRNQSARNVDEAELVGFSDSKKRLLEMIDTN

ANDGPAKVICVVGMGGLGKTALSRKIFESEEDIRKNFPCNAWITVSQSFHRIELLKDMIR

QLLGPSSLDQLLHELQGKVVVQVHHLSEYLIEELKEKRYFVVLDDLWILHDWNWINEIAF

PKNNKKGSRIVITTRNVDLAEKCATASLVYHLDFLQMNDAISLLLRKTNKNHEDMESNKN

MQKMVERIVNKCGRLPLAILTIGAVLATKQVSEWEKFYEQLPSELEINPSLEALRRMVTL

GYNHLPSHLKPCFLYLSIFPEDFEIQRNRLVGRWIAEGFVRPKVGMTTKDVGESYFNELI

NRSMIQRSRVGTAGKIKTCRIHDIIRDITVSISRQENFVLLPMGDGSDLVQENTRHIAFH

GSMSCKTGLDWSIIRSLAIFGDRPKSLAHAVCPDQLRMLRVLDLEDVTFLITQKDFDRIA

LLCHLKYLSIGYSSSIYSLPRSIGKLQGLQTLNMSSTYIAALPSEISKLQCLHTLRCIRE

LEFDNFSLNHPMKCITNTICLPKVFTPLVSRDNRAKQIAEFHMATKSFWSESFGVKVPKG

IGKLRDLQVLEYVDIRRTSSRAIKELGQLSKLRKLAVITKGSTKEKCKILYAAIEKLSSL

QSLYMNAALLSDIETLECLDSISSPPPLLRTLGLNGSLEEMPNWIEQLTHLKKFNLWSSK

LKEGKNMLILGALPNLMFLSLYHNSYLGEKLVFKTGAFPNLRTLVIFNLDQLREIRFEDG

SSPQLEKIEISCCRLESGIIGIIHLPRLKEISLEYKSKVARLGQLKGEVNTHPNRPVLRM

DSDRRDHDLGAEAEGSSIEVQTADPVPDAQGSVTVAVEATDPLPEQEGESSQSQVITLTT

NDSEEIGTAQAG*

>Pi9-Type06

MAETVLSMARSLVGSAISKAASAAANETSLLLGVEKDIWYIKDELKTMQAFLRAAEVMKK

KDELLKVWAEQIRDLSYDIEDSLDEFKVHIESQTLFRQLVKLRERHRIAIRIHNLKSRVE

EVSSRNTRYNLVEPISSGTEDDMDSYAEDIRNQSARNVDEAELVGFSDSKKRLLEMIDTN

ANDGPAKVICVVGMGGLGKTALLRKIFESEEDIRKNFPCIAWITVSQSFHRIELLKDMIR

QLLGPSSLDQLLQELQGKVVVQVHHLSEYLIEELKEKRYFVILDDLWILHDWNWINEIAF

PKNNKKGSRIVITTRNVDLAEKCATASLVYHLDFLQMNDAITLLLRKTNKNHEDMESNKN

MQKMVERIVNKCGRLPLAILTIGAVLATKHVSEWEKFYEQLPSELEINPSLEALRRMVTL

GYNHLPSHLKPCFLYLSIFPEDFEIKRNRLVGRWIAEGFVRPKVGMTTKDVGESYFNELI

NRSMIQRSRVGIAGKIKTCRIHDIIRDITVSISRQENFVLLPMGDGSDLVQENTRHIAFH

GSMSCKTGLDWSIIRSLAIFGDRPKSLAHAVCPDQLRMLRVLDLEDVTFLITQKDFDRIA

LLCHLKYLSIGYSSSIYSLPRSIGKLQGLQTLNMPSTYIAALPSEISKLQCLHTLRCSRK

FVYDNFSLNHPMKCITNTICLPKVFTPLVSRDDRAIQIAELHMATKSCWSESFGVKVPKG

IGKLRDLQVLEYVDIRRTSSRAIKELGQLSKLRKLGVTTNGSTKEKCKILYAAIEKLSSL

QSLHVDAVLFSGIIGTLECLDSISSPPPLLRTLGLNGILEEMPNWIEQLTHLKKFYLLSS

KLKEGKTMLILGALPNLMVLYLYWNAYLGEKLVFKTGAFPNLRTLHIYESDQLREMRFED

GSSPLLEKIEIFRCRLESGIIGIIHLPRLKEISLEYKSKVARLGQLEGEVSTHPNRPVLR

MDSDRRDHDLGAEAEGSSIEVQTADPVPDAQGSVTVAVEATDPLPEQEGESSQSQVIMLT

TNDSEEIGTAQAG*

>Pi9-Type07

MAETVLSMARSLVGSAISKAASAAANETSLLLGVEKDIWYIKDELKTMQAFLRAAEVMKK

KDELLKVWAEQIRDLSYDIEDSLDEFKVHIESQTLFRQLVKLRERHRIAIRIHNLKSRVE

EVSSRNTRYNLVEPISSGTEDDMDSYAEDIRNQSARNVDEAELVGFSDSKKRLLEMIDTN

ANDGPAKVICVVGMGGLGKTALSRKIFESEEDIRKNFPCNAWITVSQSFHRIELLKDMIR

QLLGPSSLDQLLQELQGKVVVQVHHLSEYLIEELKEKRYFVVLDDLWILHDWNWINEIAF

PKNNKKGSRIVITTRNVDLAEKCATASLVYHLDFLQMNDAITLLLRKTNKNHEDMESNKN

MQKMVERIVNKCGRLPLAILTIGAVLATKQVSEWEKFYEHLPSELEINPSLEALRRMVTL

GYNHLPSHLKPCFLYLSIFPEDFEIKRNRLVGRWIAEGFVRPKVGMTTKDVGESYFNELI

NRSMIQRSRVGIAGKIKTCRIHDIIRDITVSISRQENFVLLPMGDGSDLVQENTRHIAFH

GSMSCKTGLDWSIIRSLAIFGDRPKSLAHAVCPDQLRMLRVLDLEDVTFLITQKDFDHIA

LLCHLKYLSIGYSSSIYSLPRSIGKLQGLQTLNMPSTYIAALPSEISKLQCLHTLRCSRK

FVYDNFSLNHPMKCITNTICLPKVFTPLVSRDDRAKQIAELHMATKSCWSESFGVKVPKG

IGKLRDLQVLEYVDIRRTSSRAIKELGHLSKLRKLGVITKGSTKEKCKILYAAIEKLSSL

QSLYVNAALLSDIETLECLDSISSPPPLLRTLGLNGSLEEMPNWIEQLTHLKKIYLLRSK

LKEGKTMLILGALPNLMVLYLYRNAYLGEKLVFKTGAFPNLRTLCIYELDQLREIRFEDG

SSPLLEKIEIGKCRLESGIIGIIHLPKLKEIPITYGSKVAGLGQLEGEVNTHPNRPVLLM

YSDRRYHDLGAEAEGSSIEVQTADPVPDAEGSVTVAVEATDPLPEQEGESSQSQVITLTT

NDSEEIGTAQAG*

>Pi9-Type08

MAETVLSMARSLVGSAISKAASAAANETSLLLGVEKDIWYIKDELKTMQAFLRAAEVMKK

KDELLKVWAEQIRDLSYDIEDSLDEFKVHIESQTLFRQLVKLRERHRIAIRIHNLKSRVE

EVSSRNTRYNLVEPISSGTEDDMDSYAEDIRNQSARNVDEAELVGFSDSKKRLLEMIDTN

ANDGPAKVICVVGMGGLGKTALSRKIFESEEDIRKNFPCNAWITVSQSFHRIELLKDMIR

QLLGPSSLDQLLHELQGKVVVQVHHLSEYLIEELKEKRYFVVLDDLWILHDWNWINEIAF

PKNNKKGSRIVITTRNVDLAEKCATASLVYHLDFLQMNDAISLLLRKTNKNHEDMESNKN

MQKMVERIVNKCGRLPLAILTIGAVLATKQVSEWEKFYEQLPSELEINPSLEALRRMVTL

GYNHLPSHLKPCFLYLSIFPEDFEIQRNRLVGRWIAEGFVRPKVGMTTKDVGESYFNELI

NRSMIQRSRVGTAGKIKTCRIHDIIRDITVSISRQENFVLLPMGDGSDLVQENTRHIAFH

GSMSCKTGLDWSIIRSLAIFGDRPKSLAHAVCPDQLRMLRVLDLEDVTFLITQKDFDRIA

LLCHLKYLSIGYSSSIYSLPRSIGKLQGLQTLNMSSTYIAALPSEISKLQCLHTLRCIRE

LEFDNFSLNHPMKCITNTICLPKVFTPLVSRDNRAKQIAEFHMATKSFWSESFGVKVPKG

IGKLRDLQVLEYVDIRRTSSRAIKELGQLSKLRKLAVITKGSTKEKCKILYAAIEKLSSL

QSLYMNAALLSDIETLECLDSISSPPPLLRTLGLNGSLEEMPNWIEQLTHLKKFNLWSSK

LKEGKNMLILGALPNLMFLSLYHNSYLGEKLVFKTGAFPNLRTLVIFNLDQLREIRFEDG

SSPQLEKIEISCCRLESGIIGIIHLPRLKEISLEYKSKVARLGQLKGEVNTHPNRPVLRM

DSDRRDHDLGAEAEGSSIEVQTADPVPDAQGSVTVAVEATDPLPEQEGESSQSQVITLTT

NDSEEIGTAQAG*

>Pi9-Type09

MAETVLSMARSLVGSAISKAASAAANETSLLLGVEKDIWYIKDELKAMQAFLRAAEVMKK

KDELLKVWAEQICDLSYDIEDSLDEFKVHIESQNLFRQMVKLRERHRIAIRIHNLKSRVE

EVSSRNTRYSLVKPISSSTEDDIDSYAEDIRNLSARNVDEAELVGFSDSKKRLLEMIDTN

ANDGPAKVICVVGMGGLGKTALSRKIFESEEDIRKNFPCNAWITVSQSFHRIELLKDMIR

QLLGPISLNLLLKELQGKVVVQVHHLSEYLLEELKEKRYFVVLDDLWFLHDWNWINDIAF

PKNNKMGSRIVITTRSVDLAEKCATASLVYHLDFLQMNDAITLLLRKTNKKHEDMESNKN

MQNMVERIVNKCGRLPLAILTIGAVLATKHVSEWEKFYEKLPSELEINPSLEALRRMVIL

GYNHLPSHLKPCFLYLSIFPEDFEIKRNRLVGRWIAEGFVRPQVGMMTKDVGESYFNELI

SRSMIQRSRVGIAGKIQSCRVHDIIRDITVSISRQENFVLLPMGDGSDLVQENTRHIAFH

GSMSCKTGLDWSIIRSLAIFGGPKSLAHAVCPDQLRMLRVLDLEDVTFLITQKDFDRIAL

LCHLKYLSIGYSSCIYSLPRSIGKLQGLQTLNMPSTYIAALPSEISKLQCLHTLRCIREF

HYDNFSLNHPMKCITNTICLPKVFTPLVSRDDRAKQIAEFHIATKRFWSESFGVKVPKGI

GKLRDLQVLEYVDIRRTSSRAIKELGQLSKLRKLGVITKGSTKEKCKILYAAIEKLSSLQ

SLYVNAALLSDIETFECLDSISSPPPLLRTLRLNGSLEEMPNWIEQLTHLKKIYLLKSKL

KEGKTMLILGALPNLMVLHLYRNAYLGEKLVFKTGAFPNLRTLRIYELDQLREMRFEDGS

SPLLEKIEIGNCRLESGIIGIIHHPKLKEISIRYGSKVAGLGQLEGEVNTHPNRPVLRMD

SDRRDHDLGAEAEGSSIEVQTADPVPDAE*SVTVAVEATDPLPEQEGESSQSQVITLTTN

DSEEIGTAQAG*

>Pi9-Type10

MAETVLSMARSLVGSAISKAASAAANETSLLLGVEKDIWYIKDELKTMQAFLRAAEVMKK

KDELLKVWAEQIRDLSYDIEDSLDEFKVHIESQTLFRQLVKLRERHRIAIRIHNLKSRVE

EVSSRNTRYNLVEPISSGTEDDMDSYAEDIRNHSARNVDEAELVGFSDSKKRLLEMIDTN

ANDGPAKVICVVGMGGLGKTALSRKIFESEEDIRKNFPCIAWITVSQSFHRIELLKDMIR

QLLGPSSLDQLLQELQGKVVVQVHHLSEYLIEELKEKRYFVILDDLWILHDWNWINEIAF

PKNNKKGSRIVITTRNVDLAEKCATASLVYHLDFLQMNDAITLLLRKTNKNHEDMESNKN

MQKMVERIVNKCGRLPLAILTIGAVLATKHVSEWEKFYEQLPSELEINPSLEALRRMVTL

GYNHLPSHLKPCFLYLSIFPEDFEIKRNRLVGRWIAEGFVRPKVGMTTKDVGESYFNELI

NRSMIQRSRVGIAGKIKTCRIHDIIRDITVSISRQENFVLLPMGDGSDLVQENTRHIAFH

GSMSCKTGLDWSIIRSLAIFGDRPKSLAHAVCPDQLRMLRVLDLEDVTFLITQKDFDRIA

LLCHLKYLSIGYSSSIYSLPRSIGKLQGLQTLNMLRTYIAALPSEISKLQCLHTLRCIGQ

FPYDNFSLNHPMKCITNTICLPKVFTPLVSRDDRAKQIAELHMATKSCWSESFGVKVPKG

IGKLRDLQVLEYVDIRRTSSRAIKELGQLSKLRKLGVITKGSTKEKCKILYAAIEKLSSL

QSLYVNAALLSDIETLECLDSISSLPPPLLRTLGLNGSLEEMPNWIEQLTHLKKFYLLGS

KLKEGKTMLILGALPNLMVLYLYGNAYLGEKLVFKTGAFPNLRTLRIYELAQLREMRFED

GSSPLLEKIEISCCRLESGIIGIIHLPRLKEISLEYKSKVARLGQLEGEVNTHPNRPVLR

MDSDRRDHDLGAEAEGSSIEVQTADPVPDAEGSVTVAVEATDPLPEQEGESSQSQVITLT

TNDSEEIGTAQAG*

>Pi9-Type11

MAETVLSMARSLVGSAISKAASAAANETSLLLGVEKDIWYIKDELKTMQAFLRAAEVMKK

KDELLKVWAEQIRDLSYDIEDSLDEFKVHIESQTLFRQLVKLRERHRIAIRIHNLKSRVE

EVSSRNTRYNLVEPISSGTEDDMDSYAEDIRNQSARNVDEAELVGFSDSKKRLLEMIDTN

ANDGPAKVICVVGMGGLGKTALSRKIFESEEDIRKSFPCIAWITVSQSFHRIELLKDMIR

QLLGPSSLDQLLQELQGKVVVQVHHLSEYLIEELKEKRYFVILDDLWILHDWNWINEIAF

PKNNKKGSRIVITTRNVDLAEKCATASLVYHLDFLQMNDAITLLLRKTNKNHEDMESNKN

MQKMVERIVNKCGRLPLAILTIGAVLATKHVSEWEKFYEQLPSELEINPSLEALRRMVTL

GYNHLPSHLKPCFLYLSIFPEDFEIKRNRLVGRWIAEGFVRPKVGMTTKDVGESYFNELI

NRSMIQRSRVGIAGKIKTCRIHDIMRDITVSISRQENFVLLPMGDGSDLVQENTRHIAFH

GSMSCKTGLDWSIIRSLAIFGDRPKSLAHAVCPDQLRMLRVLDLEDVTFLITQKDFDRIA

LLCHLKYLSIGYSSSIYSLPRSIGKLQGLQTLNMPSTYIAALPSEISKLQCLHTLRCIGQ

FPYDNFSLNHPMKCITNTICLPKVFTPLVSRDDRAKQIAELHMATKSCWSESFGVKVPKG

IGKLRDLQVLEYVDIRRTSSRAIKELGQLSKLRKLGVITKGSTKEKCKILYAAIEKLSSL

QSLYVNAALLSDIETLECLDSISSPPPLLWTLVLIGSLEEMPNWIEQLTHLKKFYLLSSK

LKEGKTMLILGALPNLMVLYLYWNAYLGEKLVFKTGAFPNLRTLHIYKSDQLREMRFEDG

SSPLLEKIEISCCRLESGIIGIIHLPRLKEISLEYKSKVARLGQLEGEVNTHPNRPVLRM

DSDRRDHDLGAEAEGSSIEVQTADPVPDAQGSVTVAVEATDPLPEQEGESSQSQVITLTT

NDSEEIGTAQAG*

>Pi9-Type12

MAETVLSMARSLVGSAISKAASAAANETSLLLGVEKDIWYIKDELKTMQAFLRAAEVMKK

KDELLKVWAEQIRDLSYDIEDSLDEFKVHIESQTLFRQLVKLRERHRIAIRIHNLKSRVE

EVSSRNTRYNLVEPISSGTEDDMDSYAEDIRNQSARNVDEAELVGFSDSKKRLLEMIDTN

ANDGPAKVICVVGMGGLGKTALSRKIFESEEDIRKNFPCIAWITVSQSFHRIELLKDMIR

QLLGPSSLDQLLQELQGKVVVQVHHLSEYLIEELKEKRYFVILDDLWILHDWNWINEIAF

PKNNKKGSRIVITTRNVDLAEKCATASLVYHLDFLQMNDAITLLLRKTNKNHEDMESNKN

MQKMVERIVNKCGRLPLAILTIGAVLATKHVSEWEKFYEQLPSELEINPSLEALRRMVTL

GYNHLPSHLKPCFLYLSIFPEDFEIKRNRLVGRWIAEGFVRPKVGMTTKDVGESYFNELI

NRSMIQRSRVGIAGIIKTCRIHDIIRDITVSISRQENFVLLPMGDGSDLVQENTRHIAFH

GSMSCKTGLDWSIIRSLAIFGDRPKSLAHAVCLDQLRMLRVLDLEDVTFLITQKDFDRIA

LLCHLKYLSIGYSSSIYSLPRSIGKLQGLQTLNMLRTYIAALPSEISKLQCLHTLRCSRK

FVYDNFSLNHPMKCITNTICLPKVFTPLVSRDDRAKQIAELHMATKSCWSESFGVKVPKG

IGKLRDLQVLEYVDIRRTSSRAIKELGQLSKLRKLGVITKGSTKEKCKILYAAIEKLSSL

QSLYVNAALLSDIETLECLDSISSPPPLLWTLGLNGSLEEMPNWIEQLTHLKKIYLLRSK

LKEGKTMLILGALPNLMVLYLYWNAYLGEKLVFKTGAFPNLRTLRIYELDQLREMRFEDG

SSPLLEKIEISCCRLESGIIGIIHLPRLKEISLEYKSKVARLGQLEGEVNTHPNRPVLRM

DSDRRDHDLGAEAEGSSIEVQTADPVPDAEGSVTVAVEATDPLPEQEGESSQSQVITLTT

NDSEEIGTAQAG*

>Pi9-Type13

MAETVLSMARSLVGSAISKAASAAANETSLLLGVEKDIWYIKDELKTMQAFLRAAEVMKK

KDELLKVWAEQIRDLSYDIEDSLDEFKVHIESQTLFRQLVKLRERHRIAIRIHNLKSRVE

EVSSRNTRYNLVEPISSGTEDDMDSYAEDIRNQSARNVDEAELVGFSDSKKRLLEMIDTN

ANDGPAKVICVVGMGGLGKTALSRKIFESEEDIRKNFPCIAWITVSQSFHRIELLKDMIR

QLLGPSSLDQLLQELQGKVVVQVHHLSEYLIEELKEKRYFVILDDLWILHDWNWINEIAF

PKNNKKGSRIVITTRNVDLAEKCATASLVYHLDFLQMNDAITLLLRKTNKNHEDMESNKN

MQKMVERIVNKCGRLPLAILTIGAVLATKHVSEWEKFYEQLPSELEINPSLEALRRMVTL

GYNHLPSHLKPCFLYLSIFPEDFEIKRNRLVGRWIAEGFVRPKVGMTTKDVGESYFNELI

NRSMIQRSRVGIAGKIKTCRIHDIIRDITVSISRQENFVLLPMGDGSDLVQENTRHIAFH

GSMSCKTGLDWSIIRSLAIFGDRPKSLAHAVCPDQLRMLRVLDLEDVTFLITQKDFDRIA

LLCHLKYLSIGYSSSIYSLPRSIGKLQGLQTLNMPSTYIAALPSEISKLQCLHTLRCSRK

FVYDNFSLNHPMKCITNTICLPKVFTPLVSRDDRAIQIAELHMATKSCWSESFGVKVPKG

IGKLRDLQVLEYVDIRRTSSRAIKELGQLSKLRKLGVTTNGSTKEKCKILYAAIEKLSSL

QSLHVDAVLFSGIIGTLECLDSISSPPPLLRTLGLNGILEEMPNWIEQLTHLKKFYLLSS

KLKEGKTMLILGALPNLMVLYLYWNAYLGEKLVFKTGAFPNLRTLHIYESDQLREMRFED

GSSPLLEKIEIFRCRLESGIIGIIHLPRLKEISLEYKSKVARLGQLEGEVSTHPNRPVLR

MDSDRRDHDLGAEAEGSSIEVQTADPVPDAQGSVTVAVEATDPLPEQEGESSQSQVIMLT

TNDSEEIGTAQAG*
